# Secondary metabolites from the Endophytic fungi *Fusarium decemcellulare* F25 and their antifungal activities

**DOI:** 10.3389/fmicb.2023.1127971

**Published:** 2023-02-01

**Authors:** Ziwei Song, Yan Jun Sun, Shuangyu Xu, Gang Li, Chunmao Yuan, Kang Zhou

**Affiliations:** ^1^School of Pharmaceutical Sciences, Guizhou University, Guiyang, China; ^2^Key Laboratory of Plant Resource Conservation and Germplasm Innovation in Mountainous Region, Ministry of Education, Guizhou University, Guiyang, China; ^3^Department of Natural Medicinal Chemistry and Pharmacognosy, School of Pharmacy, Qingdao University, Qingdao, China; ^4^State Key Laboratory of Functions and Applications of Medicinal Plants, Guizhou Medical University, Guiyang, China

**Keywords:** *Fusarium decemcellulare* F25, secondary metabolites, isocoumarins, pyrrolidinones, antifungal activities

## Abstract

Seven new compounds, including three isocoumarins (**1**–**3**), three pyrrolidinone derivatives (**8**–**10**), and one pentaene diacid (**15**), together with 13 known compounds, were isolated from the rice culture of the endophytic fungus *Fusarium decemcellulare* F25. Their structures and stereochemistry were established using HRESIMS, NMR, electronic circular dichroism (ECD) calculations, and single-crystal X-ray diffraction. The possible biosynthetic pathways for compounds **1–3** and **8**–**10** were proposed. The antifungal efficacies of compounds **1 – 20** were evaluated against *Colletotrichum musae*, and compounds **13**, **14**, and **17** exhibited inhibitory activities against *C. musae* with MIC values of 256, 64 and 128 μg/mL, respectively.

## 1. Introduction

Endophytic fungi are microorganisms that asymptomatically colonize living tissues of healthy plants (Li and Lou, 2018). Their complex interactions with the plant host, other organisms, and the external environment result in the production of secondary metabolites that are often characterized by diverse structures and biological activities (Zhang et al., 2006; Gao et al., 2018; Gupta et al., 2019).

*Mahonia fortunei* is a traditional Chinese medicinal plant, and its root, stem, and leave can be used as medicine for treating bacterial infection, pneumoconiosis, psoriasis, and cough (Zhang et al., 2015). Fungal endophytes from this medicinal plant have afforded many bioactive novel natural products, indicating that mining fungi from this host is an effective strategy for obtaining potential lead compounds (Li et al., 2015, 2016; Wang et al., 2019; Tian et al., 2021). Specifically, an antibacterial tetracyclic triterpenoid with a unique aromatic B-ring, and a cytochalasan with a new 6/6/5-fused tricyclic core skeleton were isolated from *M. fortunei*-derived endophytic fungi (Li et al., 2015; Wang et al., 2019).

In our continuous research on fungal endophytes from *M. fortunei*, endophytic *Fusarium decemcellulare* F25 was obtained. Study on secondary metabolites from *F. decemcellulare* is relatively less. Li et al. reported three cyclic pentapeptides and an antifungal cyclic lipopeptide from an endophytic fungus, *F. decemcellulare* LG53 (Li et al., 2016). The well-known shikimic acid can be produced by *F. decemcellulare* harboring in the fruits of the plant *Flacourtia inermis* (Qader et al., 2018). Under guidance of ^1^H NMR, 12 polypropionate derivatives were isolated from a marine-derived fungus *F. decemcellulare* SYSUMS6716, and two compounds, decempyrones C and J, exhibited potent anti-inflammatory activity and inhibitory activity against protein tyrosine phosphatase A (Guo et al., 2021).

In the specific ecological niche, endophytic fungi could coevolve with associated organisms, such as other endophytic fungi and environmental pathogens. This usually made fungal endophytes produce antifungal compounds for chemical defense (Li et al., 2015). Therefore, the antifungal activity of the ethyl acetate (EtOAc) extract of *F. decemcellulare* F25 was investigated. Its ethyl acetate extract showed a significant inhibition activity against *Colletotrichum musae* ACCC 31244, revealing the production of bioactive metabolites and being worth for chemical investigation.

**Figure 1:**
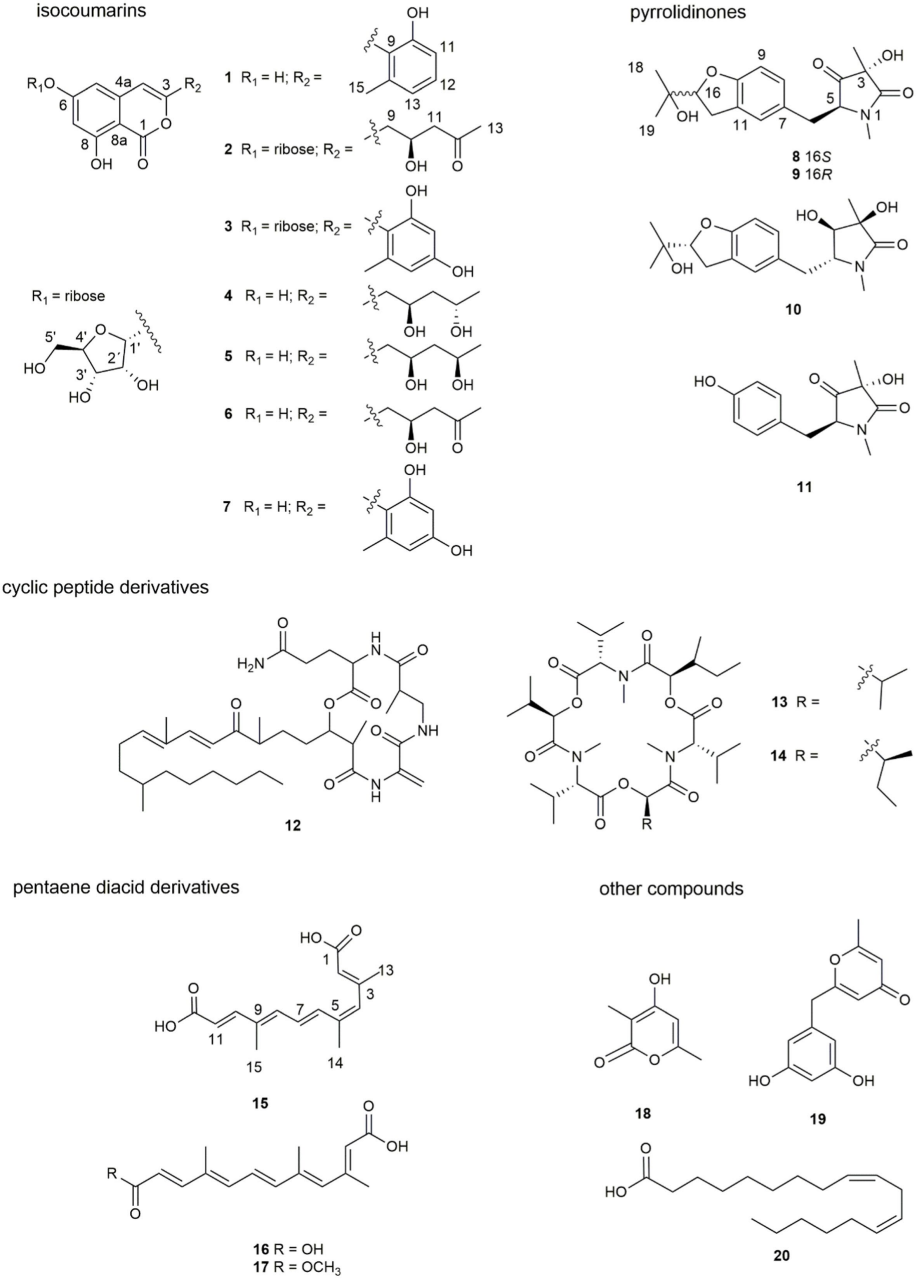
Structures of compounds **1** – **20**.

Further isolation led to the identification of three new isocoumarins (**1**–**3**), three new pyrrolidinones (**8**–**10**), and one new pentaene diacid (**15**), together with 13 known compounds from the endophytic fungus *F. decemcellulare* F25 (Figure 1). Herein, we report their isolation, identification, and biological activity, together with the proposed biosynthetic pathway.

## 2. Materials and methods

### 2.1. General experimental procedures

Optical rotations were acquired on a JASCOP-1020 polarimeter. ECD data were measured on a Chirascan spectropolarimeter. IR spectra were measured on PerkinElmer infrared spectrophotometer. 1D and 2D NMR spectra were recorded on Bruker Avance 400 or 600 DRX spectrometers in acetone-*d*_6_, methanol-*d*_4_, DMSO-*d*_6_ and chloroform-*d*. Column chromatography (CC) was performed on silica gel (200**–**300 mesh; Qingdao Marine Chemical Plant Branch., China), RP-C18 (ODS-A, 50 μm, YMC, Kyoto, Japan), or Sephadex LH-20 (100–200 mesh; Beijing Solarbio Technology Co., Ltd., China). Plates precoated with silica gel GF254 (Rushan, Shandong Sun Desiccant Co., Ltd.) were used for thin layer chromatography (TLC). An Agilent HPLC series 1260 and Shimadzu LC-20AR were used for analysis and isolation. For analysis, an Agilent Eclipse XDB-C18 column (4.6 × 150 mm, 5 μm) was used. The isolation was achieved on an Agilent semi-preparative Eclipse XDB-C18 column (9.4 × 250 mm, 5 μm). HPLC-MS data were acquired on an Agilent 1260 Series system coupled with an Agilent Accurate-Mass-Q-TOF MS 6520 system equipped with an Electrospray ionization (ESI) source.

### 2.2. Fungal material

The endophytic fungus *F. decemcellulare* F25 was isolated from the stem of the Chinese medicinal plant *M. fortunei* collected from Qingdao, People’s Republic of China. The fungal strain was deposited in 20% glycerol at −80°C in the school of Pharmaceutical Sciences, Guizhou University, Guizhou, China. The endophytic fungus was identified as *F. decemcellulare* by the analysis of internal transcribed spacer (ITS) region of the rDNA (GenBank No. OQ001346).

### 2.3. Fermentation and extraction

The fungal strain *F. decemcellulare* F25 was cultured on potato dextrose agar (PDA) media for a week at 28 ± 2°C. A week-old culture plate was cut into small pieces under aseptic conditions, and were then inoculated into 394 flasks (300 mL) each containing 40 g of rice, 0.12 g of peptone, and 60 mL of water. The cultures were incubated at 28 ± 2°C for 40 days. Afterward, the whole cultures were extracted with ethyl acetate by sonication under ice bath conditions for three times. Then the EtOAc solution was collected and evaporated to dryness, affording 352.2 g of brown extracts. After suspension of the crude extract in water, petroleum ether and EtOAc were used to extract 294.6 g and 50.0 g of the corresponding organic phase, respectively.

### 2.4. Isolation and purification

The EtOAc extract was fractionated by column chromatography (CC) on ODS eluting with a gradient of acetonitrile (CH_3_CN)/H_2_O (0:100; 3:7; 5:5; 7:3; 100:0, *v/v*, each 8 L) to give eight fractions (Fr. A**–**Fr. H).

Fraction A was applied to semi-preparative HPLC to yield compounds **11** (*t*_R_ 22.1 min, 53.3 mg), **4** (*t*_R_ 38.2 min, 8.9 mg), **5** (*t*_R_ 39.1 min, 16.5 mg), **6** (*t*_R_ 40.3 min, 29.1 mg), and a mixture of **8** and **9** (*t*_R_ 29.2 min, 16.2 mg), eluting with a gradient of CH_3_CN in H_2_O from 40 to 65%. The above mixture was subjected to isolation on a chiral HPLC column to afford compounds **8** (*t*_R_ 25.1 min, 6.2 mg) and **9** (*t*_R_ 28.9 min, 5.9 mg). Fraction A (14.4 g) was also separated into 12 subfractions (A1 − A12) by CC on silica gel eluted by CH_2_Cl_2_/CH_3_OH (1:0, 40:1, 30:1, 20:1, 10:1, 5:1 and 0:1, v/v, each 7 L). Subfraction A3 was purified by semi-preparative HPLC with a gradient elution from 30 to 85% CH_3_CN in H_2_O to afford compounds **1** (*t*_R_ 33.0 min, 11.5 mg) and **18** (*t*_R_ 12.6 min, 16.4 mg). Subfraction A6 was then applied to semi-preparative HPLC with a gradient from 30 to 55% CH_3_CN in H_2_O as eluent to obtain compound **19** (*t*_R_ 14.2 min, 25.3 mg). Compound **10** (*t*_R_ 33 min, 27.8 mg) was purified by semi-preparative HPLC, eluting with a gradient of CH_3_CN in H_2_O from 40 to 65% (v/v) as eluent from subfraction A7. Compound **2** (*t*_R_ 17.5 min, 10.1 mg) was obtained from subfraction A8 using semi-preparative HPLC with a gradient elution from 30 to 85% CH_3_CN in H_2_O. Subfrcation A9 was separated by semi-preparative HPLC with a gradient elution from 30 to 75% CH_3_CN in H_2_O to yield compound **3** (*t*_R_ 27.0 min, 10.6 mg).

Fraction C was fractionated by CC on Sephadex LH-20 eluting with CH_3_OH/CH_2_Cl_2_ (1:1, v/v) to give five subfractions (C1 **–** C5). Subfraction C3 was purified by semi-preparative HPLC with a gradient elution from 45 to 50% CH_3_CN in H_2_O with 0.1% trifluoroacetic acid (TFA) to afford compounds **15** (*t*_R_ 38.1 min, 7.5 mg) and **16** (*t*_R_ 37.5 min, 10.3 mg). Fraction C4 was subjected to semi-preparative HPLC with a gradient elution from 35 to 65% CH_3_CN in H_2_O to yield compound **7** (*t*_R_ 16.0 min, 2.0 mg).

Fraction D was purified by semi-preparative HPLC eluting with gradient from 60 to 70% CH_3_CN in H_2_O with 0.1% TFA to offer compound **17** (*t*_R_ 33.5 min, 6.3 mg). Compound **12** (*t*_R_ 27.2 min, 6.2 mg) was obtained from fraction F by semi-preparative HPLC eluting with a gradient elution from 50 to 100% CH_3_CN in H_2_O. Compounds **13** (*t*_R_ 19.3 min, 17.5 mg), **14** (*t*_R_ 22.0 min, 17.7 mg), and **20** (*t*_R_ 28.5 min, 13.1 mg) were obtained from fraction G using semi-preparative HPLC with a gradient elution from 75 to 100% CH_3_CN in H_2_O.

### 2.5. Spectroscopic data of compounds

Compound (**1**), yellowish solid; LC-UV (CH_3_CN in H_2_O) *λ*_max_: 248, 330 nm; IR *ν*_max_: 3319, 2942, 2832, 1677, 1020 cm^−1^; ^1^H NMR (DMSO-*d*_6_, 400 MHz); and ^13^C NMR (DMSO-*d*_6_, 100 MHz) data, see Table 1; HRESIMS *m/z* 285.0762 [M + H]^+^ (calcd. For C_16_H_13_O_5_, 285.0757).

**Table 1:** ^1^H NMR (400 MHz, *δ* in ppm) and ^13^C NMR Data (100 MHz, *δ* in ppm) of 1 – 3 (DMSO-*d_6_*).

Position	**1**		**2**		**3**	
	*δ_C_*, type	*δ_H_* (*J* in Hz)	*δ_C_,* type	*δ_H_* (*J* in Hz)	*δ_C_,* type	*δ_H_* (*J* in Hz)
1	166.0, C		165.5, C		166,2, C	
3	150.8, C		154.9, C		151.7, C	
4	108.7, CH	6.69, s	105.9, CH	6.55, s	108.9, CH	6.67, s
4a	138.2, C		139.3, C		139.6, C	
5	103.3, CH	6.47,d (2.1)	103.3, CH	6.57, d (2.3)	103.8, CH	6.72, d (2.2)
6	165.7, C		164.2, C		164.3, C	
7	101.9, CH	6.38, d (2.1)	102.5, CH	6.64, d (2.3)	102.8, CH	6.61, d (2.2)
8	162.7, C		162.2, C		162.3, C	
8a	98.4, C		99.9, C		100.1, C	
9	120.0, C		40.9, CH_2_	2.60, m	111.5, C	
10	155.9, C		64.6, CH	4.28, m	157.3, C	
11	113.0, CH	6.75, d (8.3)	50.3, CH_2_	2.57, m	100.3, CH	6.23, d (2.2)
12	130.6, CH	7.18, t (8.3)	207.7, C		159.3,C	
13	120.6, CH	6.77, d (8.3)	30.5, CH_3_	2.11, s	108.4,CH	6.19, d (2.2)
14	139.6, C				139.3, C	
15	19.5, CH_3_	2.21, s			19.9, CH_3_	2.12, s
1’			100.0, CH	5.74, d (4.4)	100.0, CH	5.76, d (4.5)
2’			71.5, CH	4.10, m	71.6, CH	4.11, m
3’			69.2, CH	3.96, m	69.3, CH	3.93, m
4’			86.7, CH	3.96, m	86.9, CH	3.98, m
5’			61.4, CH_2_	3.48, m	61.5, CH_2_	3.49, m

Compound (**2**), yellowish solid; $\;{\left[\alpha \right]}_{D}^{23}$ +51.6 (*c* 0.68, CH_3_OH); LC-UV (CH_3_CN in H_2_O) *λ*_max_: 244, 276, 330 nm; IR *ν*_max_: 3330, 2925, 1681, 1642, 1625, 1572, 1357, 1237, 1163, 1018, 990 cm^−1^; ^1^H NMR (DMSO-*d*_6_, 400 MHz); and ^13^C NMR (DMSO-*d*_6_,100 MHz) data, see Table 1; HRESIMS *m/z* 411.1289 [M + H]^+^ (calcd. For C_19_H_23_O_10_, 411.1286).

Compound (**3**), yellowish solid; ${\left[\alpha \right]}_{D}^{23}$ +63.2 (*c* 0.56, CH_3_OH); LC-UV (CH_3_CN in H_2_O) *λ*_max_: 204, 236, 336 nm; IR *ν*_max_: 3310, 2917, 1678, 1616, 1570, 1505, 1470, 1400, 1158, 1024, 999 cm^−1^; ^1^H NMR (DMSO-*d*_6_, 400 MHz); and ^13^C NMR (DMSO-*d*_6_,100 MHz) data, see Table 1; HRESIMS *m/z* 433.1133 [M + H]^+^ (calcd. For C_21_H_21_O_10_, 433.1129).

Compound (**8**), colorless oil; ${\left[\alpha \right]}_{D}^{24}$ -22.7 (*c* 0.59, CH_3_OH); LC-UV (CH_3_CN in H_2_O) *λ*_max_: 200, 230, 286 nm; IR *ν*_max_: 3329, 2946, 2836, 1661, 1451, 1408, 1114, 1017 cm^−1 1^H NMR (DMSO-*d*_6_, 400 MHz) and ^13^C NMR (DMSO-*d*_6_,100 MHz) data, see Supplementary Table 1; HRESIMS *m/z* 334,1,649 [M + H]^+^ (calcd. For C_18_H_24_NO_5_, 334.1649).

Compound (**9**), colorless oil; ${\left[\alpha \right]}_{D}^{24}$ +8.23 (*c* 0.61, CH_3_OH); LC-UV (CH_3_CN in H_2_O) *λ*_max_: 200, 230, 286 nm; IR *ν*_max_: 3329, 2946, 2836, 1661, 1451, 1408, 1114, 1017 cm^−1^;^1^H NMR (DMSO-*d*_6_, 400 MHz); and ^13^C NMR (DMSO-*d*_6_,100 MHz) data, see Supplementary Table 1; HRESIMS *m/z* 334, 1,652 [M + H]^+^ (calcd. For C_18_H_24_NO_5_, 334.1649).

Compound (**10**), colorless oil; ${\left[\alpha \right]}_{D}^{23}$ -24.1 (*c* 2.25, CH_3_OH); LC-UV (CH_3_CN in H_2_O) *λ*_max_: 204, 228, 286 nm; IR *ν*_max_: 3336, 3286, 2905, 1644, 1427, 1367, 1334, 1314, 1164, 1053, 1030 cm^−1^; ^1^H NMR (DMSO-*d*_6_, 400 MHz); and ^13^C NMR (DMSO-*d*_6_,100 MHz) data, see Supplementary Table 1; HRESIMS *m/z* 336,1808 [M + H]^+^ (calcd. For C_18_H_26_NO_5_, 336.1805).

Compound (**15**), yellow powder; LC-UV (CH_3_CN in H_2_O) *λ*_max_: 220, 280, 360 nm; IR *ν*_max_: 3387, 2833, 1698, 1475, 1391, 1017 cm^−1^; ^1^H NMR (DMSO-*d*_6_, 400 MHz); and ^13^C NMR (DMSO-*d*_6_,100 MHz) data, see Supplementary Table 2; HRESIMS *m/z* 263.1277 [M + H]^+^ (calcd. For C_15_H_19_O_4_, 263.1278).

### 2.6. X-ray crystallographic analysis of compound 9

The crystal structure of compound **9** was obtained from the solution of CH_3_OH. A suitable crystal were collected on a Bruker APEX-II CCD Venture diffractometer using graphite-monochromated Mo K*α* radiation (*λ* = 0.710 73 Å) at 297 K. Absorption correction using equivalent reflctions was performed with the SADABS program. Crystallographic tables were constructed using Olex2 (Dolomanov et al., 2010**)**. The structure was solved with the Shelxt software package

(Sheldrick, 2015), and refined with the Shelxt refinement package using Least Squares minimization.

Crystal data for compound **9**: C_18_H_25_NO_6_ (*M* = 351.39 g/mol): triclinic, space group P1, *a* = 6.4597(7) Å, *b* = 7.5258(7) Å, *c* = 9.4129(7) Å, *α* = 95°, *β* = 100°, *γ* = 93°, *V* = 93.981(8) Å^3^, *Z* = 1, *T* = 297 K, *μ*(Mo Kα) = 0.098 mm^−1^, *F*(000) = 188, *ρ*_calc_ = 1.311 g/cm^3^; of the 8,205 reflections measured (4.44° ≤ 2*Θ* ≤ 50.01°), 2,823 were unique (*R*_int_ = 0.0922, *R*_sigma_ = 0.0810) which used in all calculations. The final *R*_1_ was 0.0596 (*I* > 2σ(*I*)), and *wR*_2_ was 0.1706 (all data).

### 2.7. Antifungal assay

Following our previously established methods (Wang et al., 2019; Tian et al., 2021), the crude extract of *F. decemcellulare* F25 was firstly evaluated for antifungal activity against five plant pathogens (*Colletotrichum musae* ACCC 31244, *Alternaria solani*, *Fusarium foetens*, *Fusarium mangiferae*, and *Lasiodiplodia pseudotheobromae*) by agar diffusion assay. The crude extract showed inhibitory activity against *C. musae* ACCC 31244, indicating the production of antifungal molecules. Further antifungal evaluation of pure compounds against *C. musae* ACCC 31244 was determined with the broth dilution method, and provided minimum inhibitory concentration (MIC) values. The cycloheximide was used as a positive control in parallel to reveal the comparative antifungal efficacy of compounds **1**–**20**.

## 3. Results and discussion

### 3.1. OSMAC screen and fermentation of *Fusarium decemcellulare* F25

The OSMAC (One Strain Many Compounds) approach refers to the activation of many silent gene clusters in microorganisms by altering the culture environment of the strain. This strategy maximizes the biosynthetic capacity of a microorganism that produces structurally diverse and biologically active secondary metabolites. The *F. decemcellulare* F25 was cultured on four different solid media including rice-based, soybean-based, corn-based, and czapek-dox agar (CDA) culture. Remarkably, HPLC chromatograms showed a number of peaks in rice-based culture, suggesting that the rice medium strongly triggered the production of secondary metabolites (Supplementary Figure 1).

### 3.2. Screening of antifungal activities of crude extract

The antifungal activities of crude extracts were evaluated against five plant pathogens. Compared with the positive control drug cycloheximide, it was found that, at the concentration of 40 μg/paper disk, the crude extract of *F. decemcellulare* F25 showed antifungal activity against the fungal *C. musae* (Supplementary Figure 2). Considering the abundant secondary metabolites and the antifungal activity of *F. decemcellulare* F25, this strain F25 was further subjected to chemical investigation.

### 3.3. Structural characterization of these isolated compounds

Seven new compounds, including three isocoumarins (**1**–**3**), three pyrrolidinone derivatives (**8**–**10**), and one pentaene diacid (**15**), together with 13 known compounds, were isolated from the rice culture of the endophytic fungus *Fusarium decemcellulare* F25.

**Figure 2:**
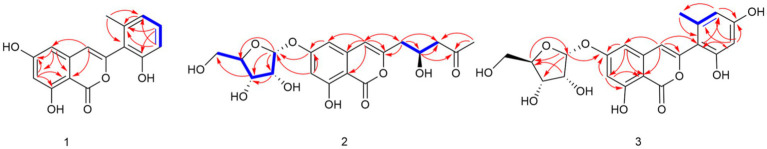
Key COSY (bold lines) and HMBC (arrows) correlations of compounds **1**–**3**.

Compound **1** was obtained as a yellowish solid. Its molecular formula C_16_H_12_O_5_ was established by the HRESIMS at *m/z* 285.0762 [M + H]^+^ (calcd. For 285.0757), implying eleven degrees of unsaturation. The presence of hydroxyl and carbonyl groups were implied by IR absorption bands at 3319 and 1677 cm^−1^, respectively. The ^1^H and ^13^C NMR data of **1** (Table 1) were highly similar to those of pleosporalone A (Cao et al., 2016), excepted for a proton at C-7 in **1** rather than a methyl group in pleosporalone A. In addition, the coupling constant between H-5 and H-7 (*J* = 2.1 Hz) proved that there is no methyl substitution at C-7 of **1**. The HMBC correlations (Figure 2) from H-5 (*δ*_H_ 6.47) to C-6 (*δ*_C_ 165.7), C-7 (*δ*_C_ 101.9), and C-8a (*δ*_C_ 98.4), and from H-7 (*δ*_H_ 6.38) to C-5 (*δ*_C_ 103.3), C-6 (*δ*_C_ 165.7), C-8 (*δ*_C_ 162.7), and C-8a (*δ*_C_ 98.4), together with the HSQC spectrum (*δ*_C_/*δ*_H_ 101.9/6.38 and 103.3/6.47 ppm) further confirmed that there is a proton attached to C-7. Final detailed analysis of the HSQC and HMBC spectra allowed the assignment for all proton and carbon resonances of **1**. Thus, the structure of compound **1** was assigned completely.

**Figure 3:**
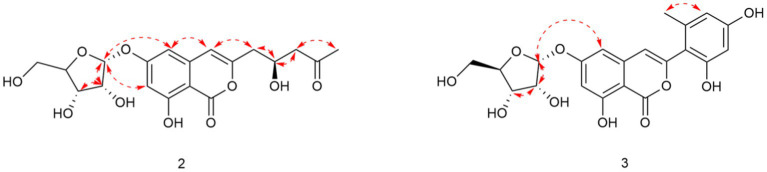
Key NOESY correlations of compounds **2** and **3**.

Compound **2** was obtained as a yellowish solid. Its molecular formula C_19_H_22_O_10_ was established by the HRESIMS at *m/z* 411.1289 [M + H]^+^ (calcd for 411.1286), implying nine degrees of unsaturation. The presence of hydroxyl and carbonyl groups were shown by IR absorption bands at 3330, 1681 cm^−1^, respectively. The attachment of sugar unit was determined to be ribose by comparison of ^1^H and ^13^C NMR data of compound **2** with those of daldiniside C. The coupling constant of the anomeric proton at *δ*_H_ 5.74 (1H, d, *J* = 4.4 Hz) indicated the ribose unit should be α-configured (Hu et al., 2014). Additionally, the pentose moiety was linked to C-6 proved by the correlation of H-1’/C-6 observed in the HMBC (Figure 2) experiment and H-1’/H-5, H-1’/H-7 in NOESY spectrum (Figure 3). The ^1^H NMR and ^13^C NMR data (Table 1) showed the presence of an isocoumarin unit in **2**, whose structure was the same as that of compound **6** ((−)-citreoisocoumarin)) (Yamamura et al., 1991) by comparison with spectroscopic data. Thus, the structure of compound **2** was established.

Compound **3** was obtained as a yellowish solid. Its molecular formula C_21_H_20_O_10_ was established by the HRESIMS at *m/z* 433.1133 [M + H]^+^ (calcd for 433.1129), implying 12 degrees of unsaturation. The presence of hydroxyl and carbonyl groups were shown by IR absorption bands at 3310, 1678 cm^−1^, respectively. The ^1^H and ^13^C NMR data of aglycone in **3** (Table 1) were highly similar to those of polyisocoumarin (Jiang et al., 2020), which was previously isolated from *Polygonum cuspidatum.* However, an α-ribose attached at the C-6 of **3** rather than a β-d-glucopyrancose at C-6 in polyisocoumarin. And the NMR data showed that compound **3** and compound **2** have the same ribose at C-6. Thus, the structure of compound **3** was assigned completely.

**Figure 4:**
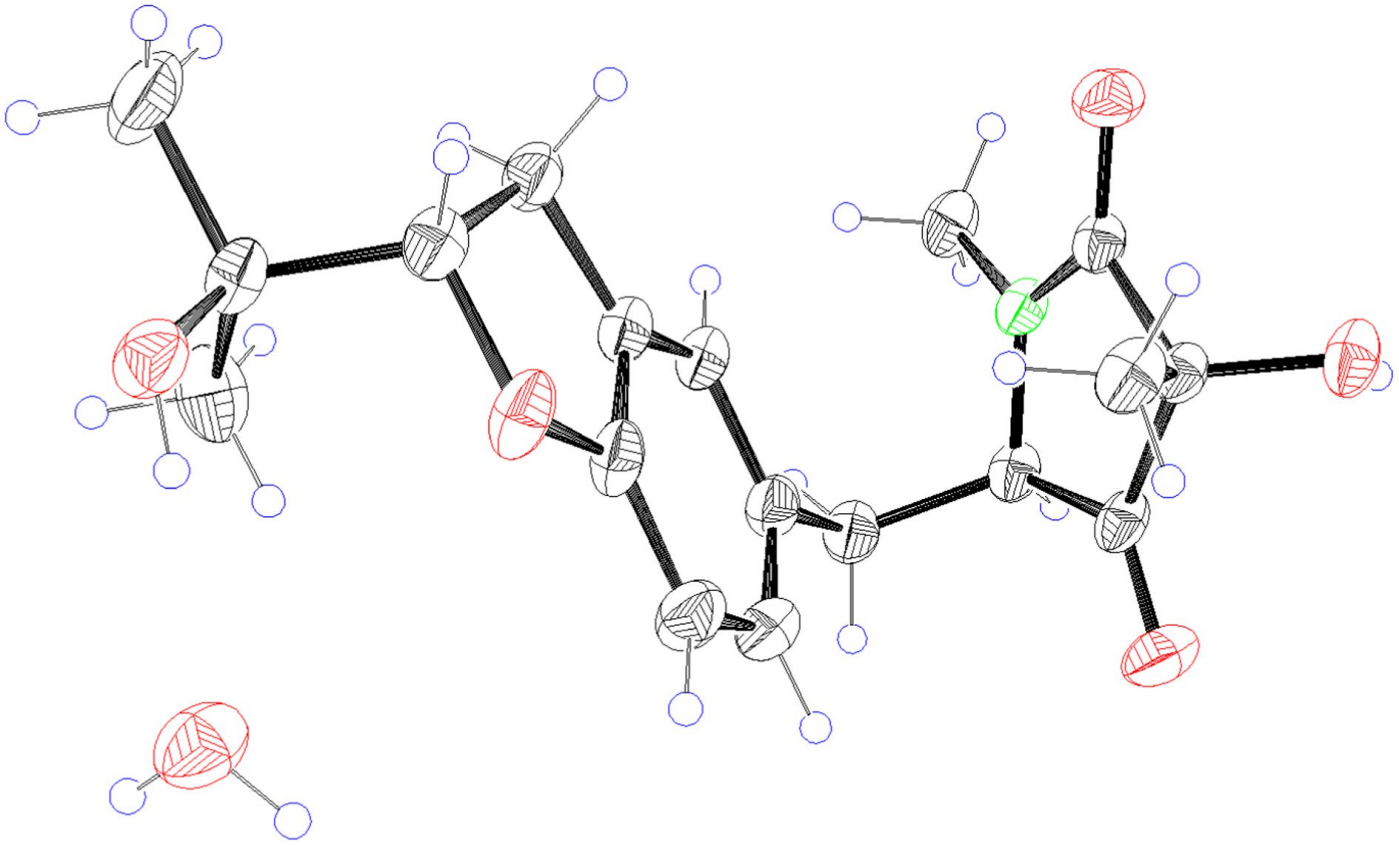
X-ray ORTEP drawing of compound **9**.

**Figure 5:**
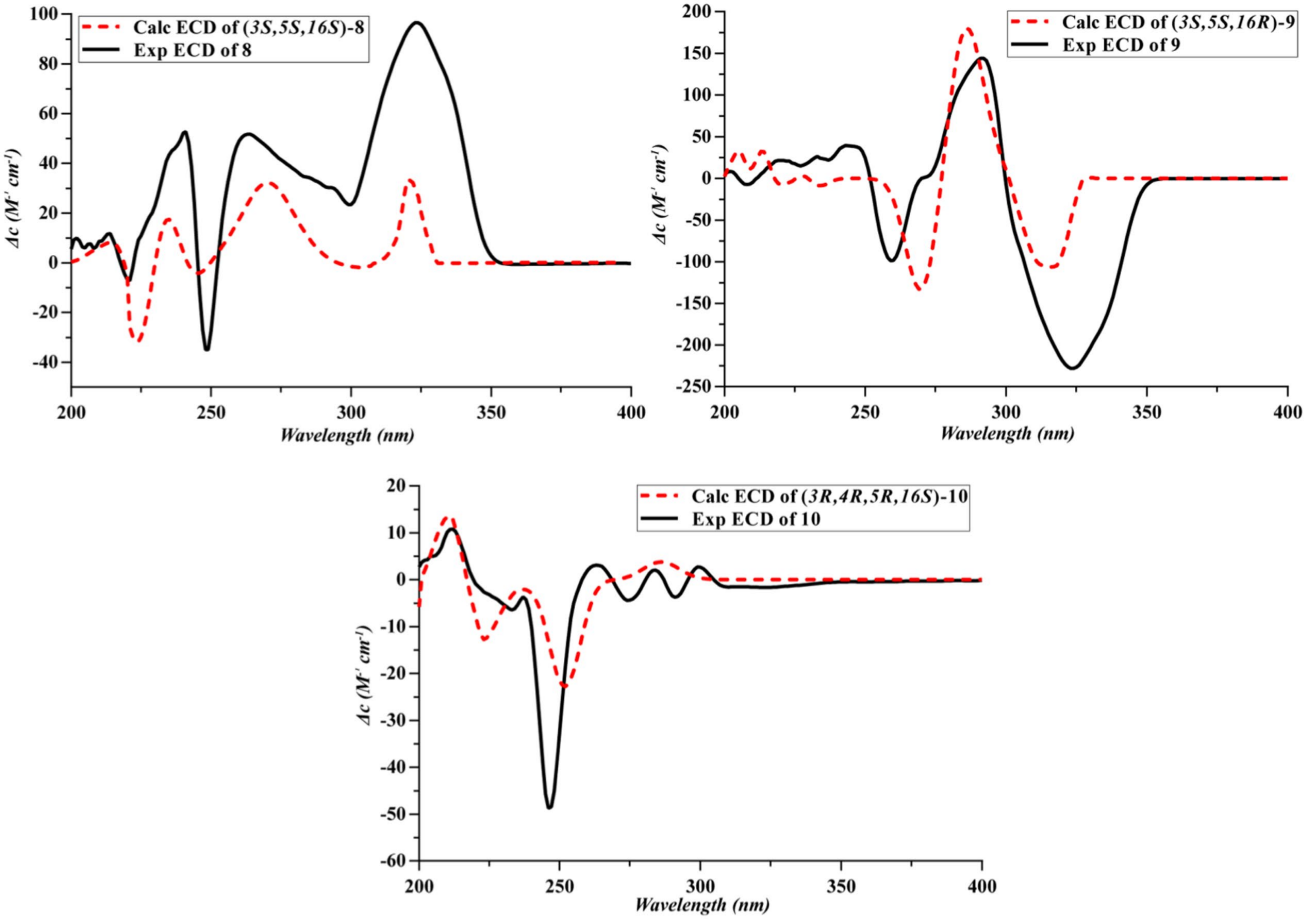
Experimental and calculated ECD spectra of **8**–**10**.

Compounds **8** and **9** were obtained as colorless oil. Their molecular formula C_18_H_23_NO_5_ were established by the HRESIMS at *m/z* 334.1649 and 334.1652 [M + H] ^+^, respectively (calcd for 334.1649), implying eight degrees of unsaturation. The presence of hydroxyl and carbonyl groups were implied by IR absorption bands at 3329 and 1661 cm^−1^, respectively. Detailed analysis of the ^1^H and ^13^C NMR data suggested that **8** possessed the same planar structure as that of rigidiusculamide C (Li et al., 2009). A comparison of the NMR data (Supplementary Table 1) of **8** and **9** suggested that they differed only in the substituent at C-16. Detailed analysis of the HSQC and HMBC spectra allowed the assignment for all proton and carbon resonances of **9**. The relative configuration of **9** was assigned by single-crystal X-ray diffraction as shown in Figure 4. To clarify the absolute configurations of **8** and **9**, ECD calculations were performed by the time dependent density functional theory-predicted curve calculated at the quantum mechanical level. The calculated electronic circular dichroism (ECD) curve of (3*S*, 5*S*, 16*S*)-**8** matched well with the **8** experimental ECD data, and the ECD curve of (3*S*, 5*S*, 16*R*)-**9** is in good agreement with the experimental ECD data of **9**. Therefore, the absolute configuration of compound **8** was determined as 3*S*, 5*S*, 16*S*, and the absolute configuration of compound **9** was 3*S*, 5*S*, 16*R* (Figure 5).

Compound **10** was obtained as a yellow oil. Its molecular formula C_18_H_25_NO_5_ was established by the HRESIMS at *m/z* 336.1808 [M + H]^+^ (calcd for 336.1805), implying seven degrees of unsaturation. The presence of hydroxyl and carbonyl groups were implied by IR absorption bands at 3336 and 1644 cm^−1^, respectively. The NMR data (Supplementary Table 1) of **10** revealed highly similar identical structural features to those found in **8**, except that the C-4 ketone carbon was replaced by an oxygenated methine in **10**. And **10** has the same planar structure as rigidiusculamide D (Li et al., 2009). The NOESY correlation of CH_3_ with H-4 indicates that 3-OH and 4-OH are located on the same face, whereas H-5 and H-4 placed on the opposite face of the ring supported by the absence of a NOESY correlation between H-5 and H-4. To determine the absolute configuration, the simulated electronic circular dichroism (ECD) spectra of **10** were obtained from the calculation of Gaussian 16 based on time-dependent density functional theory. The absolute configurations of C-3, C-4, C-5, and C-16 in **10** were deduced as 3*R*, 4*R*, 5*R*, and 16*S*, respectively, by comparing calculated ECD spectra with the experimental ECD spectrum (Figure 5).

Compound **15** was isolated as a yellow powder. Its molecular formula C_15_H_18_O_4_ was established by the HRESIMS at *m/z* 263.1277 [M + H]^+^ (calcd for 263.1278), implying seven degrees of unsaturation. The presence of hydroxyl and carbonyl groups were implied by IR absorption bands at 3387 and 1698 cm^−1^, respectively. A comparison of NMR data (Supplementary Table 2) with those of nectriacid C (Cui et al., 2016) suggested that **15** possessed a closely similar structure as nectriacid C, except that there is no methoxy group (*δ*_H_ 3.74, *δ_C_* 51.7) in **15**. Its NMR data suggested that **15** belonged to the pentaene diacid derivative. Thus, the structure of compound **15** was assigned completely.

The remaining 13 known compounds from the *F. decemcellulare* F25 were identified as 12-epicitreoisocoumarinol (**4**) (Cui et al., 2016), eoisocoumarinol (**5**) (Cui et al., 2016), (−)-citreoisocoumarin (**6**) (Yamamura et al., 1991; Ola et al., 2013), trichophenol A (**7**) (Liu et al., 2020), rigidiusculamide B (**11**) (Li et al., 2009), fusaristatins A (**12**) (Shiono et al., 2007), enniatin H (**13**) (Nilanonta et al., 2003), enniatin I (**14**) (Nilanonta et al., 2003), nectriacid A (**16**) (Cui et al., 2016), nectriacid B (**17**) (Cui et al., 2016), 4-hydroxy-3,6-dimethyl-2 *H*-pyrane-2-one (**18**) (Hirota et al., 1999), macrocarpon C (**19**), (Ola et al., 2013), and α-linoleic acid (**20**) (Zeng et al., 2017) by comparison of their MS and NMR data with those reported in the literature.

### 3.4. Antifungal assays

Compounds **1**–**20** were assayed for their antifungal activities. The results showed that compounds **13**, **14**, and **17** exhibited inhibitory activities against the plant-pathogenic fungus *C. musae* ACCC31244 with MICs of 256, 64, and 128 μg/mL, respectively. The MIC of the positive control cycliheximide was 32 μg/mL.

### 3.5. Plausible biogenetic pathways

**SCHEME 1:**
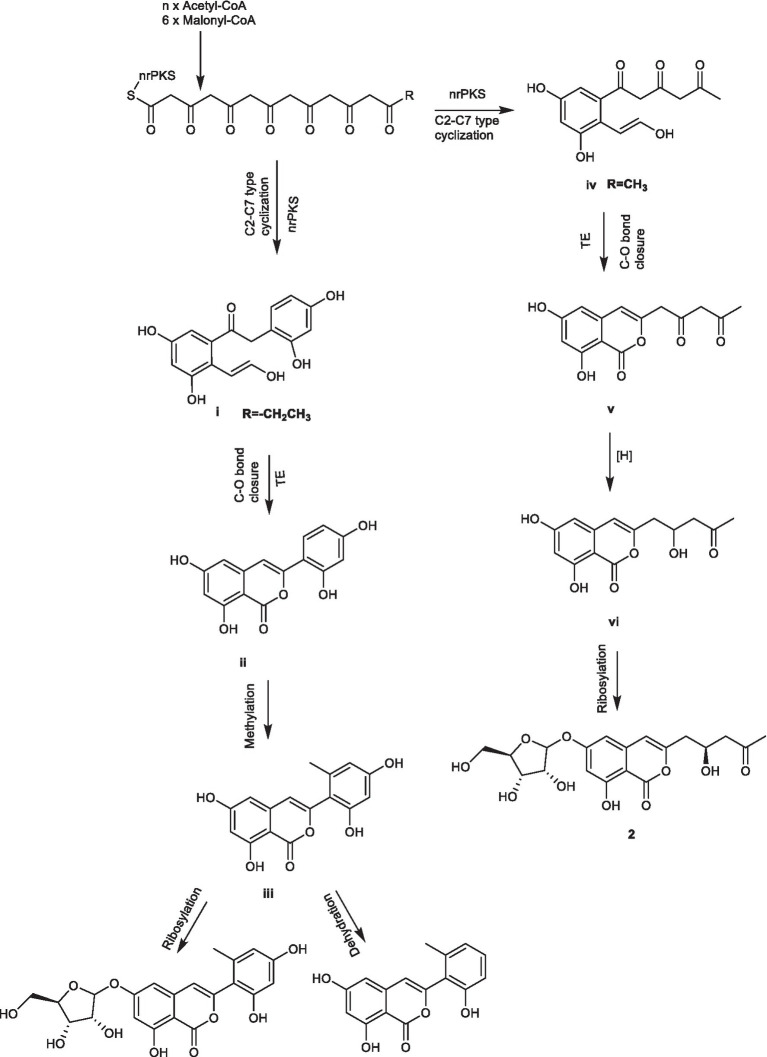
Plausible biogenetic pathways of compounds **1** – **3**.

The biosynthetic pathways of compounds **1** and **3** (Scheme 1) start with condensation catalyzed by nonreducing polyketide synthase (nrPKS) of two acetyl-coenzyme A molecules and six malonyl-CoA molecules resulting in the formation of the intermediate **i** (Liu and Begley, 2020). Intermediate **i** was catalyzed by TE domains or spontaneous C-O bond closure to form intermediate **ii**. The isocoumarin **1** was derived from intermediate **ii** through methylation and dehydration, while, intermediate **ii** will create **3** by methylation and ribosylation. The biosynthetic pathway of **2** (Scheme 1) starts with condensation catalyzed by a modular (nrPKS) of one acetyl-coenzyme A molecule and six malonyl-CoA molecules resulting in the formation of the intermediate **iv**. Intermediate **iv** was catalyzed by thioesterase (TE) domains or spontaneous C − O bond closure to form intermediate **v**. A series of reductive modifications for this intermediate led to intermediate **vi**. Intermediate **vi** then underwent a ribosylation reaction to afford **2**.

**SCHEME 2:**
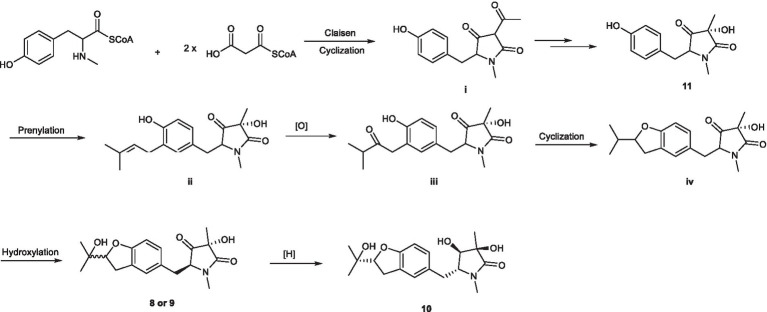
Plausible biogenetic pathways of compounds **8** – **10**.

As shown in Scheme 2, pyrrolidones originate from the cyclization of an amino acid and a polyketide (Royles, 1995; Li et al., 2009), leading to the formation of **11**. The pyrrolidones **8** and **9** were likely to be biogenetically derived from **11** through prenylation, oxidation, cyclization, and hydroxylation. The derivative **10** was formed from **8** or **9** through one-step hydrogenation.

## 4. Conclusion

In summary, the chemical investigation on the endophytic fungus *F. decemcellulare* F25 resulted in the isolation and identification of twenty secondary metabolites, including three new isocoumarin derivatives (**1–3**), three new pyrrolidinones (**8–10**), and one new pentaene diacid (**15**), together with thirteen known compounds. Compounds **13**, **14**, and **17** exhibited antifungal activities against plant pathogen *C. musae* ACCC31244. This study reveals the potential of endophytic fungi as a promising source of bioactive compounds.

## Data availability statement

The datasets presented in this study can be found in online repositories. The names of the repository/repositories and accession number(s) can be found at: GenBank No. OQ001346 of ITS region of the rDNA in NCBI and Deposition Number 2232225 of X-ray crystallographic data in CCDC.

## Author contributions

ZS and KZ: conception or design. KZ, ZS, YJS, GL, SX, and CY: acquisition, analysis, or interpretation of data. ZS, KZ, GL, and CY: drafting the work or revising and final approval of the manuscript. All authors have reviewed the manuscript. All authors contributed to the article and approved the submitted version.

## Funding

We gratefully acknowledge the National Natural Science Foundation of China (grant nos. 22067002 and 82060635), the Science and Technology Foundation of Guizhou (grant no. J[2020]1Y049), and Guizhou University (grant no. (2018)04) for the financial supports.

## Conflict of interest

The authors declare that the research was conducted in the absence of any commercial or financial relationships that could be construed as a potential conflict of interest.

## Publisher’s note

All claims expressed in this article are solely those of the authors and do not necessarily represent those of their affiliated organizations, or those of the publisher, the editors and the reviewers. Any product that may be evaluated in this article, or claim that may be made by its manufacturer, is not guaranteed or endorsed by the publisher.
