# Supplementary material for: Secondary metabolites from the Endophytic fungi Fusarium decemcellulare F25 and their antifungal activities
Source: Front Microbiol. 2023 Feb 1;14:1127971. doi: 10.3389/fmicb.2023.1127971 (PMC9929939; doi:10.3389/fmicb.2023.1127971)

checkCIF/PLATON report

Structure factors have been supplied for datablock(s) mo_20221130wy_autored

THIS REPORT IS FOR GUIDANCE ONLY. IF USED AS PART OF A REVIEW PROCEDURE FOR PUBLICATION, IT SHOULD NOT REPLACE THE EXPERTISE OF AN EXPERIENCED CRYSTALLOGRAPHIC REFEREE.

No syntax errors found.   CIF dictionary   Interpreting this report

Datablock: mo_20221130wy_autored

Bond precision: C-C = 0.0071 A   Wavelength=0.71073

Cell:	a=6.4597(7)	b=7.5258(7)	c=9.4129(7)
	alpha=95.900(7)	beta=100.927(8)	gamma=93.981(8)
Temperature:	297 K		

	Calculated	Reported
Volume	445.07(7)	445.07(7)
Space group	P 1	P 1
Hall group	P 1	P 1
Moiety formula	C18 H23 N O5, H2 O	C18 H23 N O5, H2 O
Sum formula	C18 H25 N O6	C18 H25 N O6
Mr	351.39	351.39
Dx,g cm-3	1.311	1.311
Z	1	1
Mu (mm-1)	0.098	0.098
F000	188.0	188.0
F000’	188.10	
h,k,lmax	7,8,11	7,8,11
Nref	3132[1566]	2823
Tmin,Tmax		0.293,1.000
Tmin’		

Correction method= # Reported T Limits: Tmin=0.293 Tmax=1.000
AbsCorr = MULTI-SCAN

Data completeness= 1.80/0.90   Theta(max)= 25.006

R(reflections)= 0.0596(2547)   wR2(reflections)= 0.1706(2823)

S = 1.075   Npar= 236

The following ALERTS were generated. Each ALERT has the format
test-name_ALERT_alert-type_alert-level.
Click on the hyperlinks for more details of the test.

Alert level C

```
STRVA01_ALERT_2_C           Chirality of atom sites is inverted?
           From the CIF: _refine_ls_abs_structure_Flack    1.600
           From the CIF: _refine_ls_abs_structure_Flack_su    1.000
PLAT053_ALERT_1_C Minimum Crystal Dimension Missing (or Error) ...     Please Check
PLAT054_ALERT_1_C Medium  Crystal Dimension Missing (or Error) ...     Please Check
PLAT055_ALERT_1_C Maximum Crystal Dimension Missing (or Error) ...     Please Check
PLAT089_ALERT_3_C Poor Data / Parameter Ratio (Zmax < 18) ........       6.56 Note
PLAT340_ALERT_3_C Low Bond Precision on  C-C Bonds ...............    0.00712 Ang.
PLAT907_ALERT_2_C Flack x > 0.5, Structure Needs to be Inverted? .       1.60 Check
PLAT911_ALERT_3_C Missing FCF Refl Between Thmin & STh/L=    0.595         18 Report
PLAT913_ALERT_3_C Missing # of Very Strong Reflections in FCF ....          7 Note
```

Alert level G

```
PLAT007_ALERT_5_G Number of Unrefined Donor-H Atoms ..............          4 Report
PLAT032_ALERT_4_G Std. Uncertainty on Flack Parameter Value High .      1.000 Report
PLAT072_ALERT_2_G SHELXL First  Parameter in WGHT  Unusually Large       0.12 Report
PLAT398_ALERT_2_G Deviating  C-O-C   Angle From 120 for O2       .      107.7 Degree
PLAT791_ALERT_4_G Model has Chirality at C2          (Sohnke SpGr)          S Verify
PLAT791_ALERT_4_G Model has Chirality at C5          (Sohnke SpGr)          R Verify
PLAT791_ALERT_4_G Model has Chirality at C8          (Sohnke SpGr)          R Verify
PLAT909_ALERT_3_G Percentage of I>2sig(I) Data at Theta(Max) Still        77% Note
PLAT916_ALERT_2_G Hooft y and Flack x Parameter Values Differ by .       0.10 Check
PLAT933_ALERT_2_G Number of HKL-OMIT Records in Embedded .res File          4 Note
PLAT978_ALERT_2_G Number C-C Bonds with Positive Residual Density.          0 Info
```

---

0 **ALERT level A** = Most likely a serious problem - resolve or explain
0 **ALERT level B** = A potentially serious problem, consider carefully
9 **ALERT level C** = Check. Ensure it is not caused by an omission or oversight
11 **ALERT level G** = General information/check it is not something unexpected

3 ALERT type 1 CIF construction/syntax error, inconsistent or missing data
7 ALERT type 2 Indicator that the structure model may be wrong or deficient
5 ALERT type 3 Indicator that the structure quality may be low
4 ALERT type 4 Improvement, methodology, query or suggestion
1 ALERT type 5 Informative message, check

It is advisable to attempt to resolve as many as possible of the alerts in all categories. Often the minor alerts point to easily fixed oversights, errors and omissions in your CIF or refinement strategy, so attention to these fine details can be worthwhile. In order to resolve some of the more serious problems it may be necessary to carry out additional measurements or structure refinements. However, the purpose of your study may justify the reported deviations and the more serious of these should normally be commented upon in the discussion or experimental section of a paper or in the "special_details" fields of the CIF. checkCIF was carefully designed to identify outliers and unusual parameters, but every test has its limitations and alerts that are not important in a particular case may appear. Conversely, the absence of alerts does not guarantee there are no aspects of the results needing attention. It is up to the individual to critically assess their own results and, if necessary, seek expert advice.

Publication of your CIF in IUCr journals

A basic structural check has been run on your CIF. These basic checks will be run on all CIFs submitted for publication in IUCr journals (*Acta Crystallographica*, *Journal of Applied Crystallography*, *Journal of Synchrotron Radiation*); however, if you intend to submit to *Acta Crystallographica Section C* or *E* or *IUCrData*, you should make sure that full publication checks are run on the final version of your CIF prior to submission.

Publication of your CIF in other journals

Please refer to the *Notes for Authors* of the relevant journal for any special instructions relating to CIF submission.

PLATON version of 28/11/2022; check.def file version of 28/11/2022

Datablock mo_20221130wy_autored - ellipsoid plot

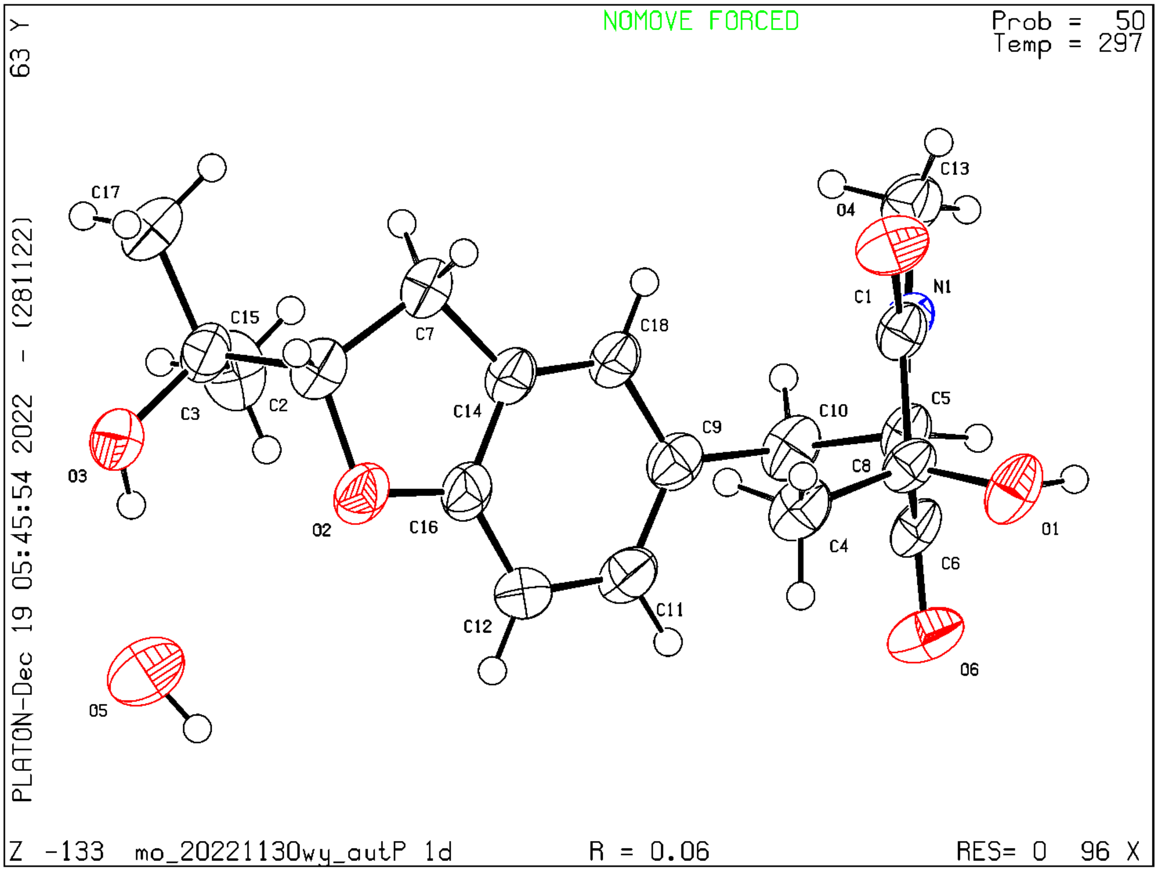

Supplement: Supplementary file 1 [file Data_Sheet_1.PDF]
