# Supplementary material for: Secondary metabolites from the Endophytic fungi Fusarium decemcellulare F25 and their antifungal activities
Source: Front Microbiol. 2023 Feb 1;14:1127971. doi: 10.3389/fmicb.2023.1127971 (PMC9929939; doi:10.3389/fmicb.2023.1127971)
Supplement: Supplementary file 2 [file Data_Sheet_2.PDF]

Supplementary Material

Secondary Metabolites from the Endophytic Fungi *Fusarium decemcellulare* F25 and Their Antifungal Activities

Ziwei Song [1,2], Yan-Jun Sun[3], Shuangyu Xu[4], Gang Li[3*], Chunmao Yuan[4*], Kang Zhou[1,2*]

[1]School of Pharmaceutical Sciences, Guizhou University, Guiyang 550025, China

[2]Key laboratory of Plant Resource Conservation and Germplasm Innovation in Mountainous Region, Ministry of Education, Guizhou University, Guiyang 550025, China

[3]Department of Natural Medicinal Chemistry and Pharmacognosy, School of Pharmacy, Qingdao University, Qingdao 266071, China

[4]State Key Laboratory of Functions and Applications of Medicinal Plants, Guizhou Medical University, Guiyang 550014, China

*** Correspondence:**
Gang Li
gang.li@qdu.edu.cn
Chunmao Yuan
yuanchunmao01@126.com
Kang Zhou
kangzhouzj@126.com

Keywords: *Fusarium decemcellulare* F25, secondary metabolites, isocoumarins, pyrrolidinones, antifungal activities.

Contents

1 Supplementary Data

The strain's (*Fusarium decemcellulare* F25) ITS sequence of the rDNA

The physicochemical data of the known compounds

2 Supplementary Tables

Tables 1−3:^{1}H NMR and ^{13}C NMR Data of **8**, **9**, **13**, **14**, and **15**

3 Supplementary Figures

Supplementary Figure 1.The HPLC analyses of crude extracts of *Fusarium decemcellulare* F25 in different cultural conditions

Supplementary Figure 2. The antifungal activities of crude extracts were evaluated against *Colletotrichum musae* ACCC31244 (Positive control: cycloheximide; Negative control: methanol)

Supplementary Figures 3−10. NMR, MS, UV, and IR spectra of compound **1**

Supplementary Figures 11−19. NMR, MS, UV, and IR spectra of compound **2**

Supplementary Figures 20−28. NMR, MS, UV, and IR spectra of compound **3**

Supplementary Figures 29−33. NMR, MS, UV, and IR spectra of compound **8**

Supplementary Figures 34−42. NMR, MS, UV, and IR spectra of compound **9**

Supplementary Figures 43−51. NMR, MS, UV, and IR spectra of compound **10**

Supplementary Figures 52−56. NMR, MS, UV, and IR spectra of compound **15**

1. Supplementary Data

The strain's (*Fusarium decemcellulare* F25) ITS sequence of the rDNA

TGATCGAGGTCACATTCAGAAGTTGGGGGTTTAACGGCTTGGCCGCGCCGCGTTCCAGT
TGCGAGGTGTTAGCTACTACGCAATGGAGGCTACAGCGAGACCGCCACTAGATTTGGGG
GACGGCGACTATCGCCGATCCCCAACACCAAGCCCTAGGGGCTTGAGGGTTGAAATGAC
GCTCGAACAGGCATGCCCGCCAGAATACTGGCGGGCGCAATGTGCGTTCAAAGATTCGA
TGATTCACTGAATTCTGCAATTCACATTACTTATCGCATTTCGCTGCGTTCTTCATCGATG
CCAGAACCAAGAGATCCGTTGTTGAAAGTTTTGATTTATTTGTTTTGTTTTACTCAGAAG
ATCCACAAGAATACATAGAGTTTGGGGTTCCTCTGGCAGCGAGCGGCGCCCGATCTCTC
GGAGCACCGTCGTTGAGTCTGCCGAGGCAAATTATAGGTATGTTCACAGGGGTTTGGGA
GTTGTAAACTCGGTAATGATCCCTCCGCAGGCCCCCCCTTCCGGAAGGGA

The physicochemical data of the known compounds

12-epicitreoisocoumarinol (**4**) (Cui et al., 2016): white powder; ^{1}H NMR (400 MHz, methanol-d_4) δ 6.38 (1H, s, H-4), 6.32 (2H, s, H-5, H-7), 2.58 (1H, dd, J =14.5, 8.0 Hz, H-9a), 2.66 (1H, dd, J =14.5, 5.0 Hz, H-9b), 4.22 (1H, m, H-10), 1.58 (2H, m, H-11), 4.02 (1H, m, H-12), 1.20 (3H, d, J =6.2 Hz, 13-H); ^{13}C NMR (100 MHz, methanol-d_4) δ 167.9 (C-1), 156.2 (C-3), 107.1 (C-4), 141.3 (C-4a), 103.7 (C-5), 167.4 (C-6), 102.6 (C-7), 164.8 (C-8), 99.8 (C-8a), 43.3 (C-9), 67.1 (C-10), 46.6 (C-11), 65.2 (C-12), 24.3 (C-13).

Eoisocoumarinol (**5**) (Cui et al., 2016): white powder; ^{1}H NMR (400 MHz, methanol-d_4) δ 6.38 (1H, s, H-4), 6.31 (2H, s, H-5, H-7), 2.57 (1H, dd, J = 14.5, 8.3 Hz, H-9a), 2.69 (1H, dd, J = 14.5, 4.5 Hz, H-9a), 4.14 (1H, m, H-10), 1.71 (1H, m, H-11a), 1.61 (1H, m, H-11b), 4.00 (1H, m, H-12), 1.20 (3H, d, J =6.1, 13-H); ^{13}C NMR (100 MHz, methanol-d_4) δ 167.9 (C-1), 156.0 (C-3), 107.3 (C-4), 141.2 (C-4a), 103.7 (C-5), 167.3 (C-6), 102.6 (C-7), 164.8 (C-8), 99.8 (C-8a), 42.5 (C-9), 68.7 (C-10), 46.4 (C-11), 67.1 (C-12), 23.6 (C-13).

(−)-Citreoisocoumarin (**6**) (Yamamura et al., 1991; Ola et al., 2013): yellow oil; ^{1}H NMR (400 MHz, DMSO-d_6) δ 6.47 (1H, s, H-4), 6.36 (1H, d, J =2.1 Hz, H-5), 6.31 (1H, d, J =2.1 Hz, H-7), 2.58 (2H, m, H-9), 4.27 (1H, m, H-10), 2.58 (2H, m, H-11), 2.10 (3H, s, H-13); ^{13}C NMR (100 MHz, DMSO-d_6) δ 165.6 (C-1), 154.7 (C-3), 105.7 (C-4), 139.6 (C-4a), 102.6 (C-5), 165.6 (C-6), 101.5 (C-7), 162.7 (C-8), 98.3 (C-8a), 40.9 (C-9), 64.6 (C-10), 50.3 (C-11), 207.2 (C-12), 30.5 (C-13).

Trichophenol A (**7**) (Liu et al., 2020): white powder; ^{1}H NMR (600 MHz, DMSO-d_6) δ 6.59 (1H, s, H-4), 6.45 (1H, d, J =2.2 Hz, H-5), 6.35 (1H, d, J =2.2 Hz, H-7), 6.24 (1H, d, J =2.2 Hz, H-11), 6.18 (1H, J =2.2 Hz, H-13), 2.11 (3H, s, H-15); ^{13}C NMR (150 MHz, DMSO-d_6) δ 166.3 (C-1), 151.4 (C-3), 108.8 (C-4), 139.9 (C-4a), 103.1 (C-5), 165.7 (C-6), 101.8 (C-7), 162.7 (C-8), 98.3 (C-8a), 111.6 (C-9), 157.2 (C-10), 100.2 (C-11), 159.2 (C-12), 108.3 (C-13), 139.2 (C-14), 19.8 (C-15).

Rigidiusculamide B (**11**) (Li et al., 2009): colorless oil; ^{1}H NMR (400 MHz, DMSO-d_6) δ 4.21 (1H, t, J = 4.3 Hz, H-5), 3.02 (1H, dd, J =14.5, 4.3 Hz, H-6a), 2.89 (1H, dd, J=14.5, 4.3 Hz, H-6b), 6.80 (2H, d, J = 8.5 Hz, H-8, H-12), 6.64 (2H, d, J = 8.5 Hz, H-9, H-11), 2.96 (3H, s, 13-H), 0.34 (3H, s, 14-H); ^{13}C NMR (100 MHz, DMSO-d_6) δ 172.6 (C-2), 70.0 (C-3), 210.9 (C-4), 66.8 (C-5), 33.1 (C-6), 125.0 (C-7), 131.0 (C-8), 115.2 (C-9), 156.4 (C-10), 115.2 (C-11), 131.0 (C-12), 27.7 (C-13), 19.0 (C-14).

Fusaristatins A (**12**) (Shiono et al., 2007) : colorless oil; ^{1}H NMR (400 MHz, DMSO-d_6) δ 0.83~0.85 (7H, m, H-1, H-6a, H-7′), 0.97~1.00 (3H, m, H-2, H-6b), 1.05~1.07 (7H, m, H-3, H-4, H-5, H-8), 1.18~1.31 (12H, m, H-7, H-15′, H-16, H-17, H-19′), 1.34~1.41 (2H, m, H-16, H-17), 1.52~1.62 (2H, m, H-9), 1.76 (3H, s, H-11′), 1.83~1.99 (2H, m, H-31), 2.03~2.08 (2H, m, H-32), 2.18 (2H, m, H-19, H-26), 2.44 (1H, m, H-15), 2.67 (1H, m, H-25), 2.80 (1H, m, H-25), 4.23 (1H, m, H-30), 4.88 (1H, m, H-18), 5.28 (1H, s, H-22′a), 5.78 (1H, s, H-22′b), 6.05 (1H, t, J = 7.5 Hz, 10-H), 6.18 (1H, d, J = 15.7 Hz, 13-H), 6.63 (1H, t, J = 5.8 Hz, 24-NH), 6.57 (1H, s, 34-NH_a), 7.20 (1H, d, J = 17.5 Hz, 12-H), 7.26 (1H, s, 34-NH_b), 8.02 (1H, d, J = 8.0 Hz, 28-NH), 9.27 (1H, s, 21-NH); ^{13}C NMR (100 MHz, DMSO-d_6) δ 14.0 (C-1), 22.1 (C-2), 31.3 (C-3), 26.0 (C-4), 28.3 (C-5), 36.3 (C-6), 31.8 (C-7), 19.7 (C-7′), 35.7 (C-8), 25.8 (C-9), 143.6 (C-10), 132.8 (C-11), 12.0 (C-11′), 147.2 (C-12), 122.8 (C-13), 202.9 (C-14), 42.8 (C-15), 16.5 (C-15′), 27.0 (C-16), 29.0 (C-17), 75.5 (C-18), 42.8 (C-19), 14.6 (C-19′), 172.5 (C-20), 22 (C-115.7), 163.3 (C-23), 41.4 (C-25), 41.0 (C-26), 14.5 (C-26′), 173.2 (C-27), 51.5 (C-29), 170.7 (C-30), 26.3 (C-31), 31.3 (C-32), 173.6 (C-33).

Nectriacid A (**16**) (Cui et al., 2016): yellow powder; ^{1}H NMR (400 MHz, DMSO-d_6) δ 5.73 (1H, s, H-2), 6.21 (1H, s, H-4),), 6.58 (1H, d, J = 15.0, H-6), 6.78 (1H, dd, J = 15.0, 11.2 Hz, H-7), 6.58 (1H, d, J = 15.0 Hz, H-8), 6.61 (1H, d, J = 11.2 Hz, H-9), 7.27 (1H, d, J = 15.5 Hz, H-10), 5.86 (1H, d, J = 15.5 Hz, H-11), 2.24 (3H, s, 13-H), 2.02 (3H, s, 14-H), 1.93 (3H, s, 15-H); ^{13}C NMR (100 MHz, DMSO-d_6) δ 167.8 (C-1), 120.2 (C-2), 151.6 (C-3), 136.1 (C-4), 138.6 (C-5), 141.8 (C-6), 126.3 (C-7), 138.4 (C-8), 134.3 (C-9), 148.1 (C-10), 117.9 (C-11), 167.5 (C-12), 18.8 (C-13), 14.2 (C-14), 12.5 (C-15).

Nectriacid B (**17**) (Cui et al., 2016): yellow powder; ^{1}H NMR (400 MHz, DMSO-d_6) δ 5.73 (1H, s, H-2), 6.22 (1H, s, H-4), 6.59 (1H, d, J = 14.9, H-6), 6.78 (1H, dd, J = 14.9, 11.3 Hz, H-7), 6.67 (1H, d, J = 11.3, H-8), 7.34 (1H, d, J = 15.6 Hz, H-10), 5.95 (1H, d, J = 15.6 Hz, H-11), 2.24 (3H, d, J = 1.3 Hz, 13-H), 2.03 (3H, d, J = 1.3 Hz, 14-CH_3), 1.94 (3H, s, 15-H), 3.68 (3H, s, -OCH_3); ^{13}C NMR (100 MHz, DMSO-d_6) δ 167.5 (C-1), 120.3 (C-2), 151.7 (C-3), 138.6 (C-4), 134.2 (C-5), 142.2 (C-6), 126.2 (C-7), 139.2 (C-8), 136.3 (C-9), 148.7 (C-10), 116.4 (C-11), 166.9 (C-12), 18.8 (C-13), 14.2 (C-14), 12.5 (C-15), 51.3 (-OCH_3).

4-hydroxy-3,6-dimethyl-2 *H*-pyrane-2-one (**18**) (Hirota et al., 1999): colorless oil; ^{1}H NMR (400 MHz, DMSO-d_6) δ 5.98 (1H, s, H-5), 2.13 (3H, s, H-7), 1.73 (3H, s, H-8); ^{13}C NMR (100 MHz, DMSO-d_6) δ 165.2 (C-2), 165.1 (C-3), 159.3 (C-4), 99.9 (C-5), 96.2 (C-6), 19.2 (C-7), 8.3 (C-8).

Macrocarpon C (**19**) (Ola et al., 2013): white powder; ^{1}H NMR (400 MHz, DMSO-d_6) δ 6.04 (1H, d, J = 2.3 Hz, H-3), 6.07 (1H, d, J = 2.3 Hz, H-5), 3.65 (1H, s, H-7), 6.12 (1H, d, J = 2.1 Hz, H-9), 6.10 (1H, t, J = 2.1, H-11), 6.12 (1H, d, J = 2.1 Hz, H-13), 2.20 (3H, s, 14-H); ^{13}C NMR (100 MHz, DMSO-d_6) δ 165.9 (C-2), 113.3 (C-3), 178.8 (C-4), 113.3 (C-5), 167.7 (C-6), 40.1 (C-7), 137.6 (C-8), 107.0 (C-9), 158.6 (C-10), 101.3 (C-11), 158.6 (C-12), 107.0 (C-13), 19.2 (C-14).

α-linoleic acid (**20**) (Zeng et al., 2017): white oil; ^{1}H NMR (400 MHz, DMSO-d_6) δ 0.85 (3H, m, H-18), 1.23~1.32 (14H, m, H-4, H-5, H-6, H-7, H-15, H-16, H-17), 1.48 (2H, m, H-3), 1.98~2.03 (4H, m, H-8, H-14), 2.16 (2H, t, J = 7.4 Hz, H-2), 2.72 (2H, t, J = 6.4 Hz, H-11), 5.25~5.36 (4H, m, H-9, H-10, H-12, H-13); ^{13}C NMR (100 MHz, DMSO-d_6) δ 174.3 (C-1), 33.7 (C-2), 24.5 (C-3), 28.7 (C-4), 28.6 (C-5), 28.7 (C-6), 29.1 (C-7), 26.7 (C-8), 129.6 (C-9), 127.7 (C-10), 25.2 (C-11), 127.7 (C-12), 130.0 (C-13), 26.7 (C-14), 28.81 (C-15), 31.0 (C-16), 22.0 (C-17),13.8 (C-18).

Cui, H., Liu, Y., Nie, Y., Liu, Z., Chen, S., Zhang, Z., et al. (2016). Polyketides from the mangrove-derived endophytic fungus *Nectria* sp. HN001 and their α-glucosidase inhibitory activity. *Mar. Drugs.* 14, 1660-3397.

Yamamura, S., Lai, S., Shizuri, Y., Kawai, K., and Furukawa, H. (1991). Three new phenolic metalolites from *Penicillium* species. *Heterocycles.* 32, 297-305.

Ola, A. R. B., Thomy, D., Lai, D., Brötz-Oesterhelt, H., and Proksch, P. (2013). Inducing secondary metabolite production by the endophytic fungus *Fusarium tricinctum* through coculture with *Bacillus* subtilis. *J. Nat. Prod.* 76, 2094-2099.

Liu, X. H., Hou, X. L., Song, Y. P., Wang, B. G., and Ji, N. Y. (2020). Cyclonerane sesquiterpenes and an isocoumarin derivative from the marine-alga-endophytic fungus *Trichoderma citrinoviride* A-WH-20-3. *Fitoterapia* 141, 104469.

Li, J., Liu, S., Niu, S., Zhuang, W., and Che, Y. (2009). Pyrrolidinones from the ascomycete fungus *Albonectria rigidiuscula*. *J. Nat. Prod.* 72, 2184-2187.

Shiono, Y., Tsuchinari, M., Shimanuki, K., Miyajima, T., Murayama, T., Koseki, T., et al. (2007). Fusaristatins A and B, two new cyclic lipopeptides from an endophytic *Fusarium* sp. *J. Antibiot.* 60, 309-316.

Hirota, A., Nemoto, A., Tsuchiya, Y., Hojo, H., and Abe, N. (1999). Isolation of a 2-pyrone compound as an antioxidant from a fungus and its new reaction product with 1, 1-diphenyl-2-picrylhydrazyl radical. *Biosci. Biotechnol. Biochem.* 63, 418-420.

Zeng, J. X., Bing, X. B., Ying, B. I., Wang, J., Ren, G., Wang, H. L., et al. (2017). Chemical constitutes from plantaginis semen(Ⅱ). *Chin. J. Exp. Tradit. Med. Form.* 23, 81-84.

2 Supplementary Tables and Figures

2.1 Supplementary Tables

Supplementary Table 1. ^{1}H NMR (400MHz, δ in ppm) and ^{13}C NMR Data (100MHz, δ in ppm) of **8**(Acetone-d_6), **9** (Acetone-d_6), and **10** (Methanol-d_4).

Position	8		9		10	
	δ_C, type	δ_H (J in Hz)	δ_C, type	δ_H (J in Hz)	δ_C, type	δ_H (J in Hz)
2	173.5, C		173.8, C		177.3, C	
3	71.2, C		71.2, C		77.8, C	
4	210.7, C		210.8, C		75.1, CH	3.83, d (5.1)
5	68.1, CH	4.2, t (4.4)	68.2, CH	4.23, t (4.3)	64.7, CH	3.91, m
6a	34,6, CH_2	3.16, m	34.4, CH_2	3.13, m	33.7, CH_2	2.83, dd (13.5, 6.1)
6b						3.01, dd (13.5, 8.5)
7	127.8, C		127.7, C		131.2, C	
8	127.3, CH	6.77, d (8.3)	130.2, CH	6.76, d (8.1)	129.8, CH	7.02, d (8.1)
9	109.3, CH	6.59, d (8.3)	109.3, CH	6.58, d (8.1)	109.7, CH	6.66, d (8.1)
10	160.3, C		160.2, C		160.0, C	
11	128.9, C		128.8, C		128.8, C	
12	130.3, CH	6.87, s	127.4, CH	6.85, s	126.9, CH	7.14, s
13	28.2, CH_3	3.03, s	28.3, CH_3	3.03, s	29.0, CH_3	2.72, s
14	20.0, CH_3	0.53, s	19.8, CH_3	0.51, s	18.9, CH_3	1.32, s
15a	30.9, CH_2	3.10, m	30.8, CH_2	3.13, m	31.5, CH_2	3.14, dd (9.0, 5.4)
15b						
16	90.2, CH	4.56, dd (9.6, 8.0)	90.1, CH	4.56, t (8.4)	90.4, CH	4.56, t (9.0)
17	71.5, C		71.5, C		72.5, C	
18	25.2, CH_3	1.18, s	24.9, CH_3	1.16, d (2.1)	25.2, CH_3	1.21, s
19	26.1, CH_3	1.18, s	26.0, CH_3	1.16, d (2.1)	25.4, CH_3	1.24, s

Supplementary Table 2. ^{1}H NMR (400 MHz, δ in ppm) and ^{13}C NMR Data (100 MHz, δ in ppm) of **15** (DMSO-d_6).

Position	**15**	
	δ_C, type	δ_H (J in Hz)
1	167.7, C	
2	120.2, CH	5.62, s
3	151.6, C	
4	134.2, CH	6.08, s
5	136.8, C	
6	134.2, CH	6.94, d (14.8)
7	127.8, CH	6.80, dd (14.8, 11.2)
8	138.6, CH	6.68, d (11.2)
9	134.8, C	
10	148.0 CH	7.27, d (15.6)
11	118.1, CH	5.87, d (15.6)
12	167.3, C	
13	19.0, CH_3	2.20, s
14	20.9, CH_3	1.99, s
15	12.5, CH_3	1.93, s

Supplementary Table 3. ^{1}H NMR (400MHz, δ in ppm) and ^{13}C NMR Data (100MHz, δ in ppm) of **13** and **14** (DMSO-d_6)

Position	**13**		**14**	
	δ_C, type	δ_H (J in Hz)	δ_C, type	δ_H (J in Hz)
NMeVal	3 units		3 units	
1	170.0, 170.0, 169.9		170.5, 170.4, 170.4	
2	61.1, 61.1, 60.9	4.64, (3H, m)	61.6, 61.4, 61.4	4.70-4.64, (3H, m)
3	27.6, 27.5, 27.4	2.06-2.21, (3H, m)	28.0, 27.9, 27.8	2.21-2.07, (3H, m)
4	19.8, 19.7, 19.7	1.00-0.82, (9H, m)	20.2, 20.2, 20.1	1.00-0.81 ,(9H, m)
4'	19.4, 19.3 ,19.1	1.00-0.82, (9H, m)	19.8, 19.7, 19.6	1.00-0.81, (9H, m)
N-CH_3	31.6, 31.4, 31.4	3.06, (3H, s)	31.9, 31.9, 31.7	3.05, (3H, s)
		3.05, (3H, s)		3.03, (3H, s)
		3.04, (3H, s)		3.02, (3H, s)
Hiv	2 units		1unit	
1	168.6, 168.6		168.9	
2	74.3, 74.1	5.23, (1H, d, 8.0); 5.19, (1H, d, 8.0)	74.7	5.22, (1H, d, 8.1)
3	29.6, 29.6	2.06-2.21, (2H, m)	30.1	2.21-2.07, (1H, m)
4	18.1, 18.1	1.00-0.82, (6H, m)	18.6	1.00-0.81, (3H, m)
4'	17.9, 17.8	1.00-0.82, (6H, m)	18.3	1.00-0.81, (3H, m)
Hmp	1 units		2 units	
1	168.8		169.1, 169.1	
2	72.8	5.31,(1H, d, 6.4)	73.4, 73.3	5.34, (2H, d, 6.7)
3	35.8	1.86, (1H, m)	36.3, 36.2	1.91-1.84, (2H, m)
4	24.7	1.38, (1H, m)	25.1, 25.1	1.40-1.33, (2H, m)
		1.17, (1H, m)		1.20-1.31, (2H, m)
5	11.3	1.00-0.82, (3H, m)	11.8, 11.8	1.00-0.81, (6H, m)
3-CH_3	14.2	1.00-0.82, (3H, m)	14.7, 14.7	1.00-0.81, (6H, m)

2.2 Supplementary Figures

A.

CDA culture

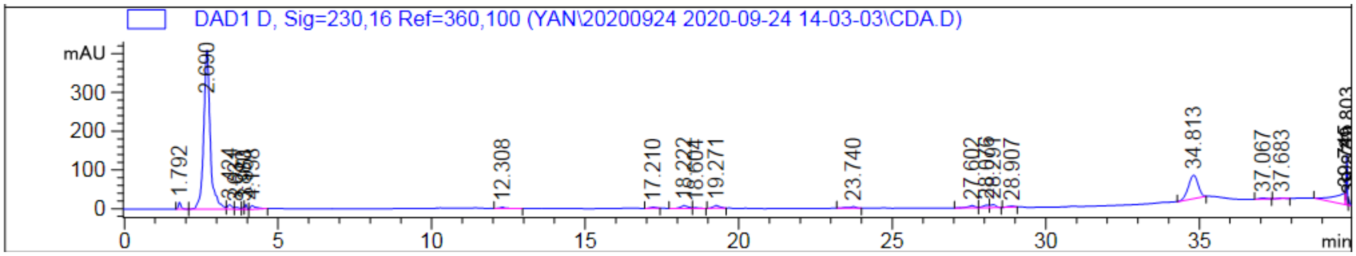

F25-CDA culture

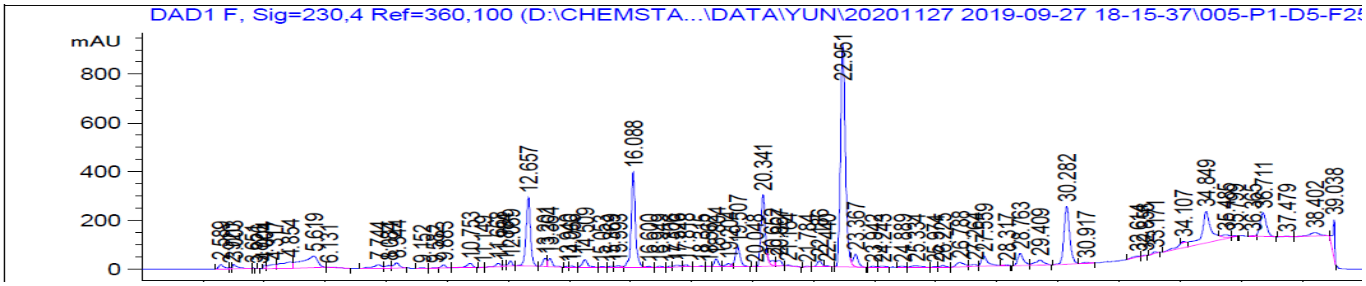

B.

Soybean-based culture

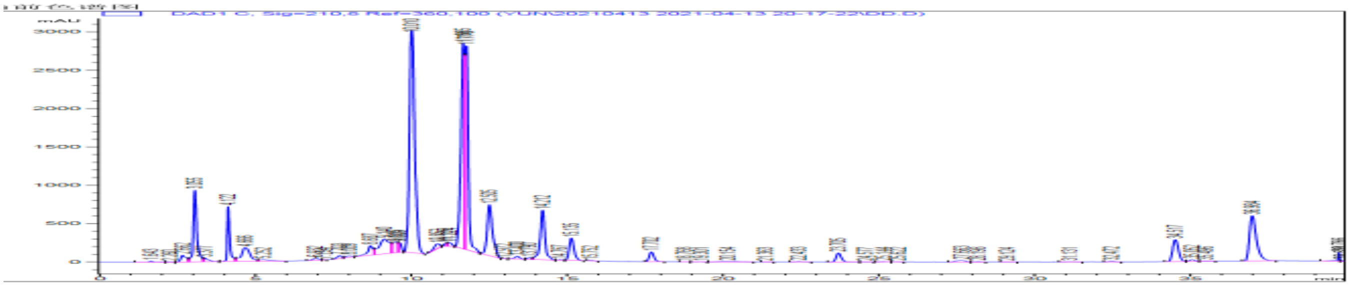

F25-Soybean- based culture

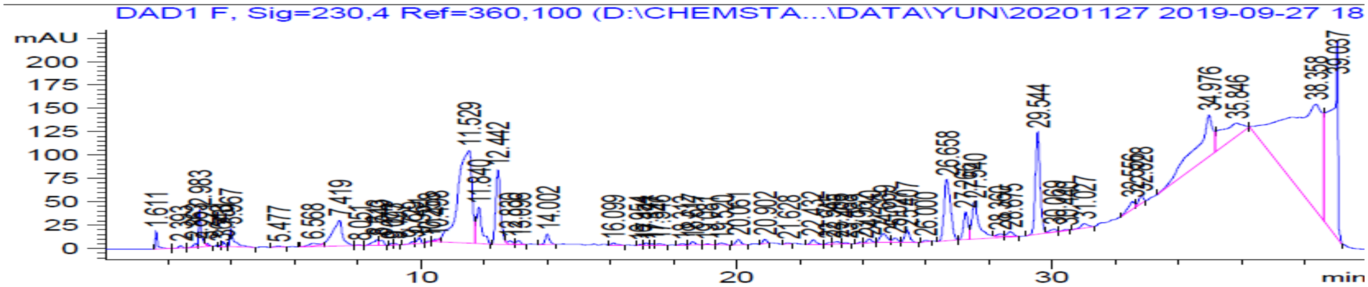

C.

Rice-based culture

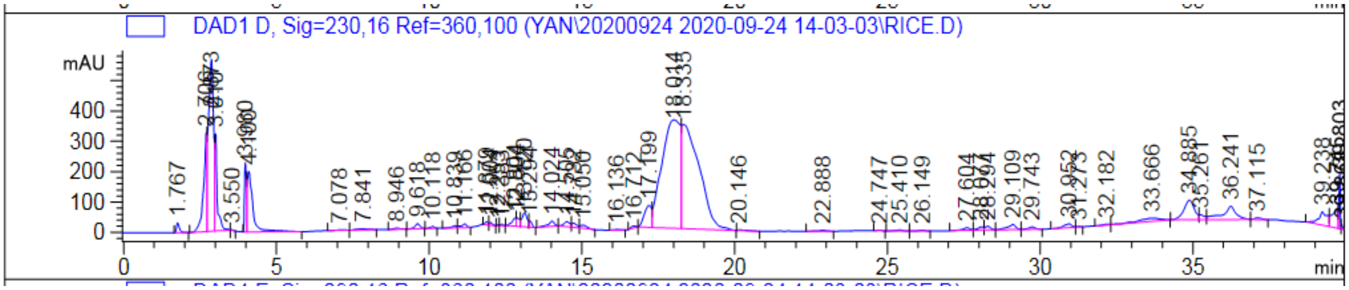

F25-Rice-based culture

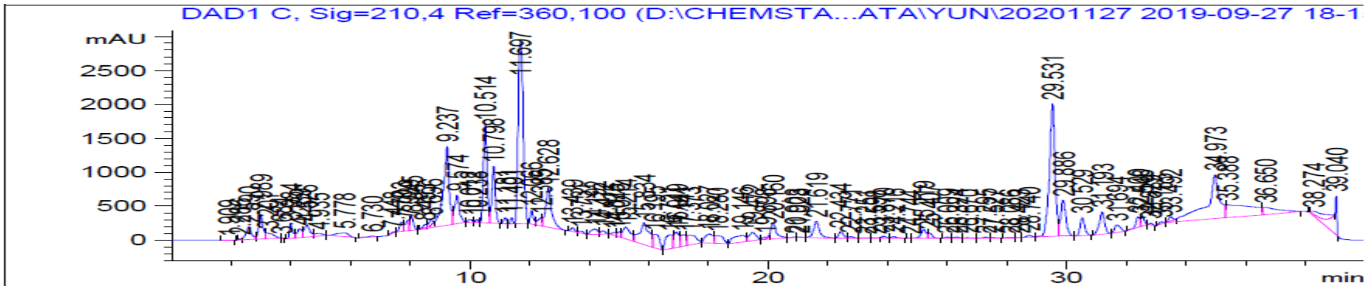

D.

Corn-based culture

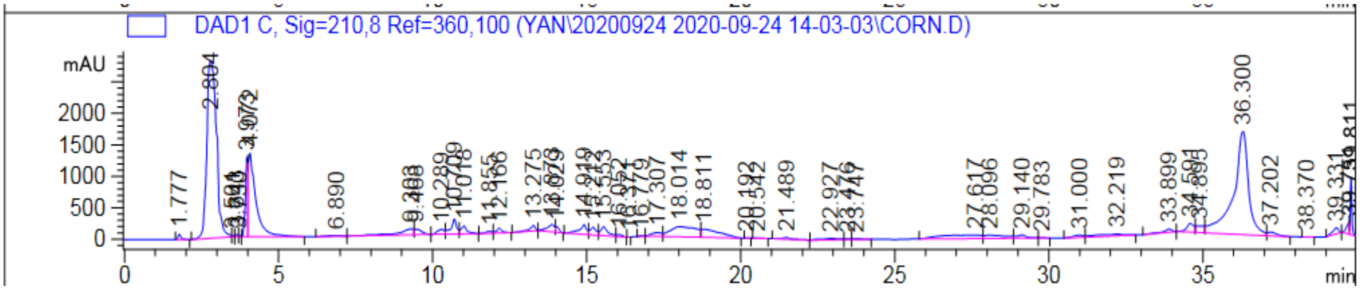

F25-Corn-based culture

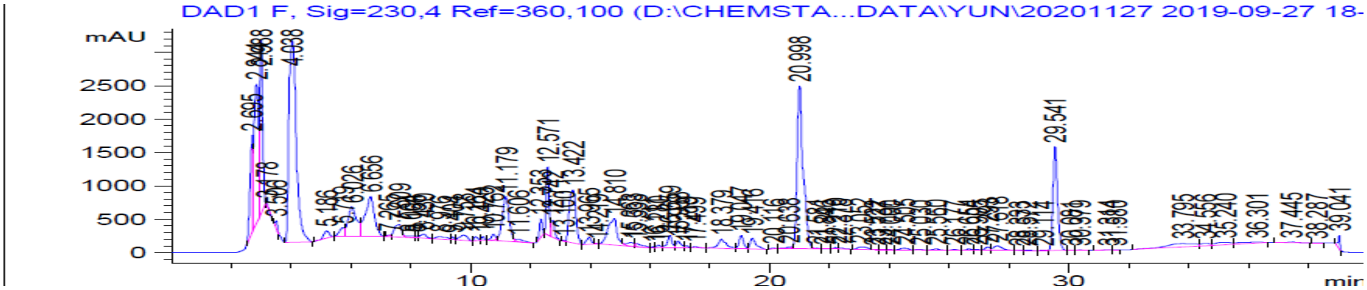

Supplementary Figure 1.The HPLC analyses of crude extracts of *Fusarium decemcellulare* F25 in different cultural conditions.

(A) The HPLC data of crude extracts of *Fusarium decemcellulare* F25 cultured in CDA culture.

(B) The HPLC data of crude extracts of *Fusarium decemcellulare* F25 cultured in soybean-based culture.

(C) The HPLC data of crude extracts of *Fusarium decemcellulare* F25 cultured in rice-based culture.

(D) The HPLC data of crude extracts of *Fusarium decemcellulare* F25 cultured in corn-based culture.

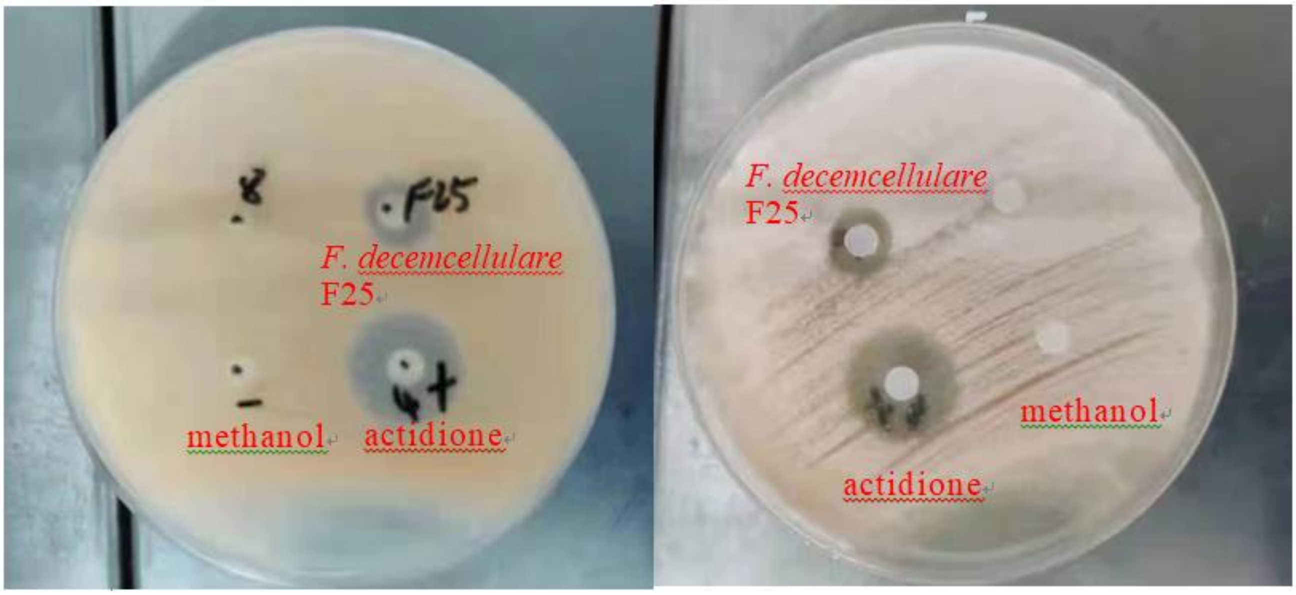

Supplementary Figure 2. The antifungal activities of **c**rude extracts were evaluated against *Colletotrichum musa*e ACCC 31244 (Positive control: cycloheximide; Negative control: methanol) .

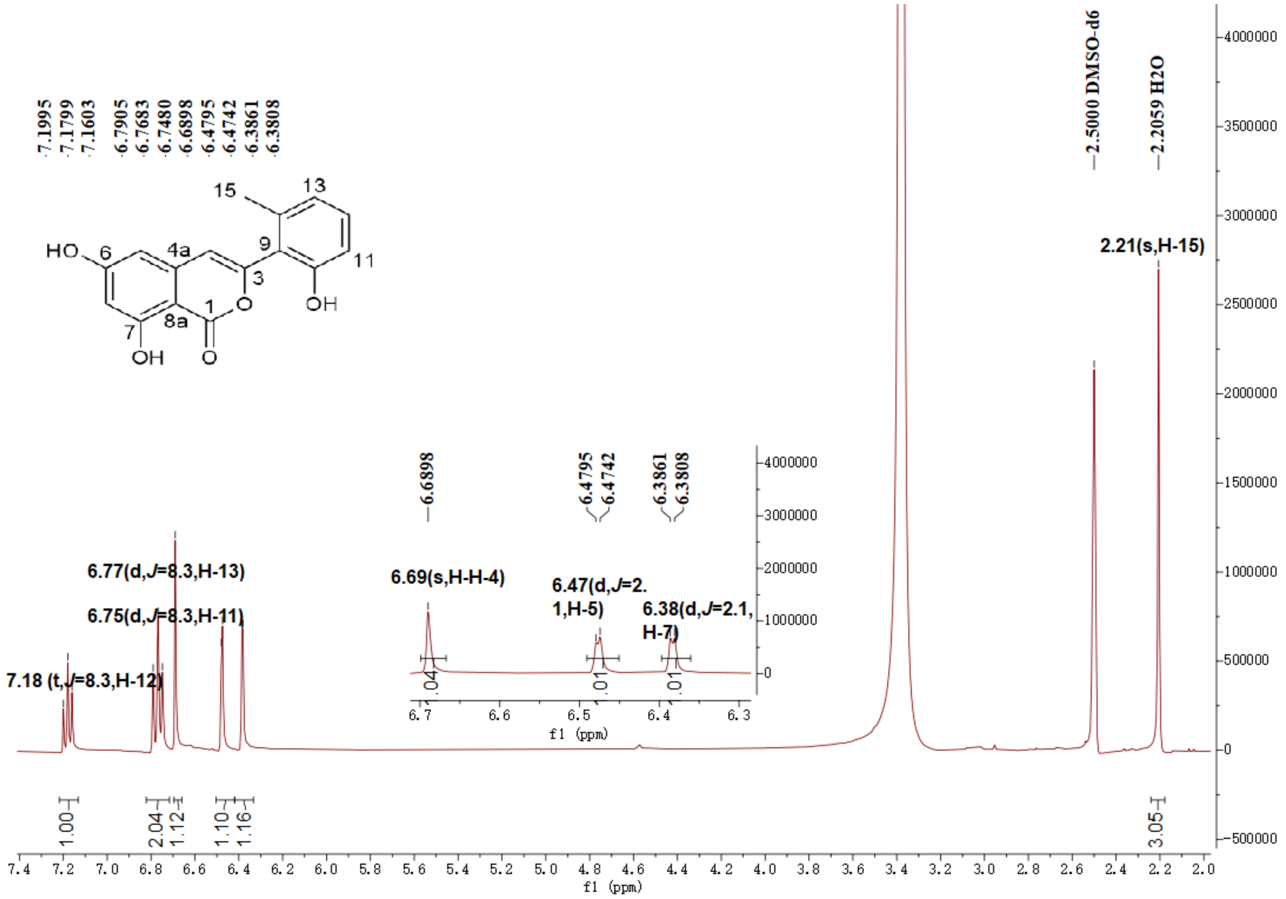

Supplementary Figure 3. The ^{1}H NMR (400 MHz, DMSO-d_6) spectrum of compound **1**.

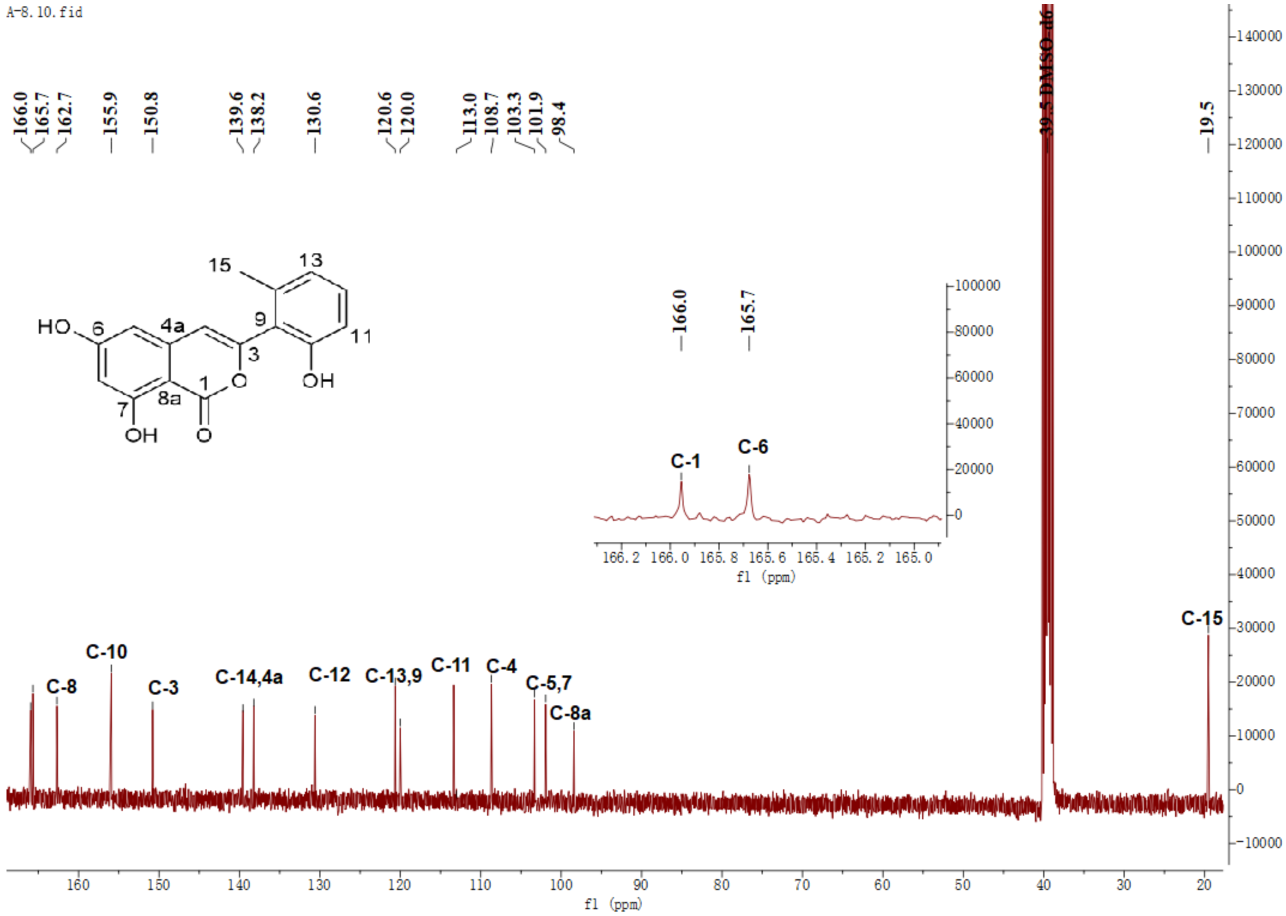

Supplementary Figure 4. The ^{13}C NMR (100 MHz, DMSO-d_6) spectrum of compound **1**.

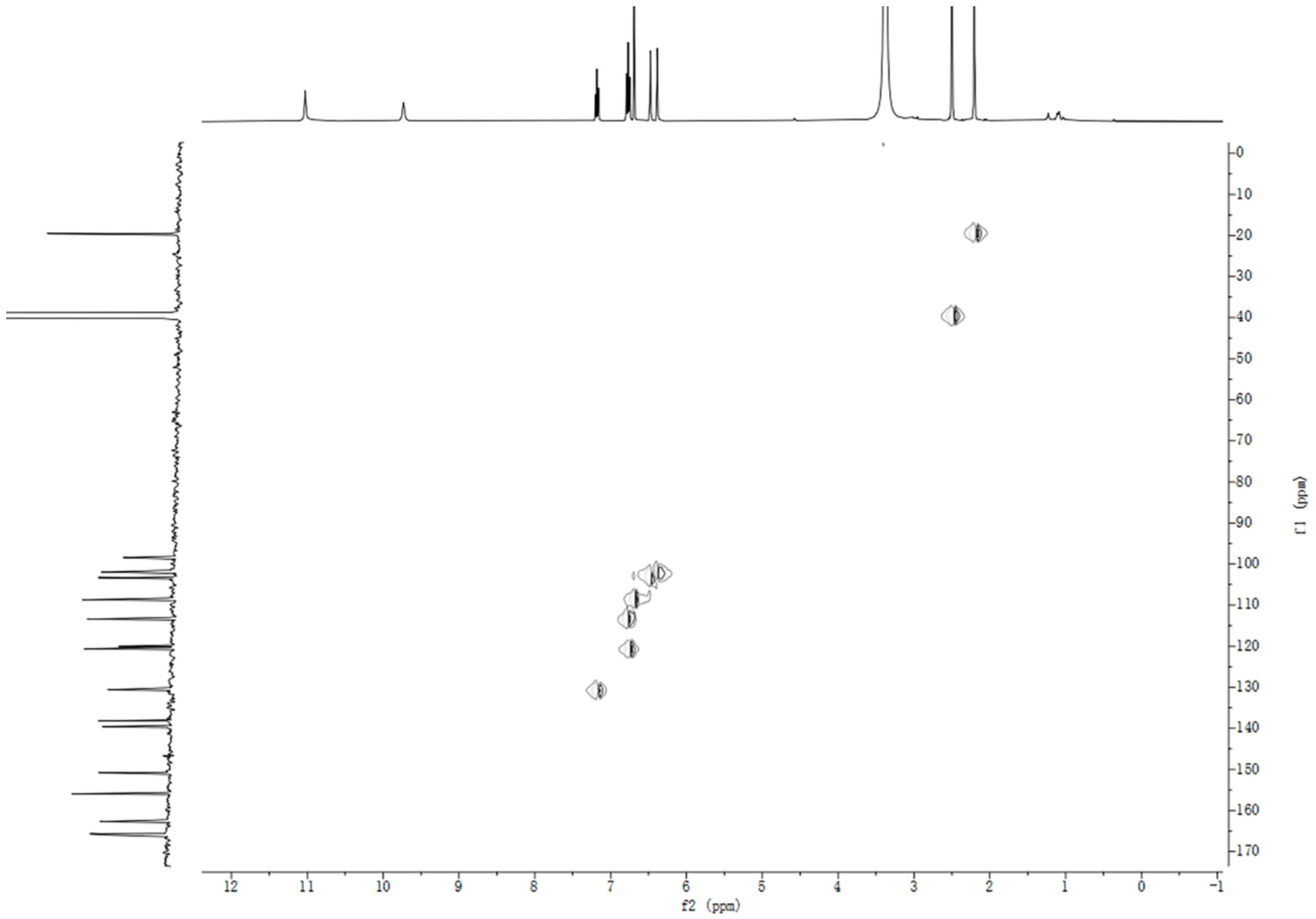

Supplementary Figure 5. The HSQC spectrum of compound **1**.

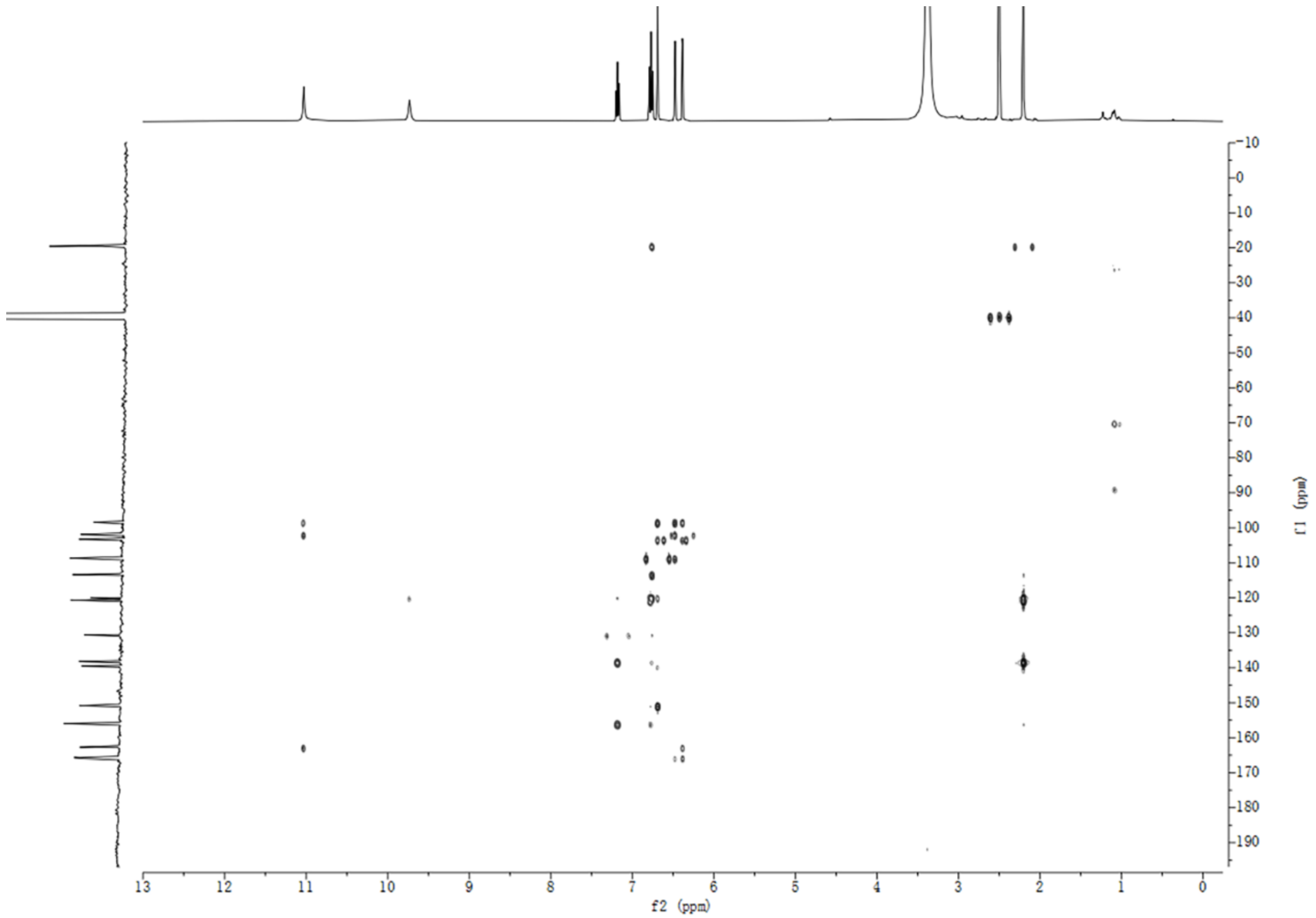

Supplementary Figure 6. The HMBC spectrum of compound **1**.

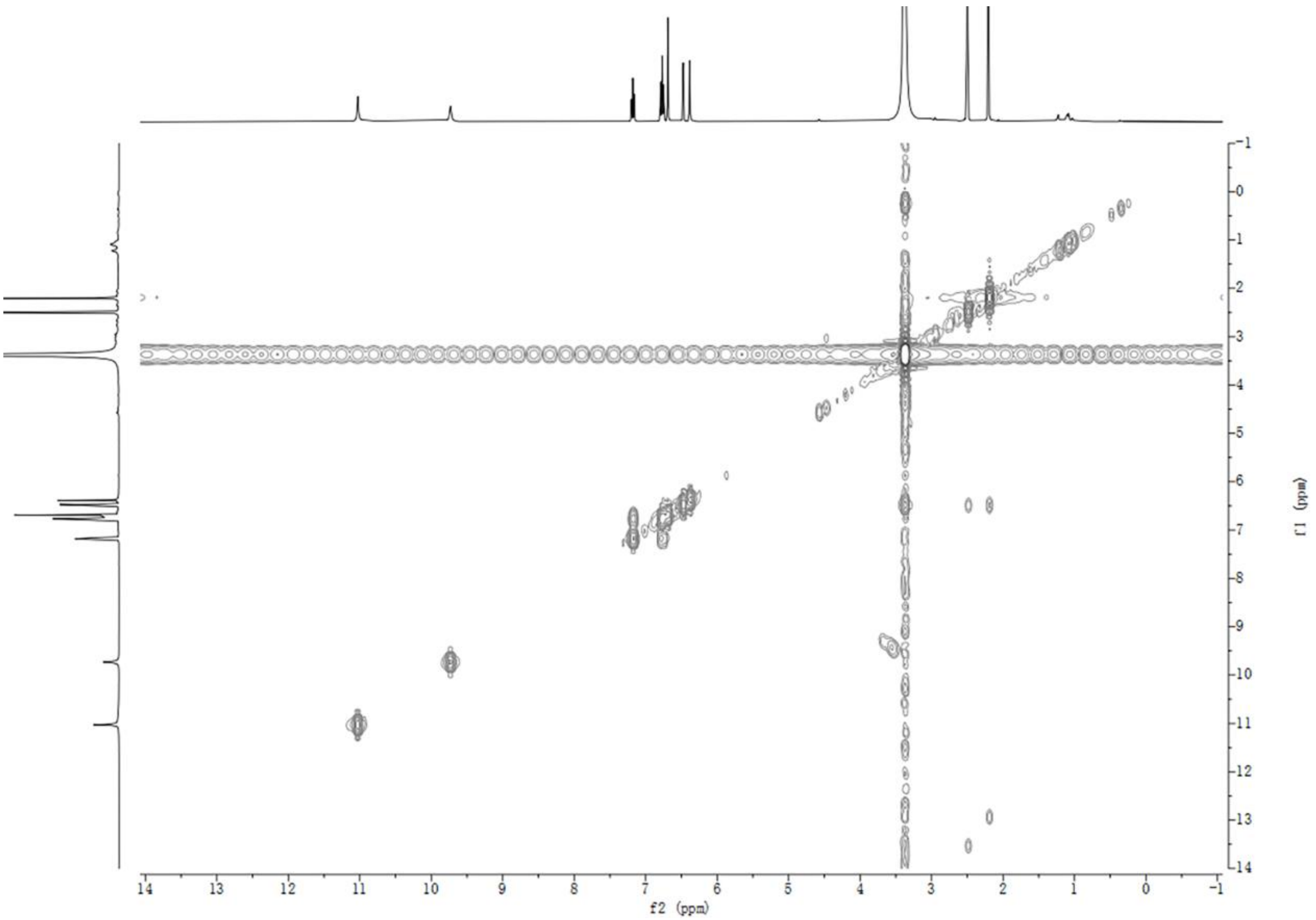

Supplementary Figure 7. The COSY spectrum of compound **1**.

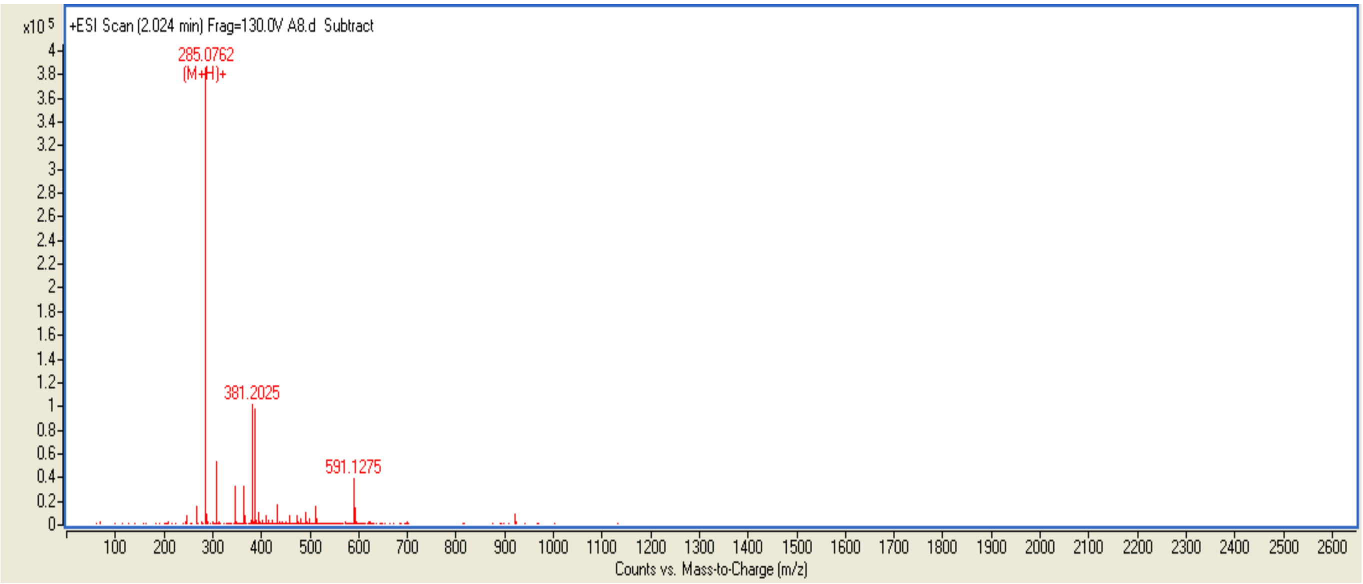

Supplementary Figure 8. The ESI-HRMS spectrum of compound **1**.

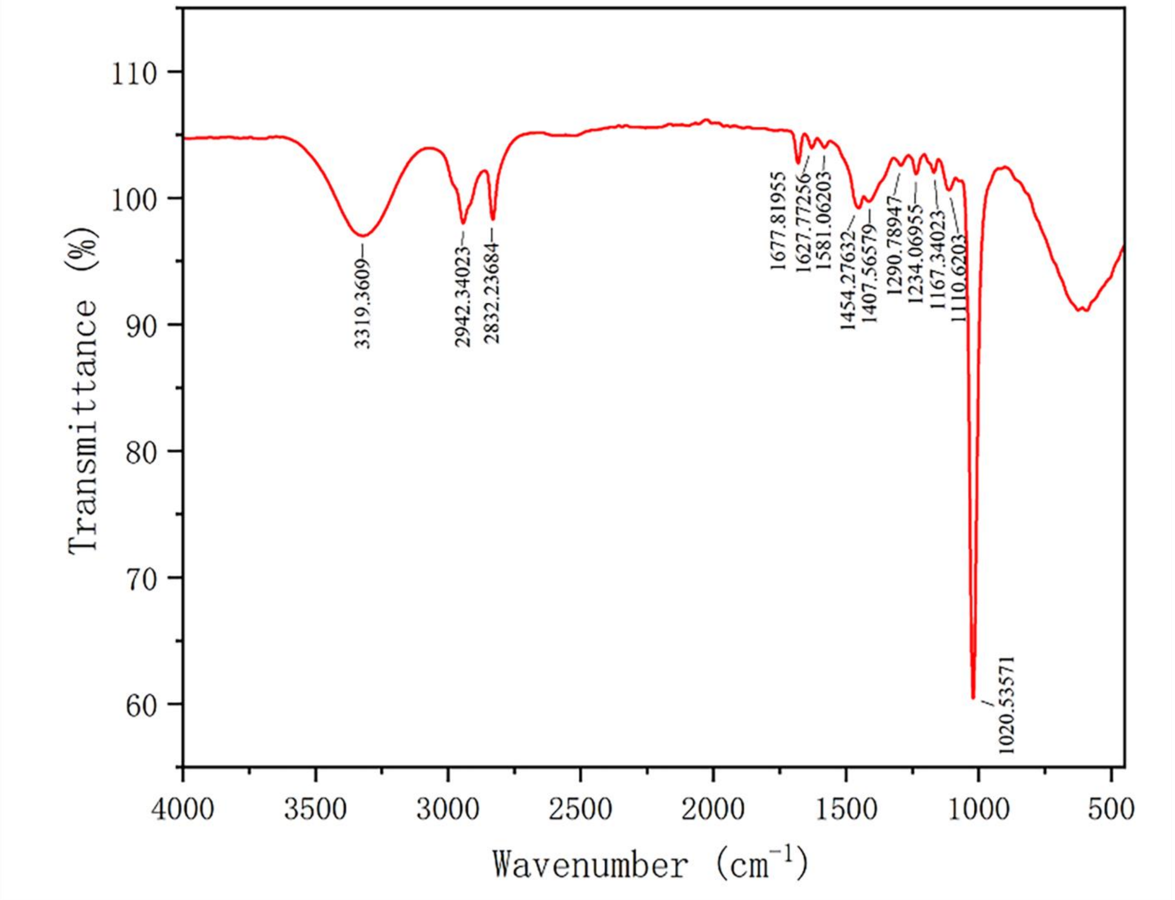

Supplementary Figure 9. IR spectrum of compound **1**.

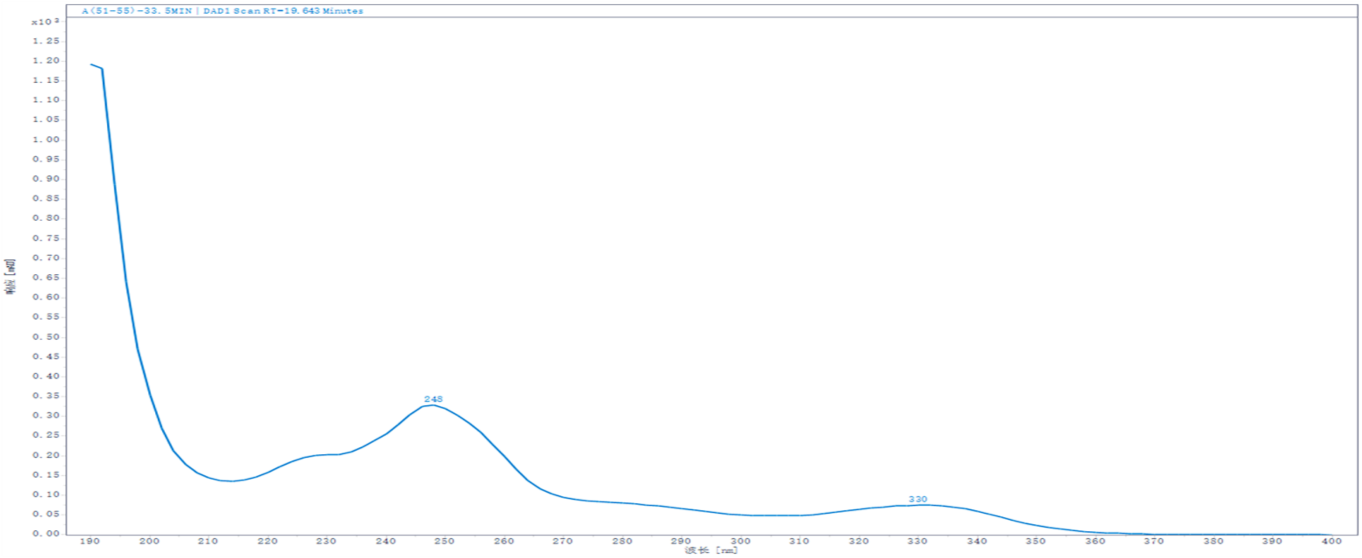

Supplementary Figure 10. UV spectrum of compound **1**.

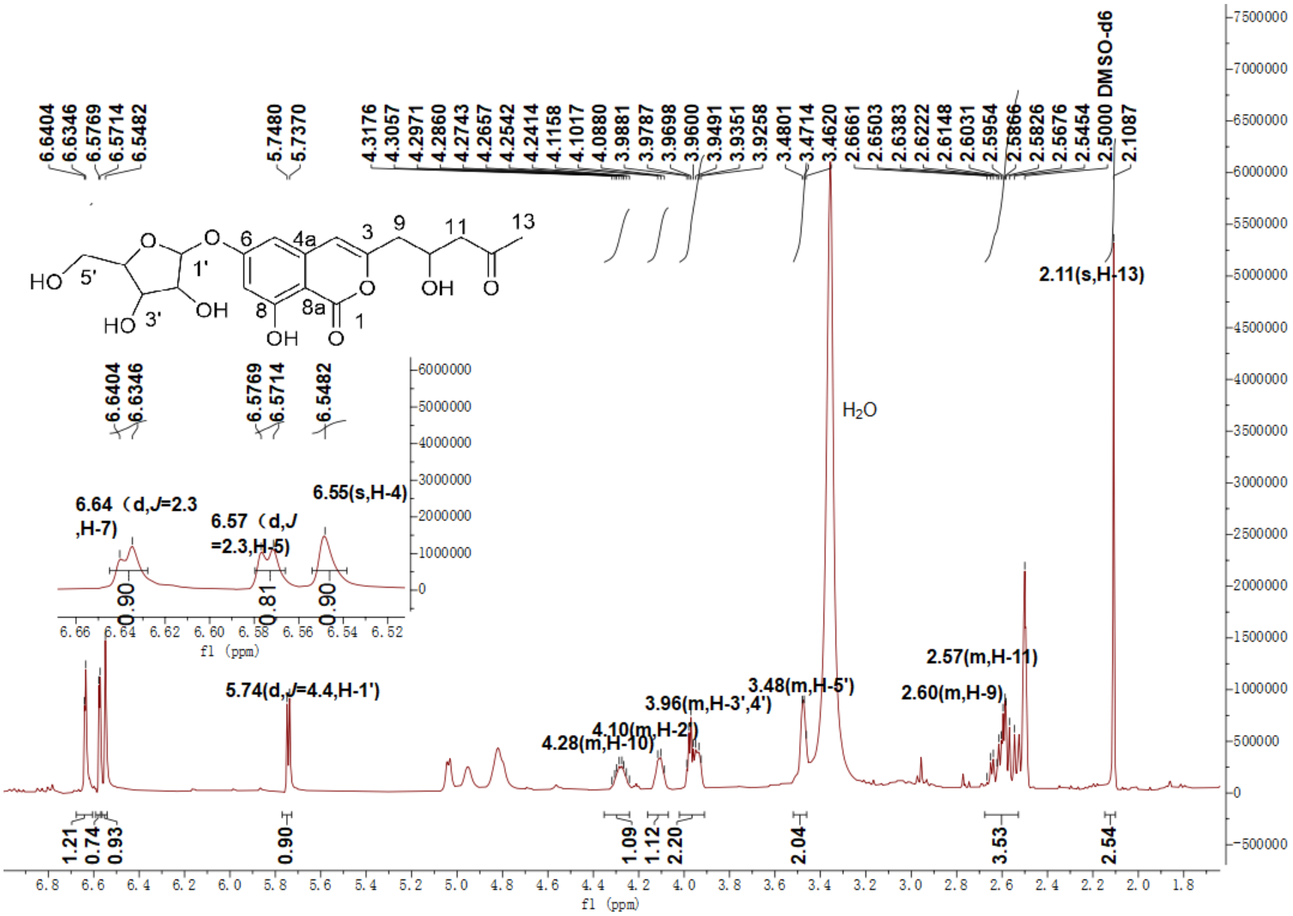

Supplementary Figure 11. The ^{1}H NMR (400 MHz, DMSO-d_6) spectrum of compound **2**.

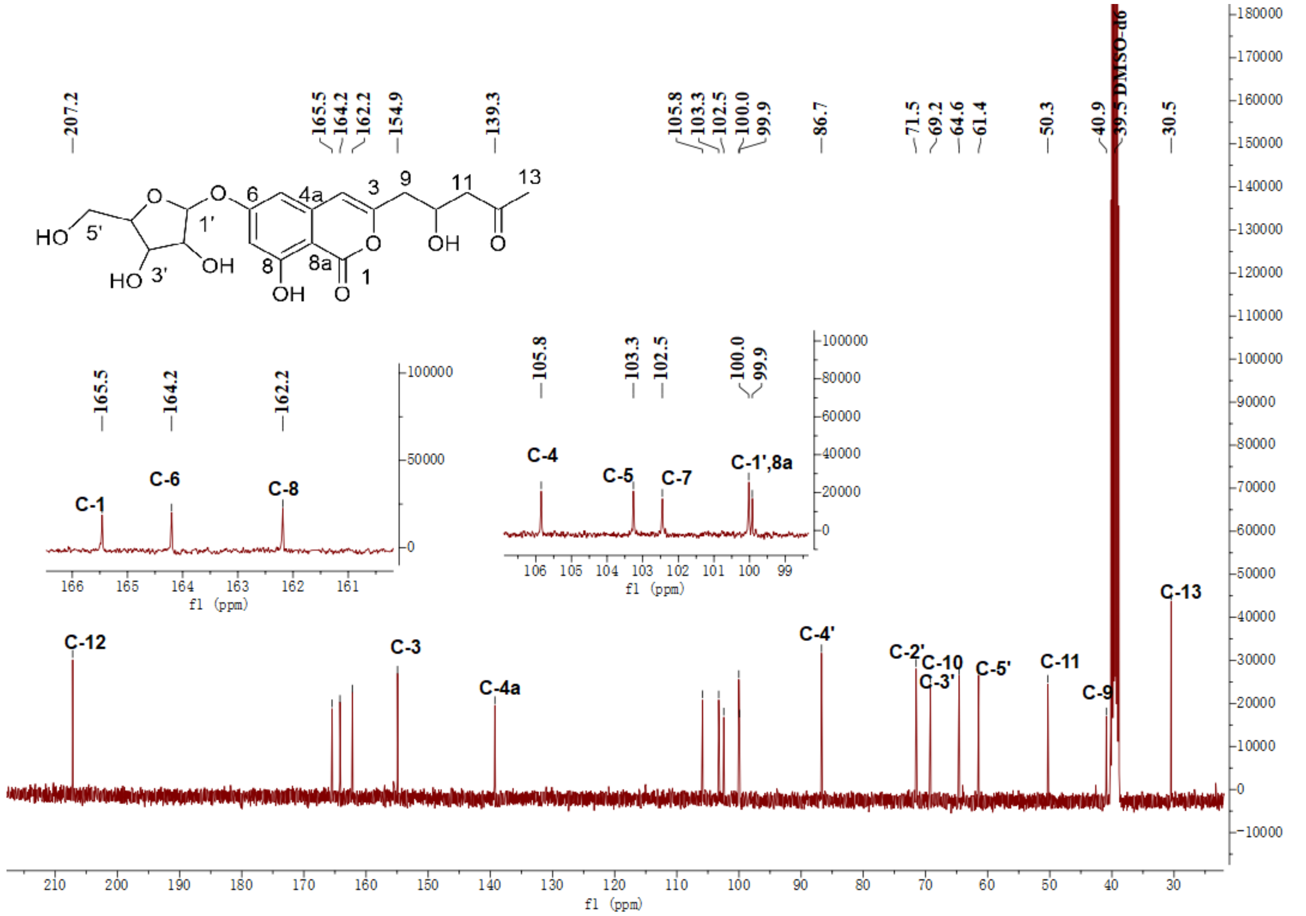

Supplementary Figure 12. The ^{13}C NMR (100 MHz, DMSO-d_6) spectrum of compound **2**.

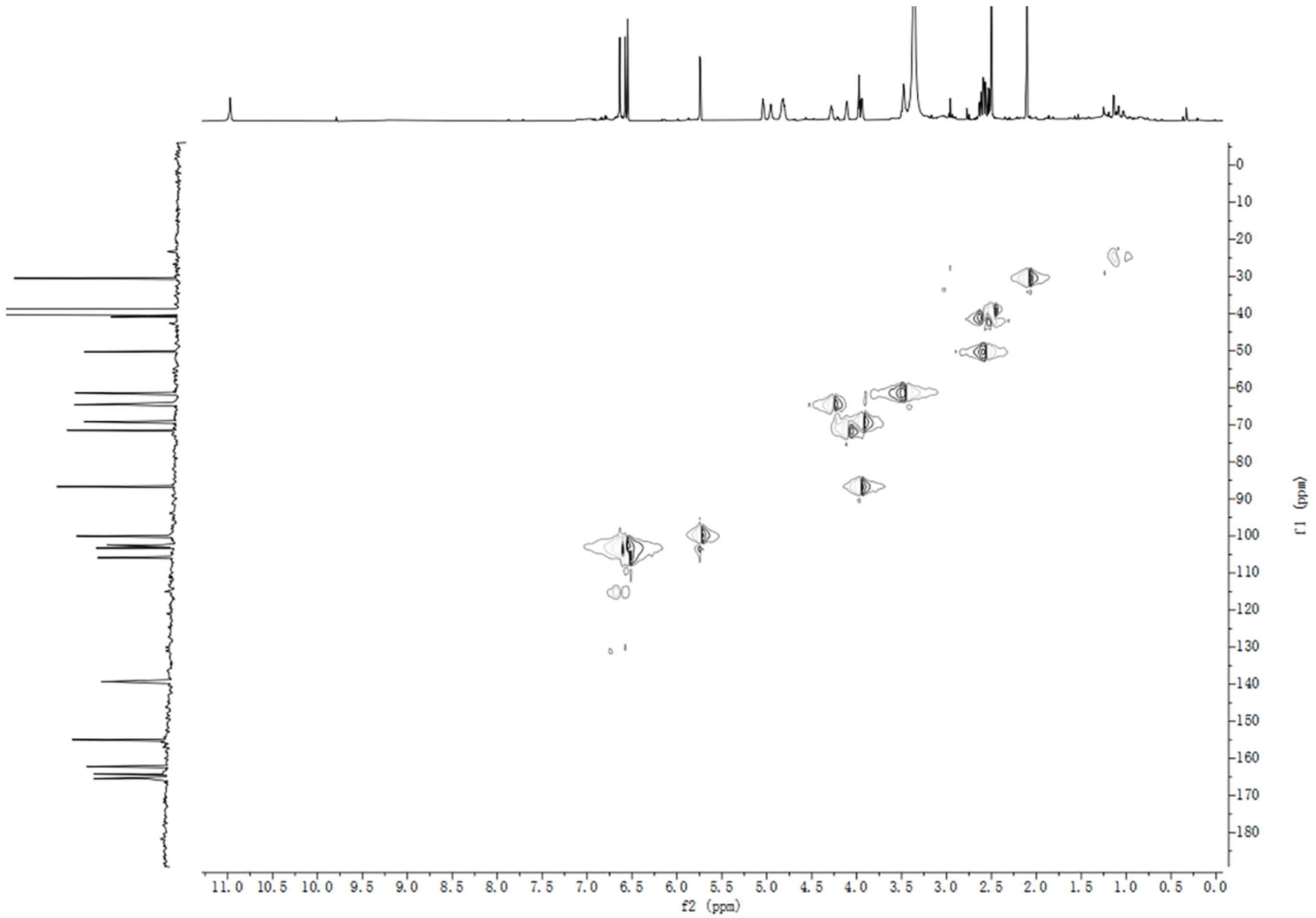

Supplementary Figure 13. The HSQC spectrum of compound **2**.

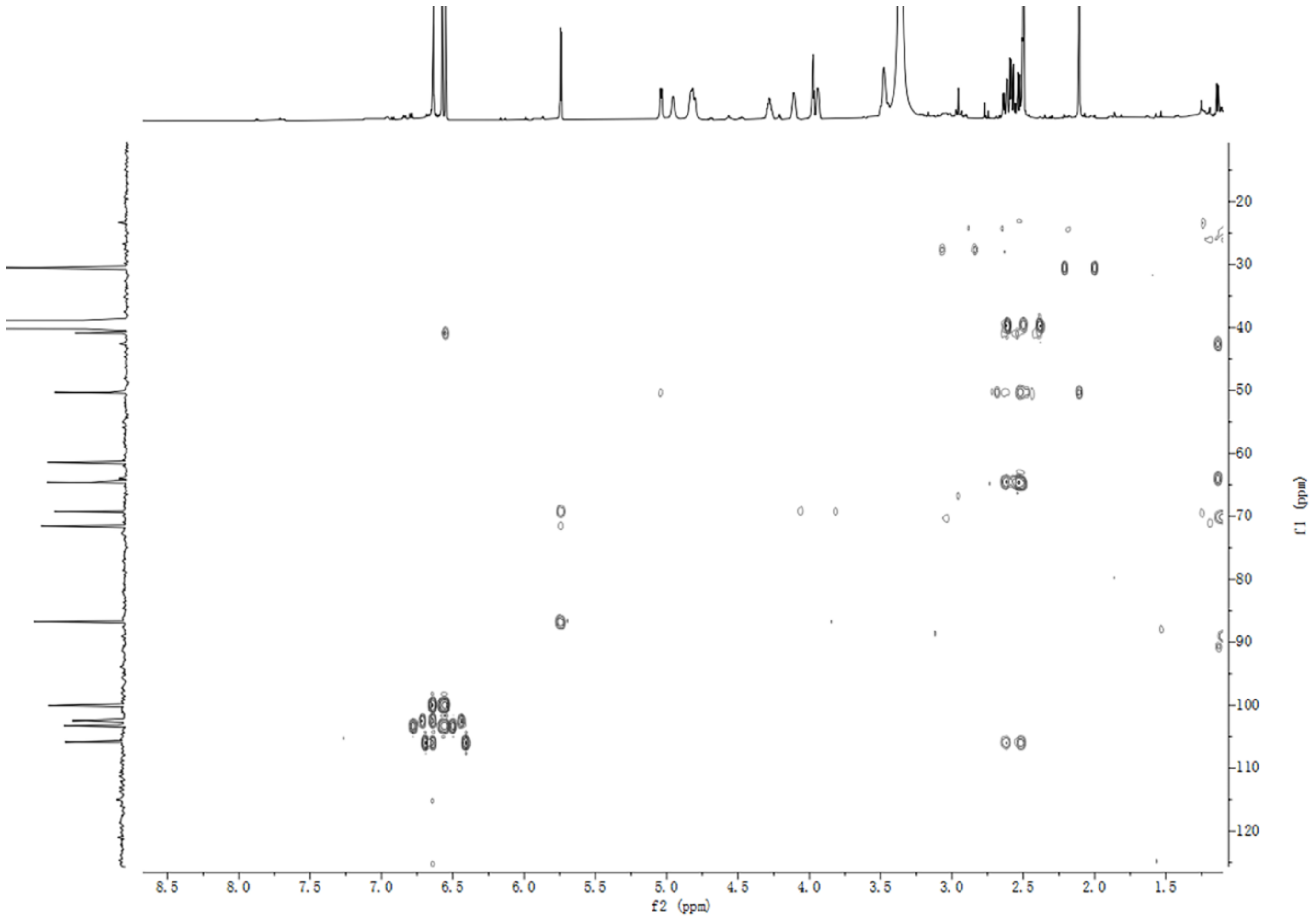

Supplementary Figure 14. The HMBC spectrum of compound **2**.

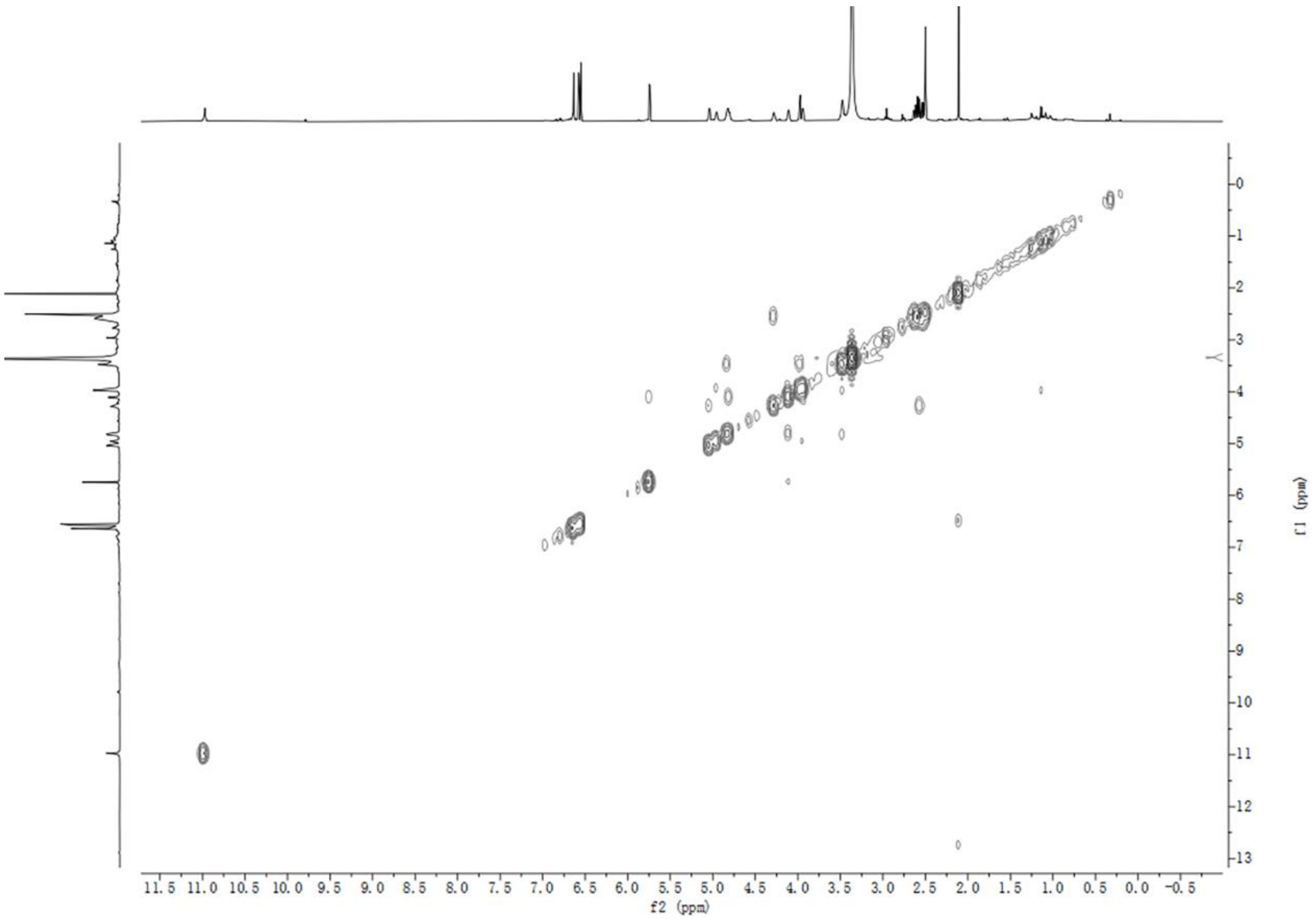

Supplementary Figure 15. The COSY spectrum of compound **2**.

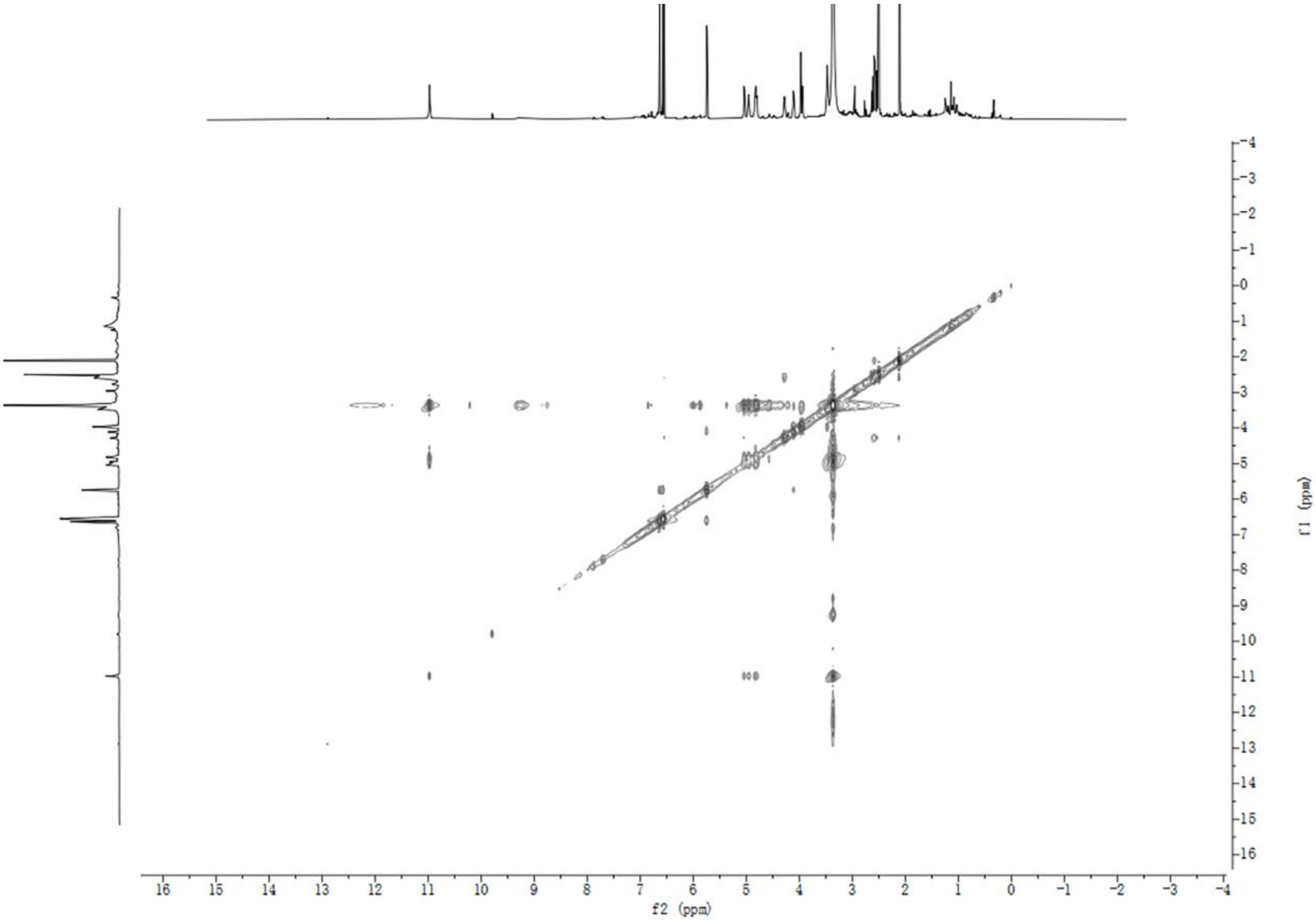

Supplementary Figure 16. The NOESY spectrum of compound **2**.

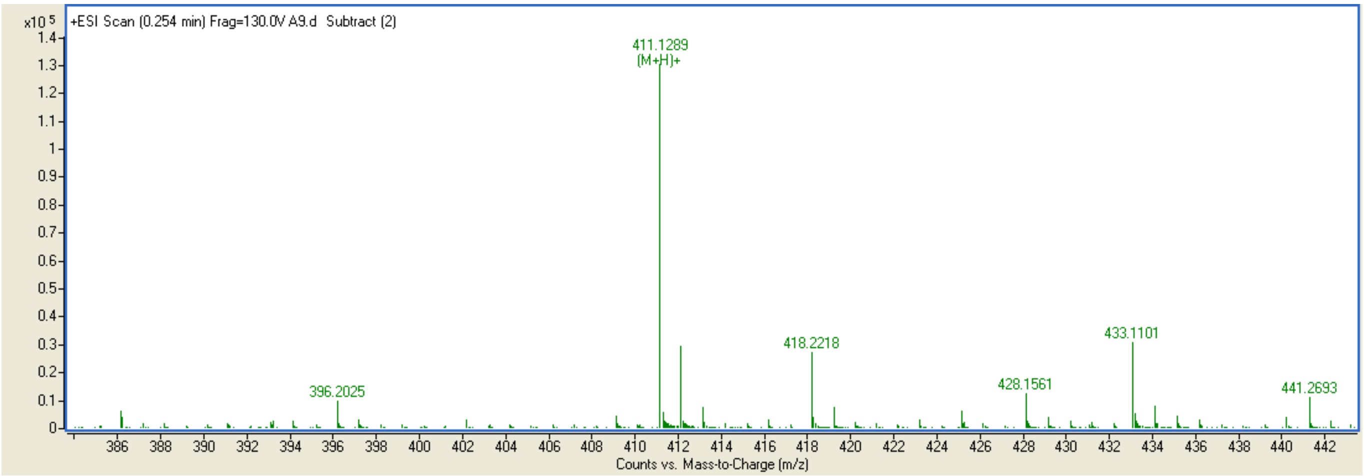

Supplementary Figure 17. The ESI-HRMS spectrum of compound **2**.

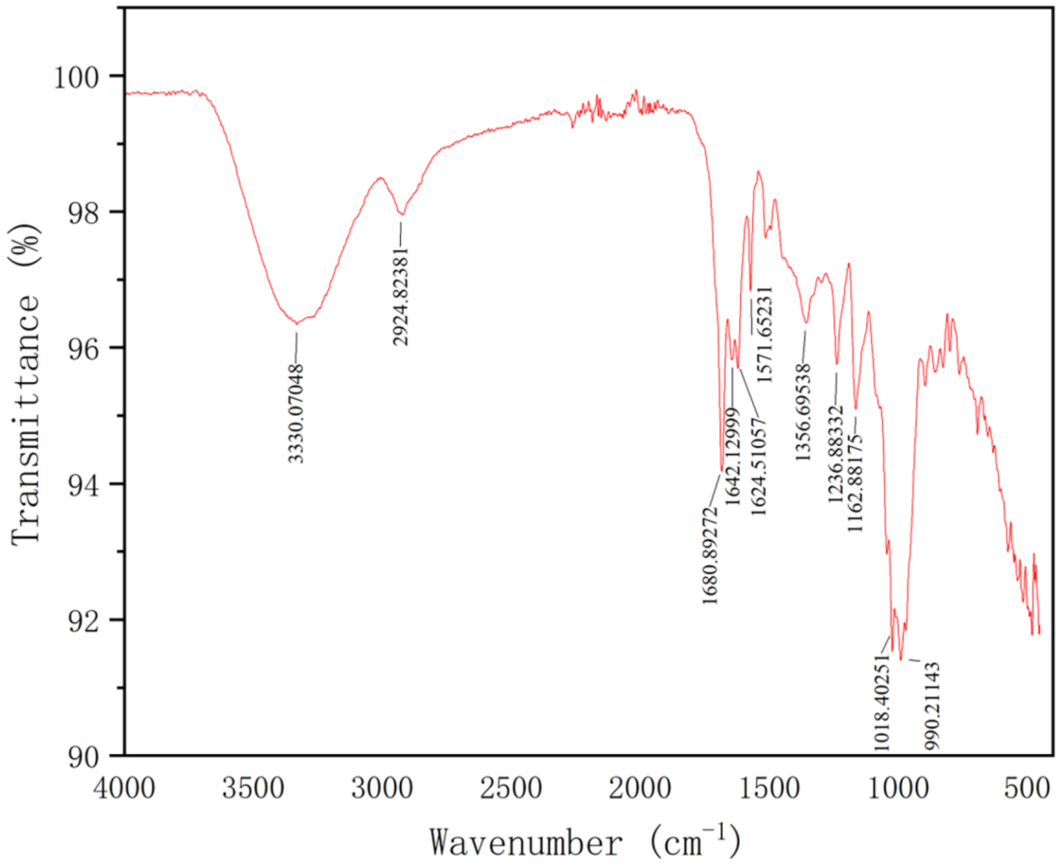

Supplementary Figure 18. IR spectrum of compound **2**.

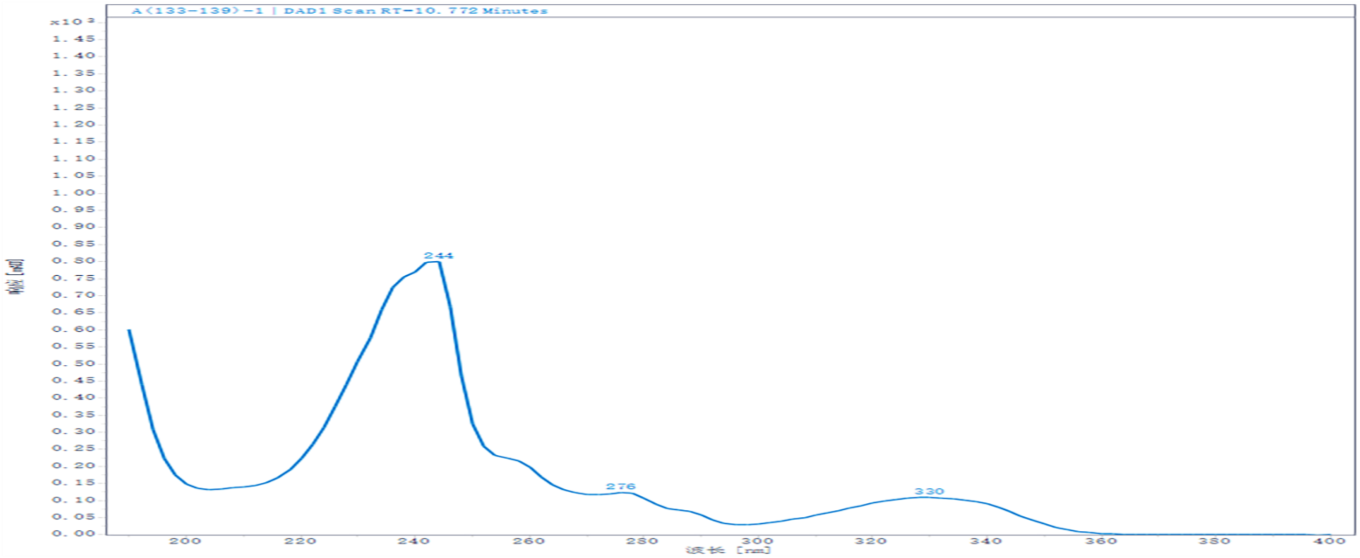

Supplementary Figure 19. UV spectrum of compound **2**.

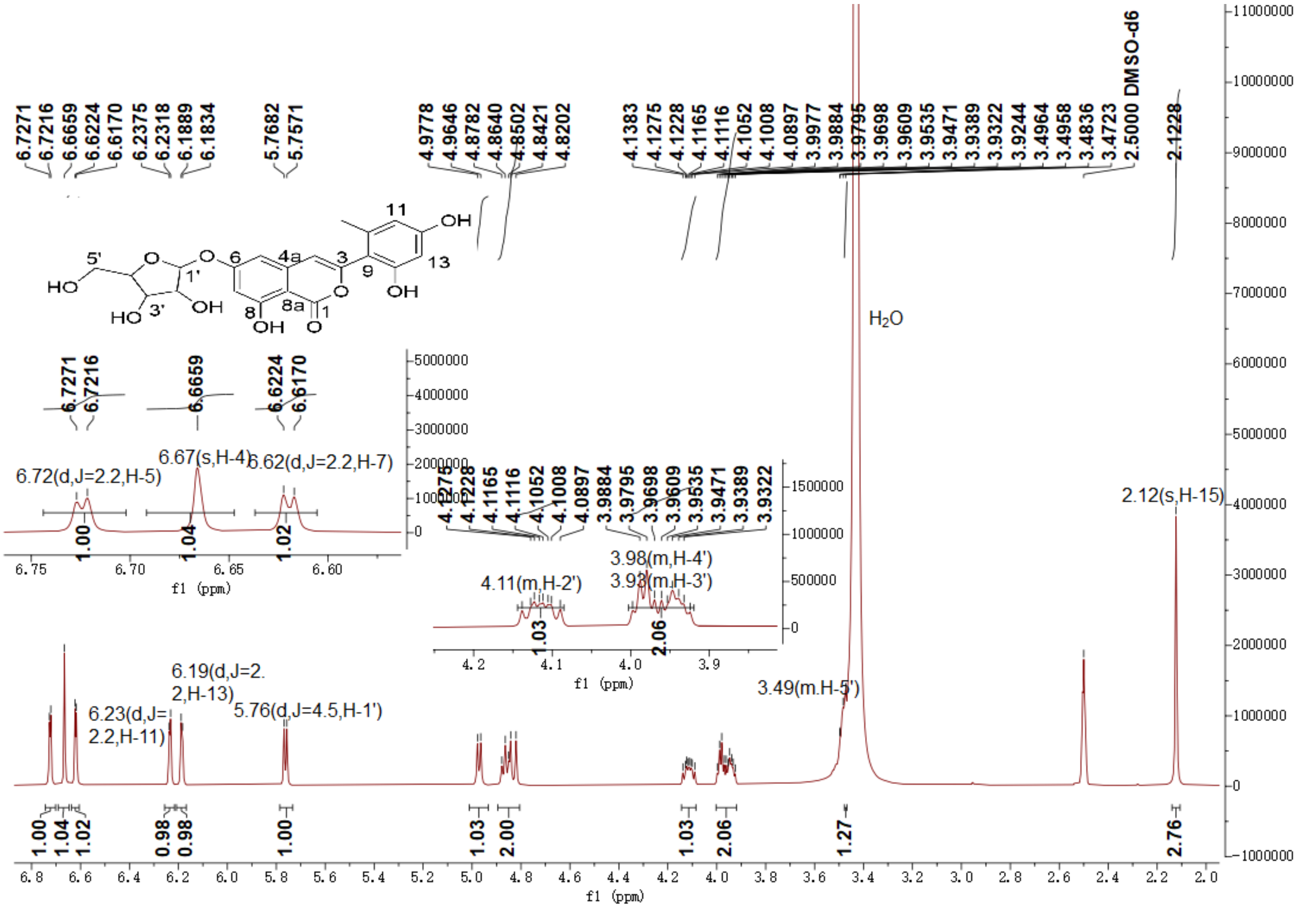

Supplementary Figure 20. The ^{1}H NMR (400 MHz, DMSO-d_6) spectrum of compound **3**.

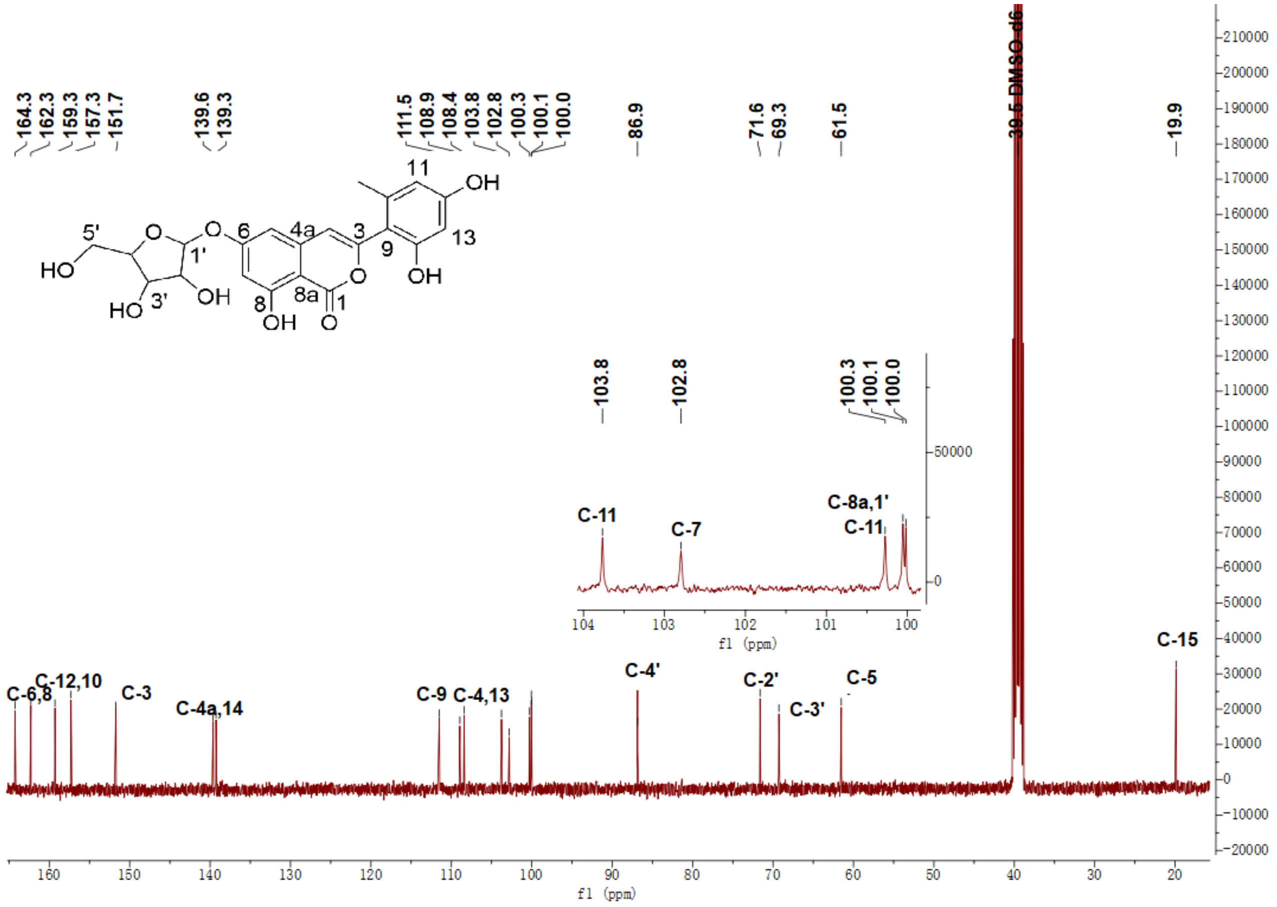

Supplementary Figure 21. The ^{13}C NMR (100 MHz, DMSO-d_6) spectrum of compound **3**.

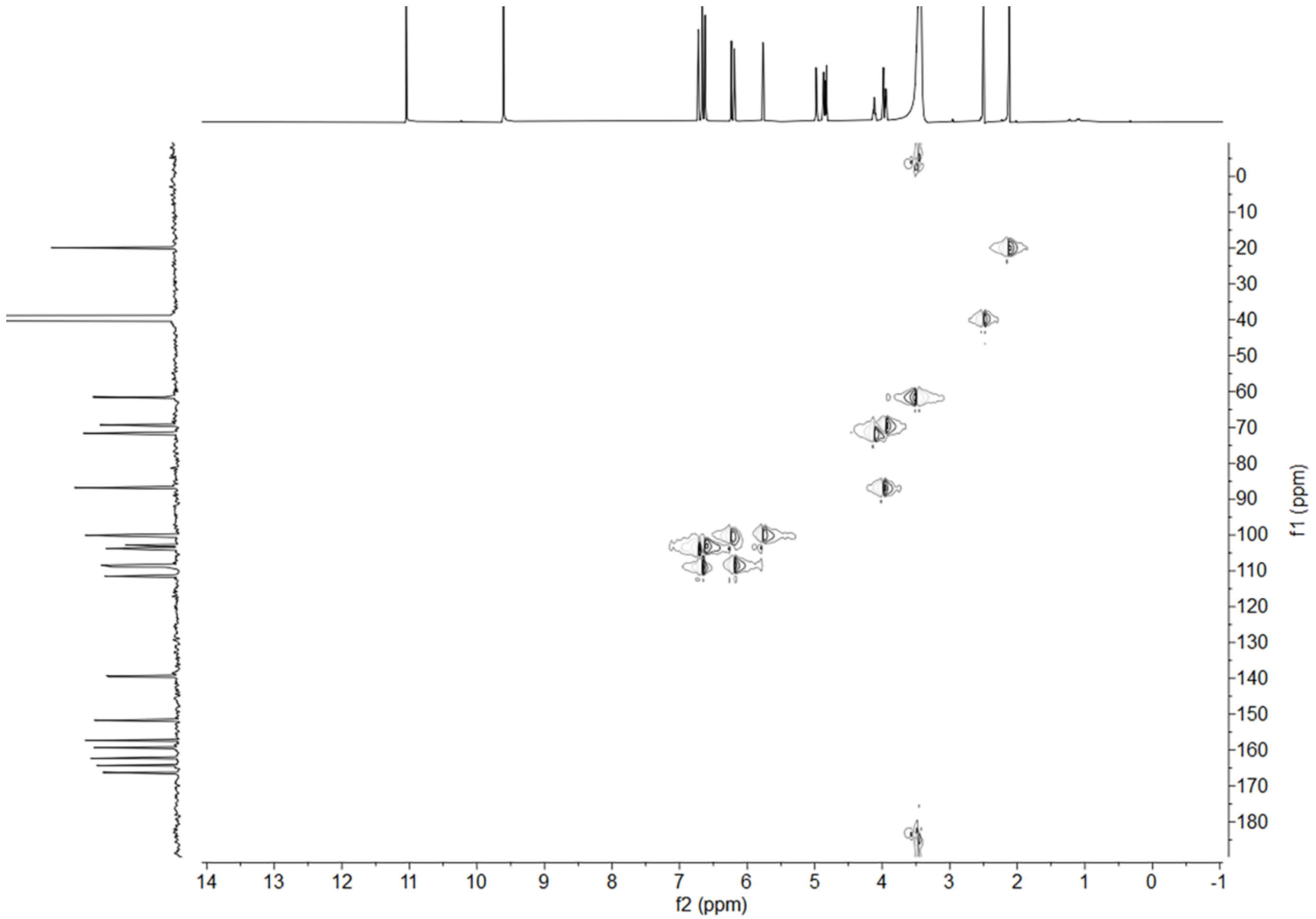

Supplementary Figure 22. The HSQC spectrum of compound **3**.

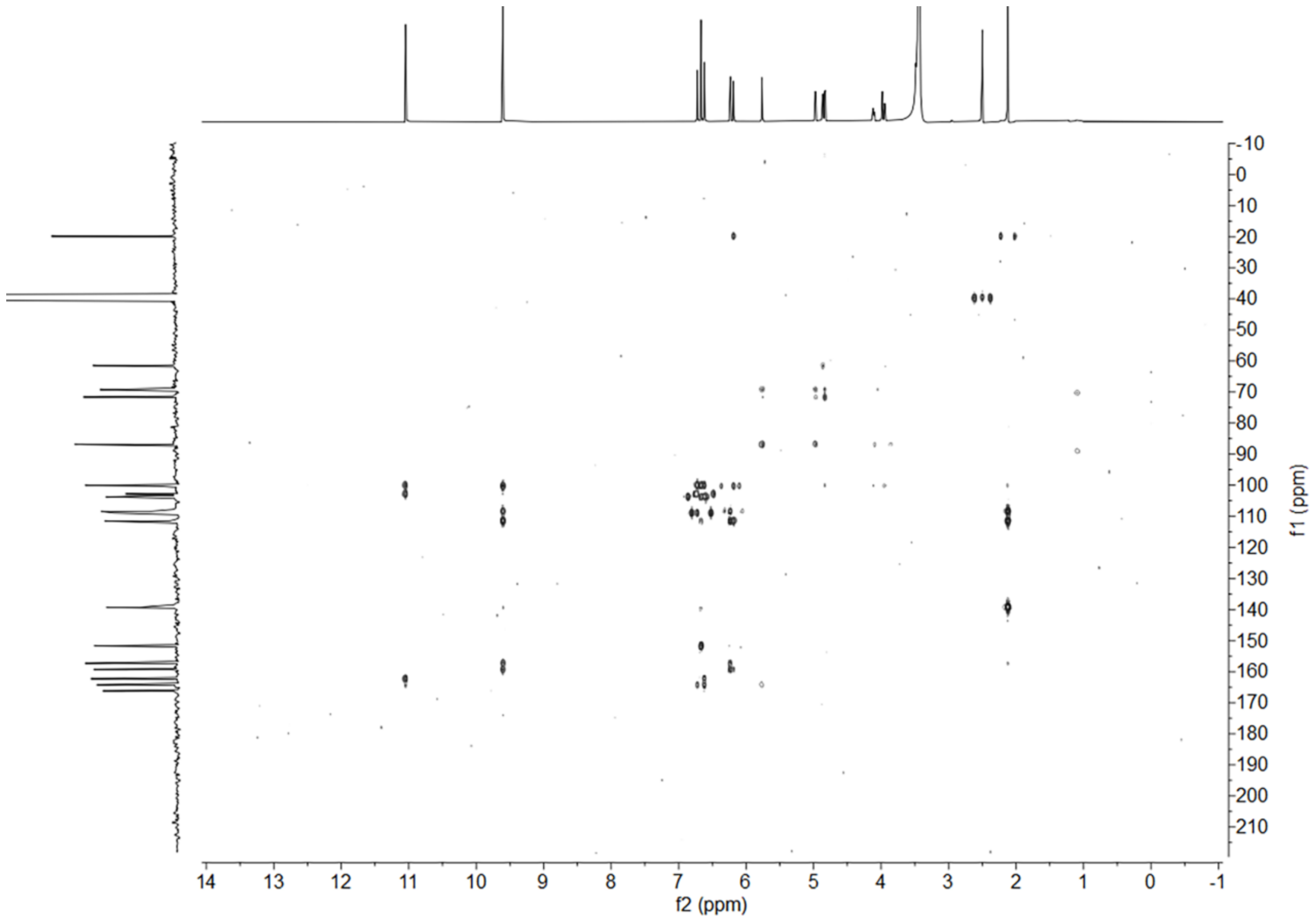

Supplementary Figure 23. The HMBC spectrum of compound **3**.

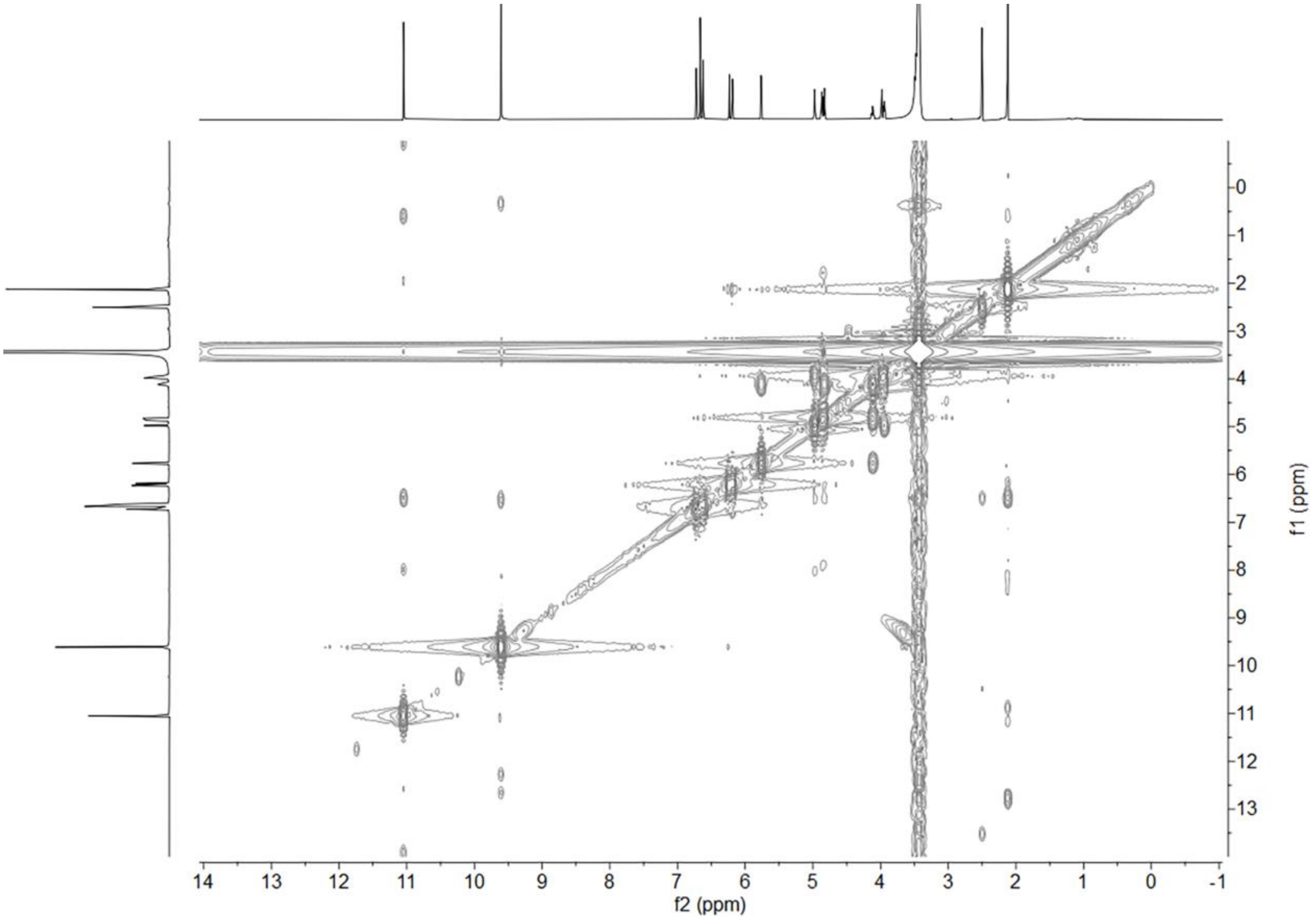

Supplementary Figure 24. The COSY spectrum of compound **3**.

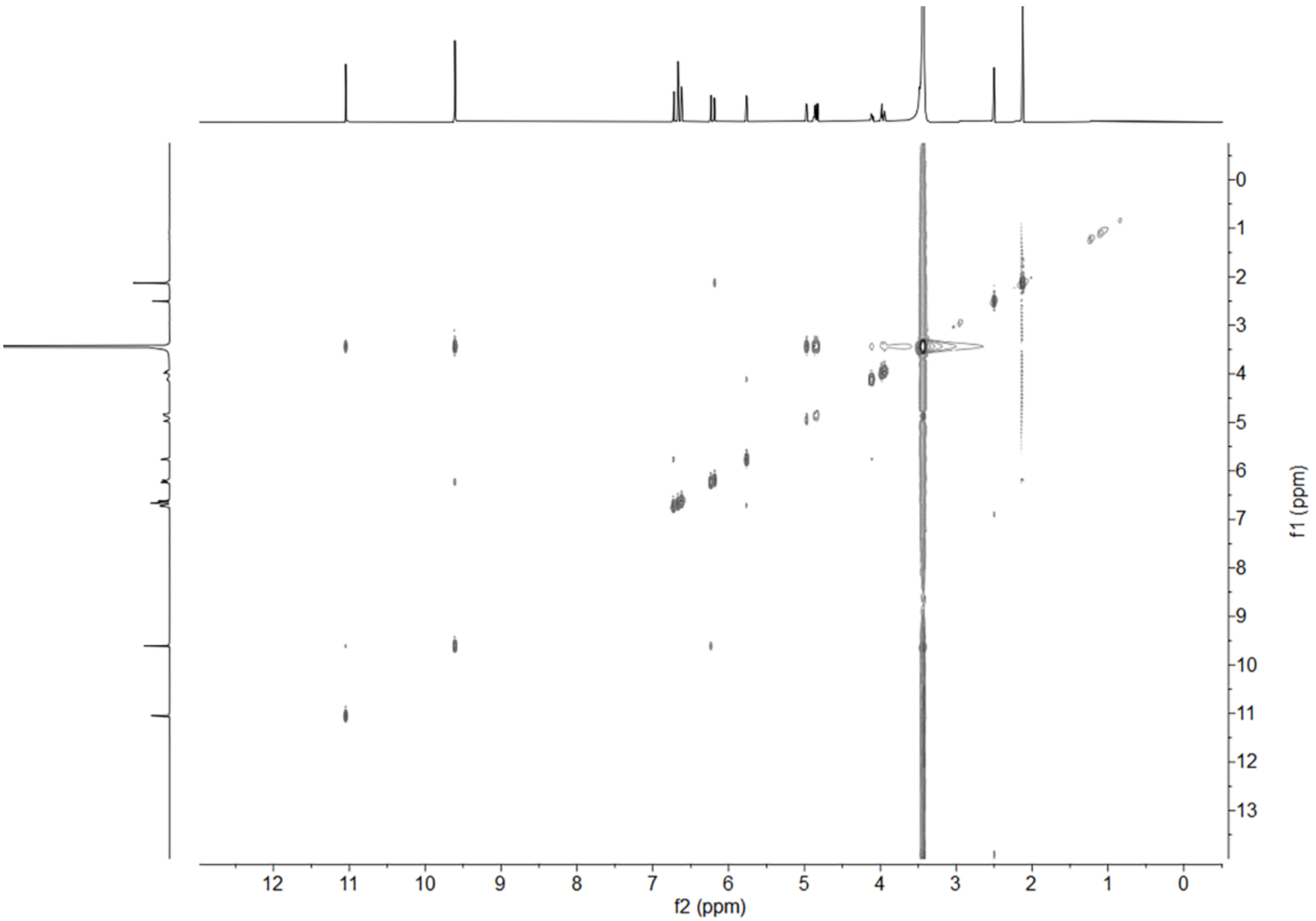

Supplementary Figure 25. The NOESY spectrum of compound **3**.

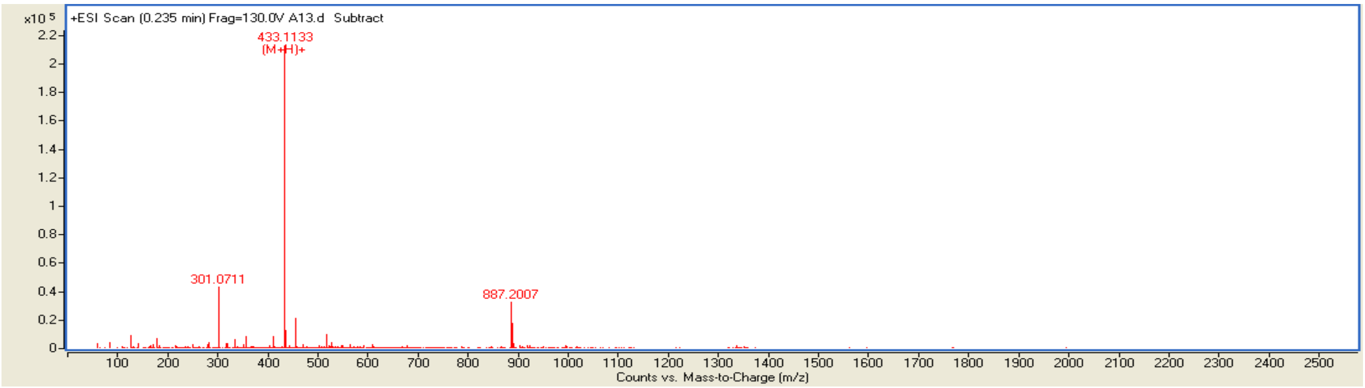

Supplementary Figure 26. The ESI-HRMS spectrum of compound **3**.

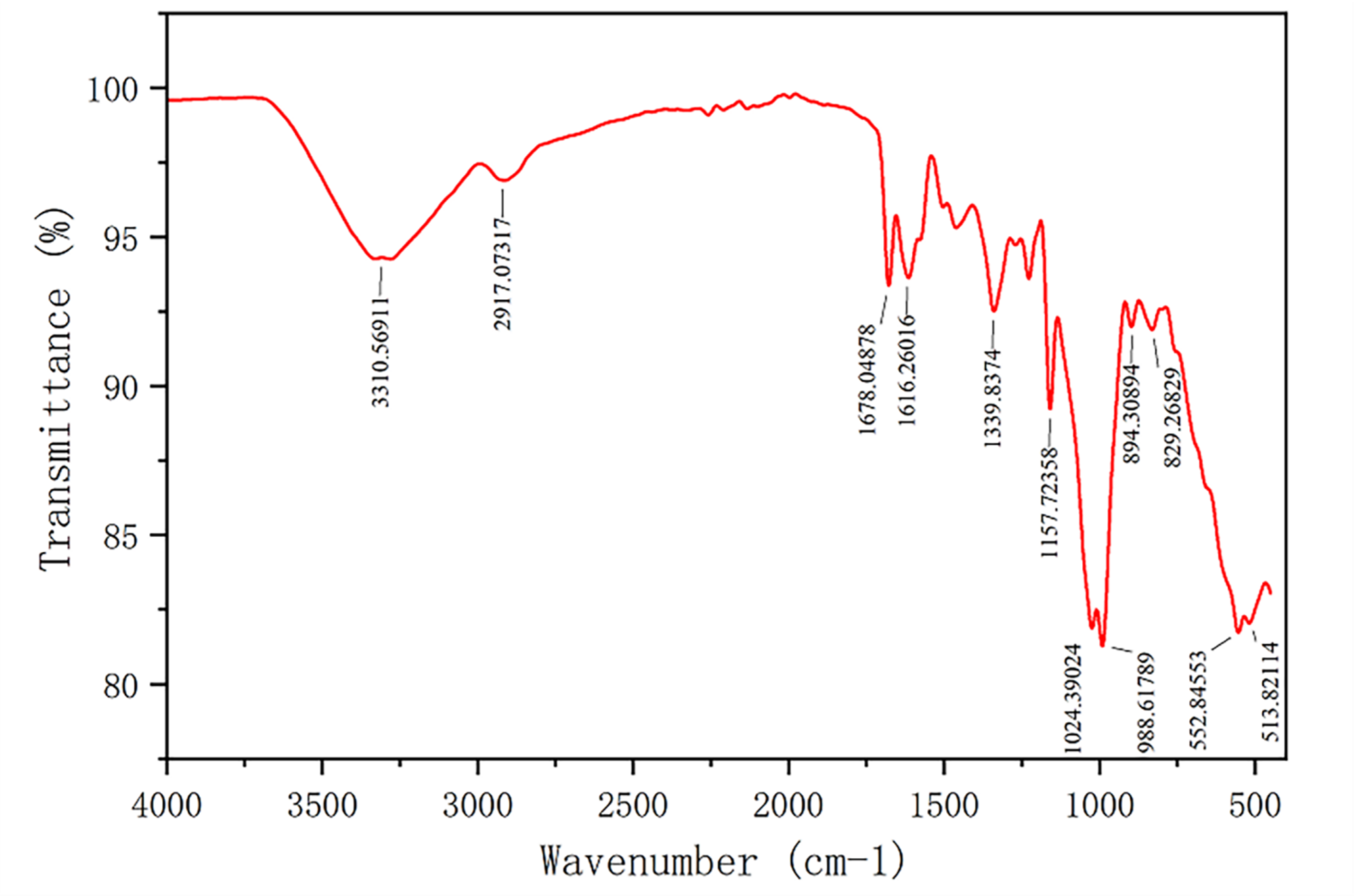

Supplementary Figure 27. IR spectrum of compound **3**.

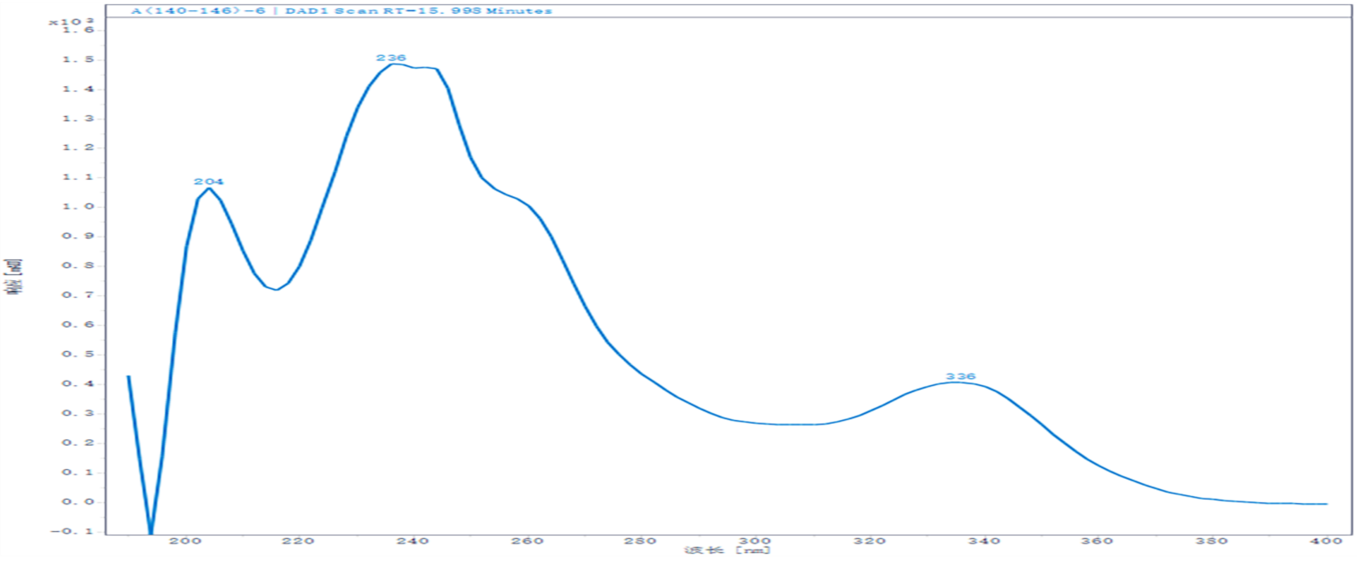

Supplementary Figure 28. UV spectrum of compound **3**.

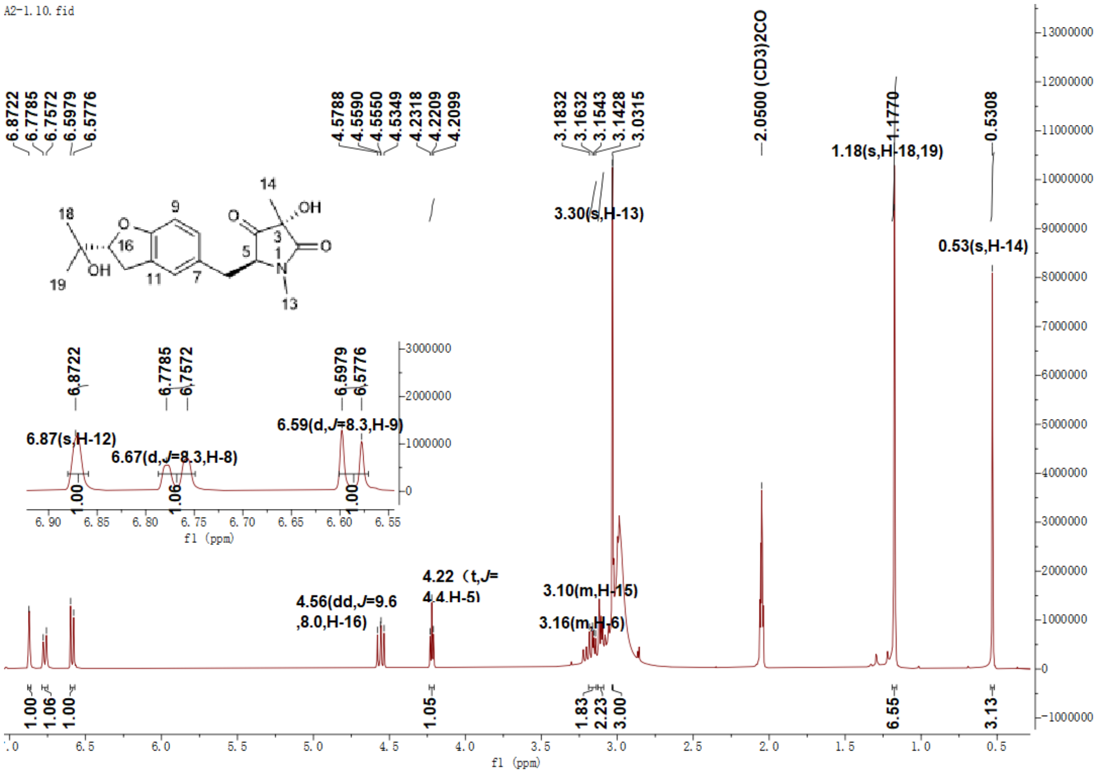

Supplementary Figure 29. The ^{1}H NMR (400 MHz, acetone-d_6) spectrum of compound **8**.

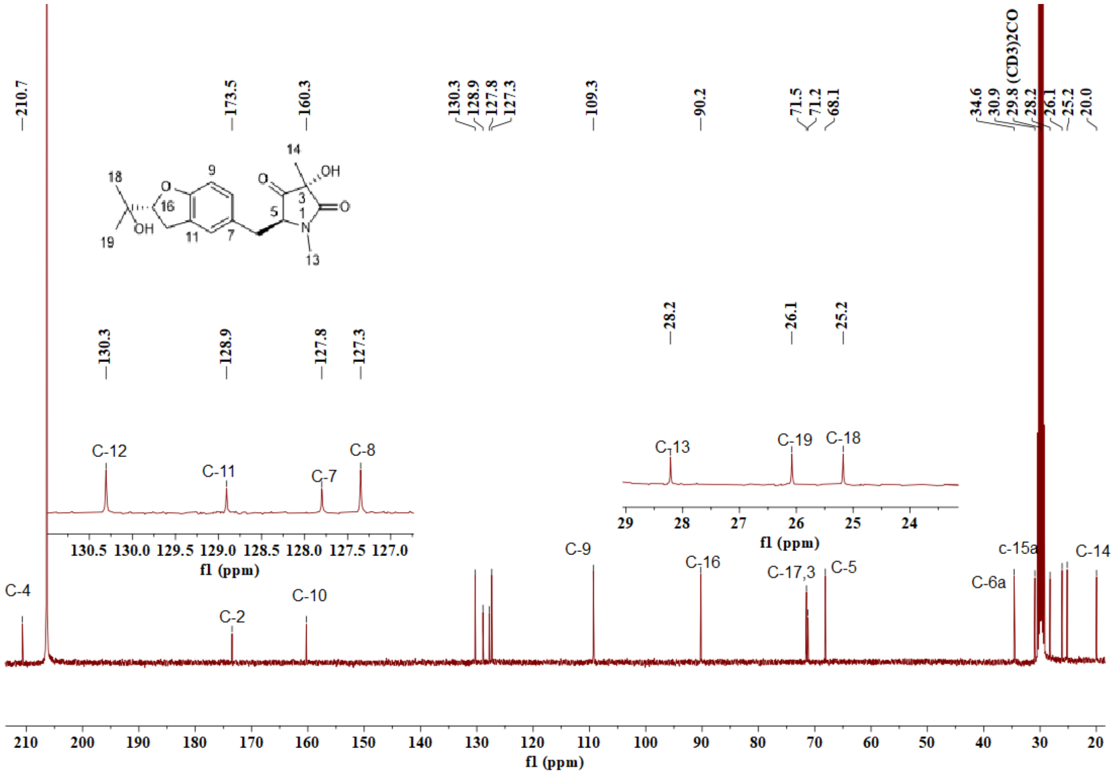

Supplementary Figure 30. The ^{13}C NMR (100 MHz, acetone-d_6) spectrum of compound **8**.

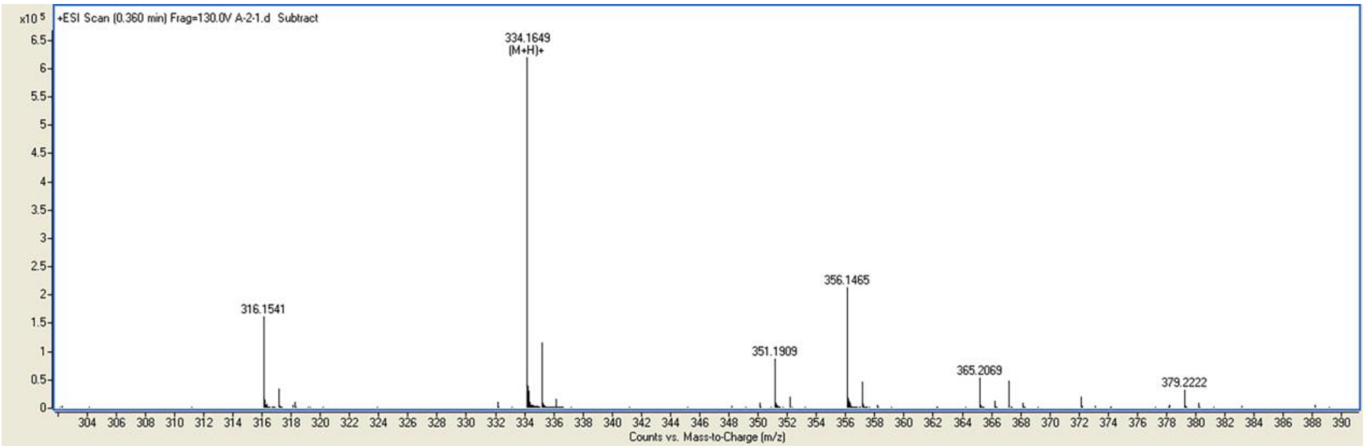

Supplementary Figure 31. The ESI-HRMS spectrum of compound **8**.

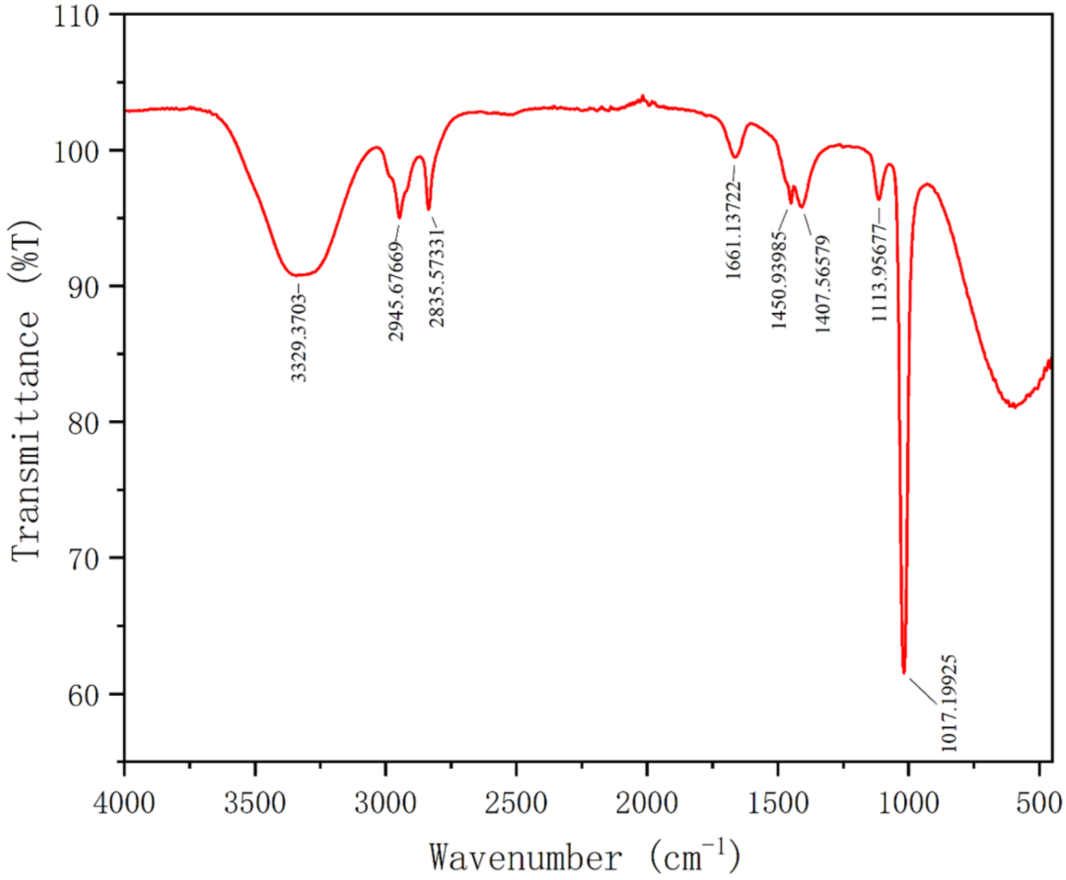

Supplementary Figure 32. IR spectrum of compound **8**.

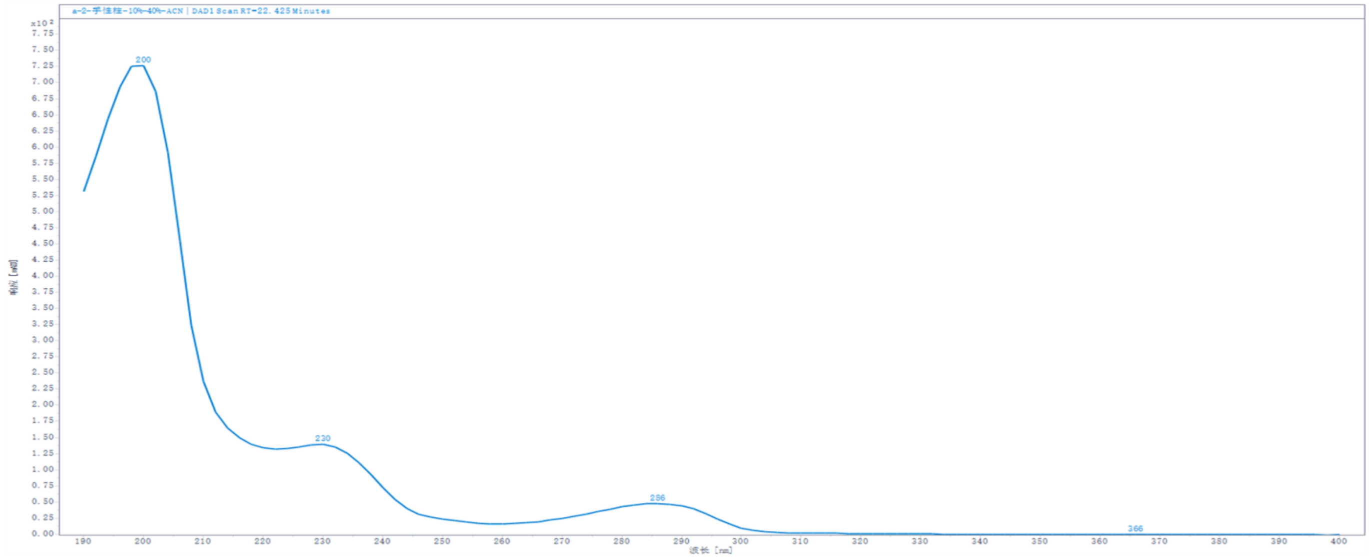

Supplementary Figure 33. UV spectrum of compound **8**.

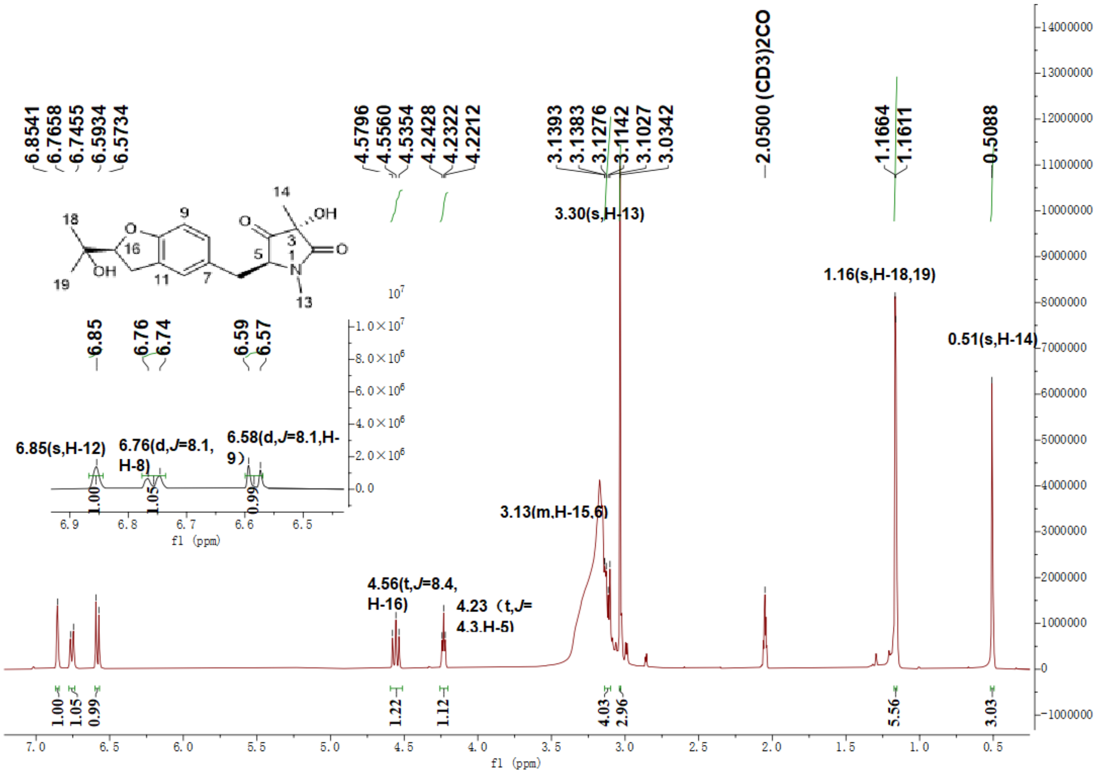

Supplementary Figure 34. The ^{1}H NMR (400 MHz, acetone-d_6) spectrum of compound **9**.

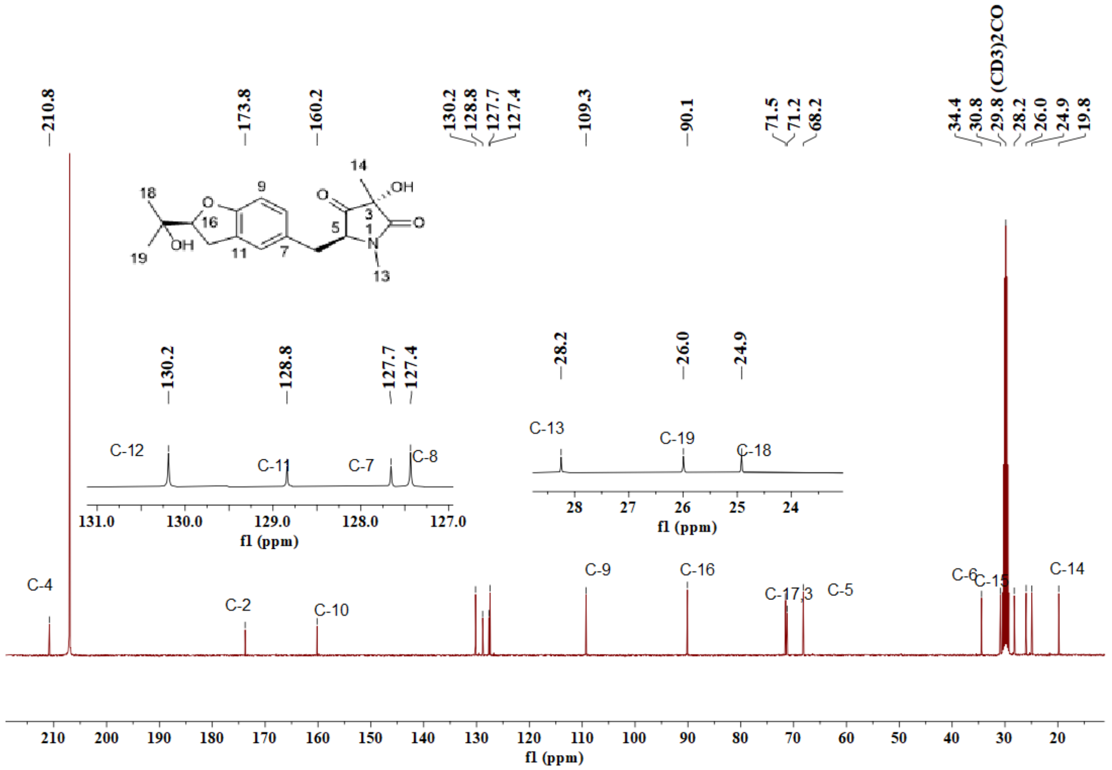

Supplementary Figure 35. The ^{13}C NMR (100 MHz, acetone-d_6) spectrum of compound **9**.

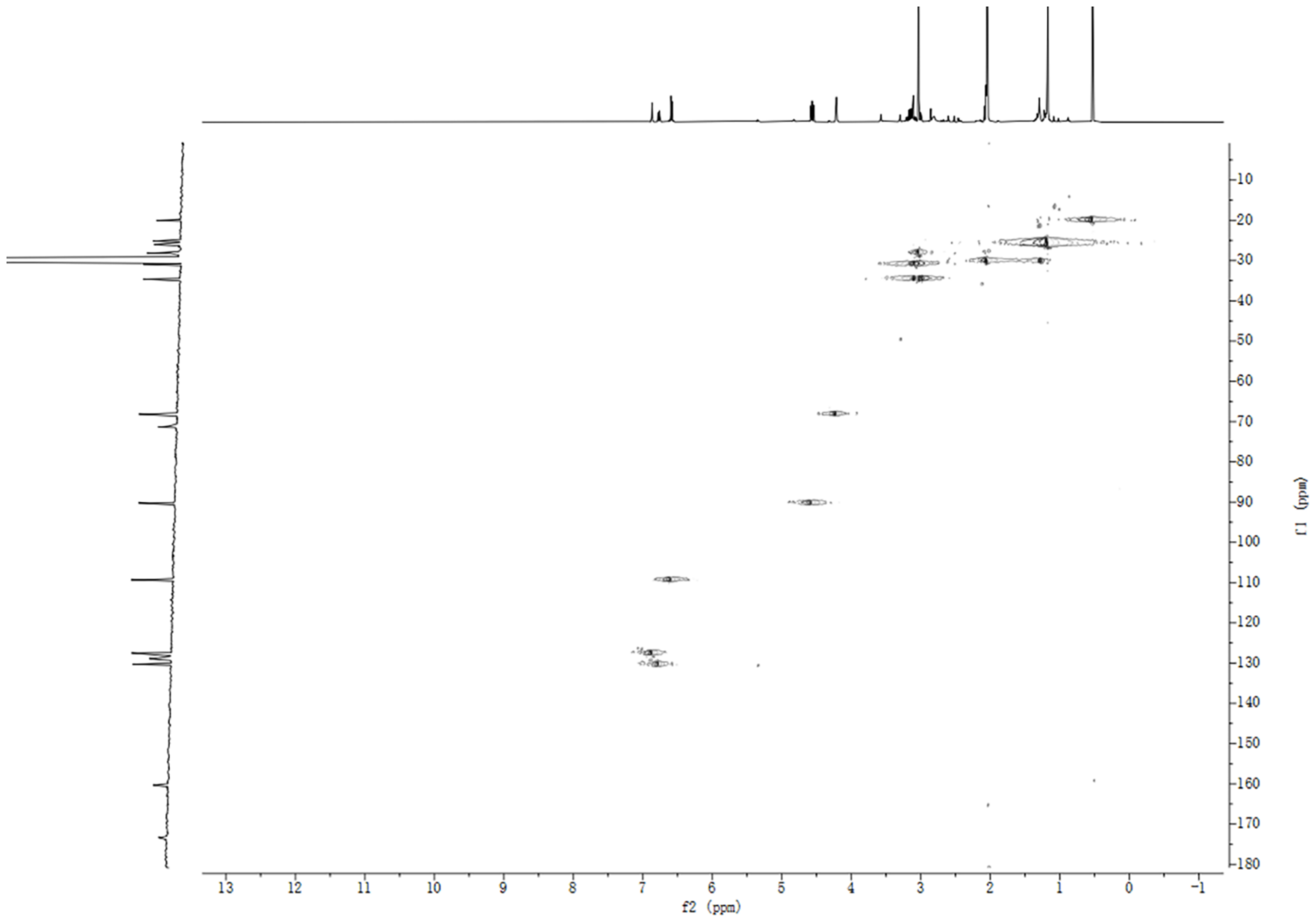

Supplementary Figure 36. The HSQC spectrum of compound **9**.

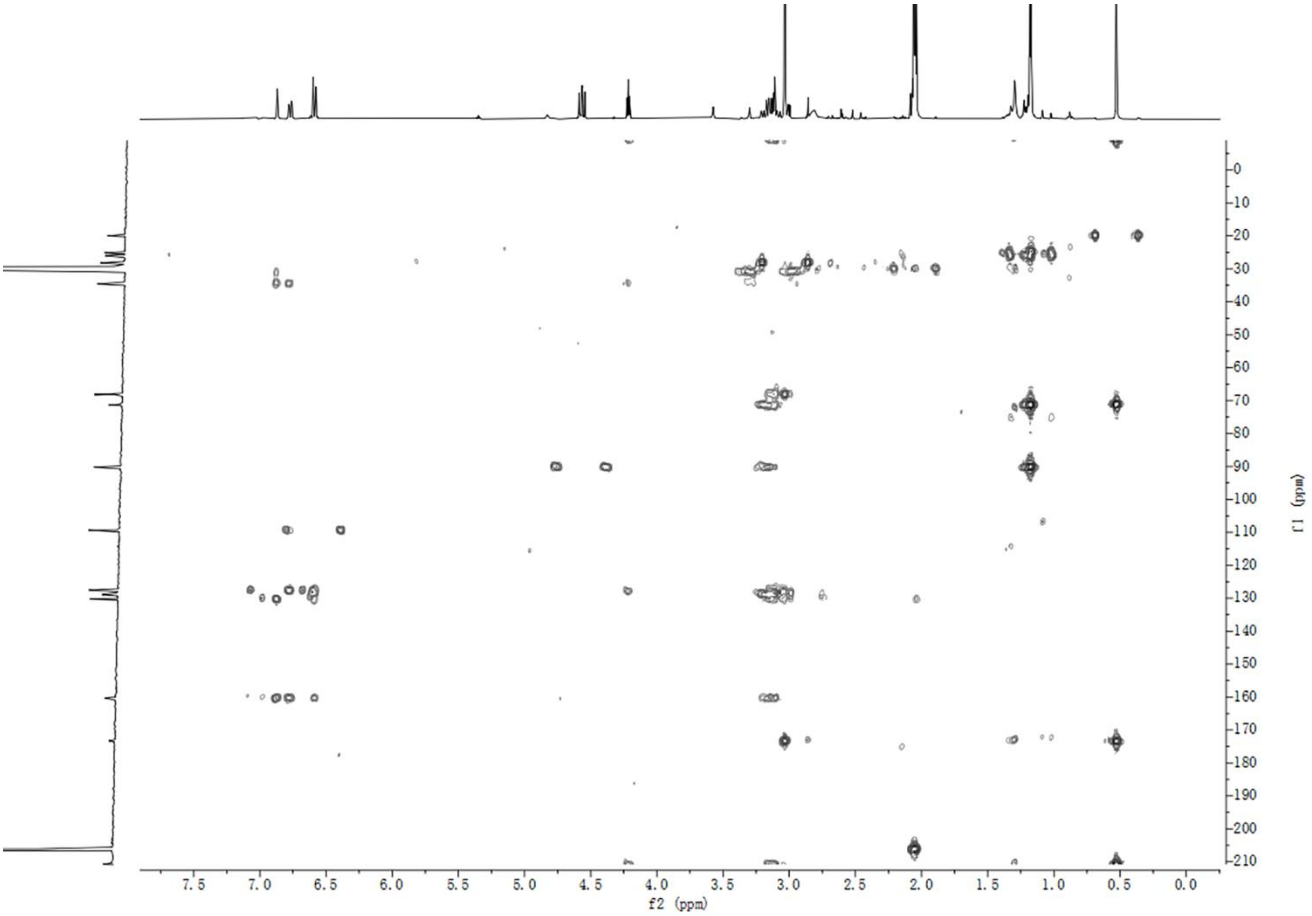

Supplementary Figure 37. The HMBC spectrum of compound **9**.

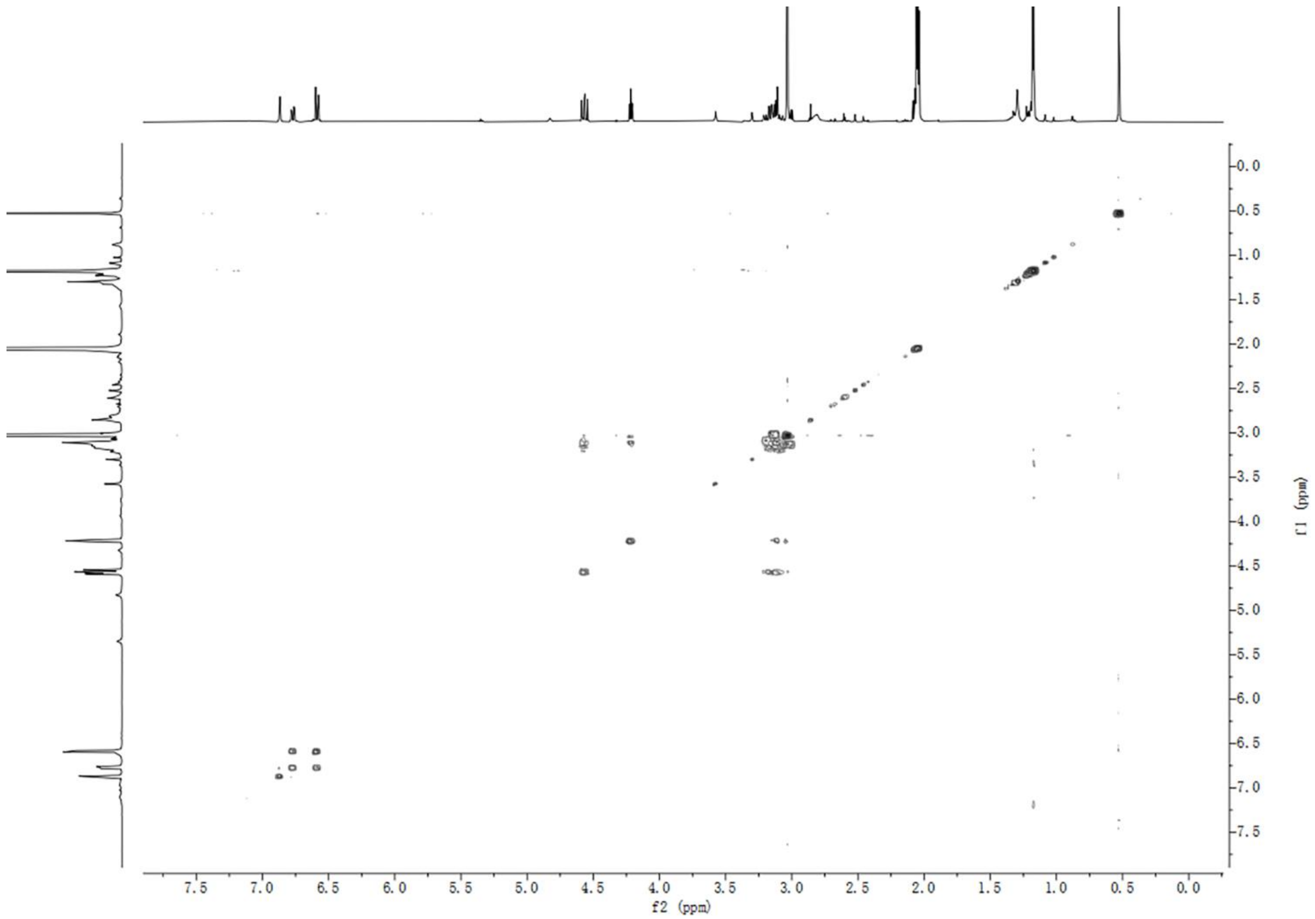

Supplementary Figure 38. The COSY spectrum of compound **9**.

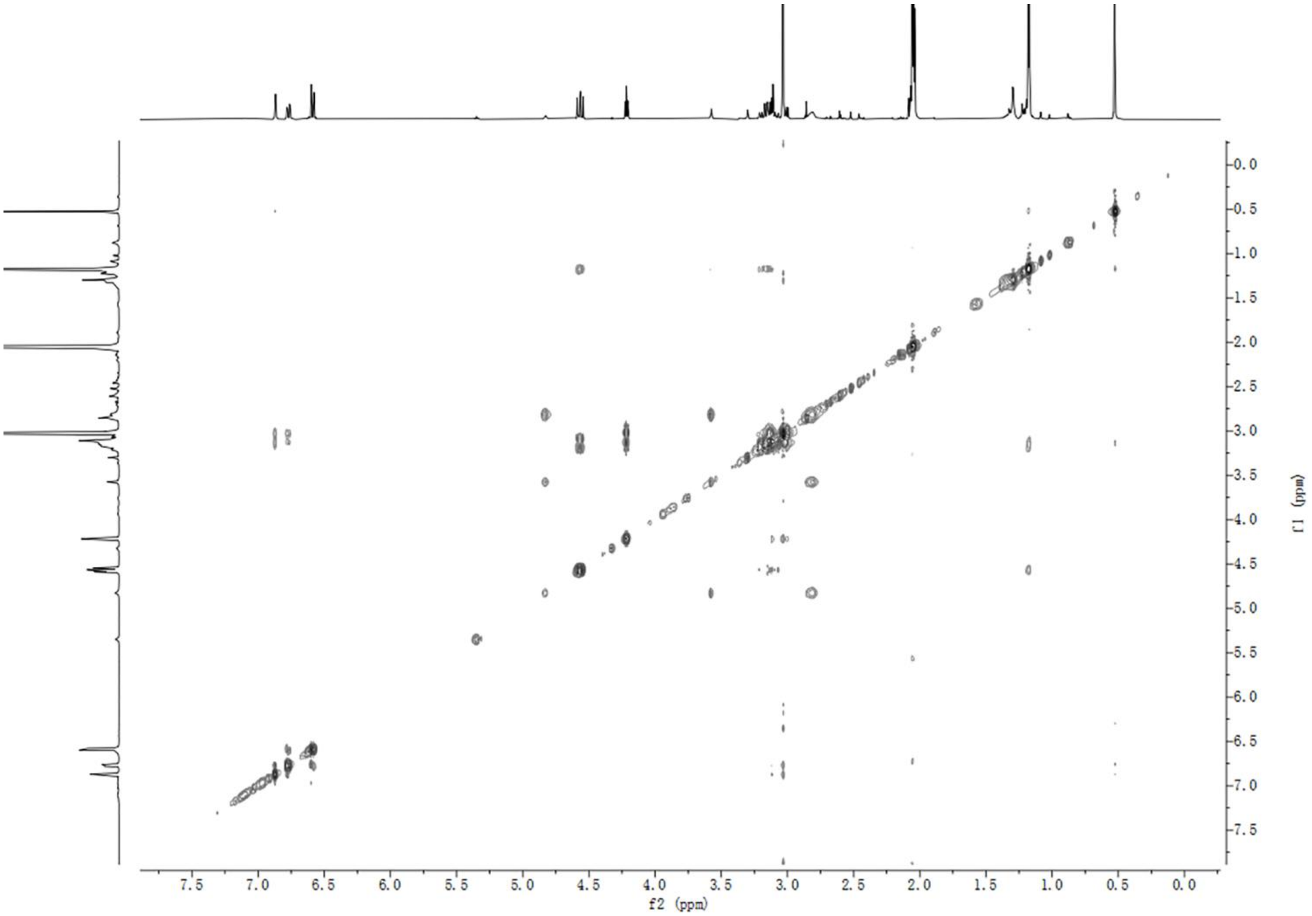

Supplementary Figure 39. The NOESY spectrum of compound **9**.

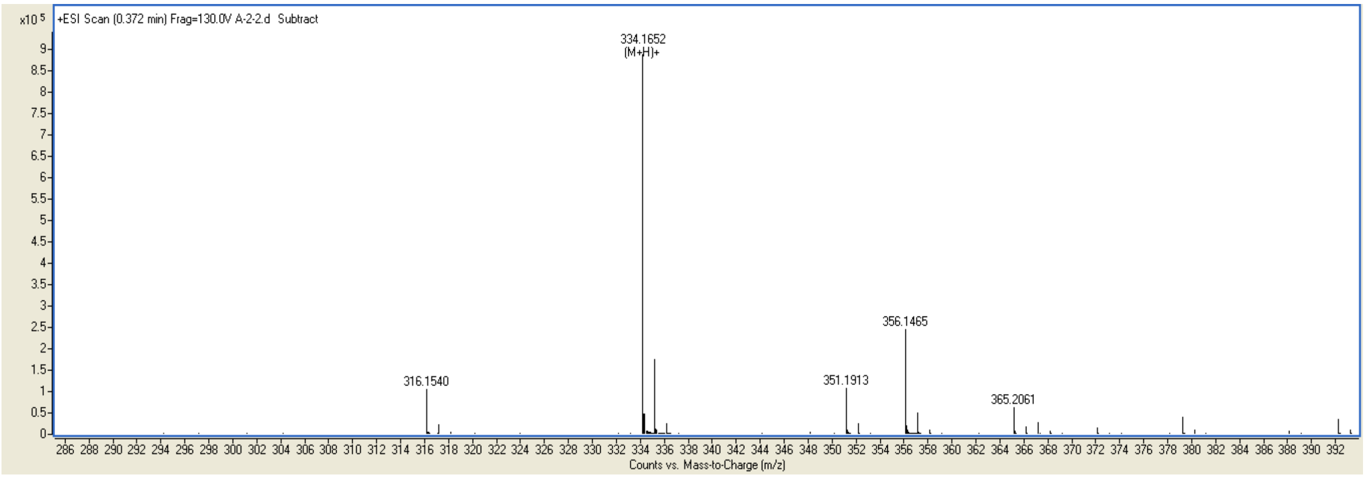

Supplementary Figure 40. The ESI-HRMS spectrum of compound **9**.

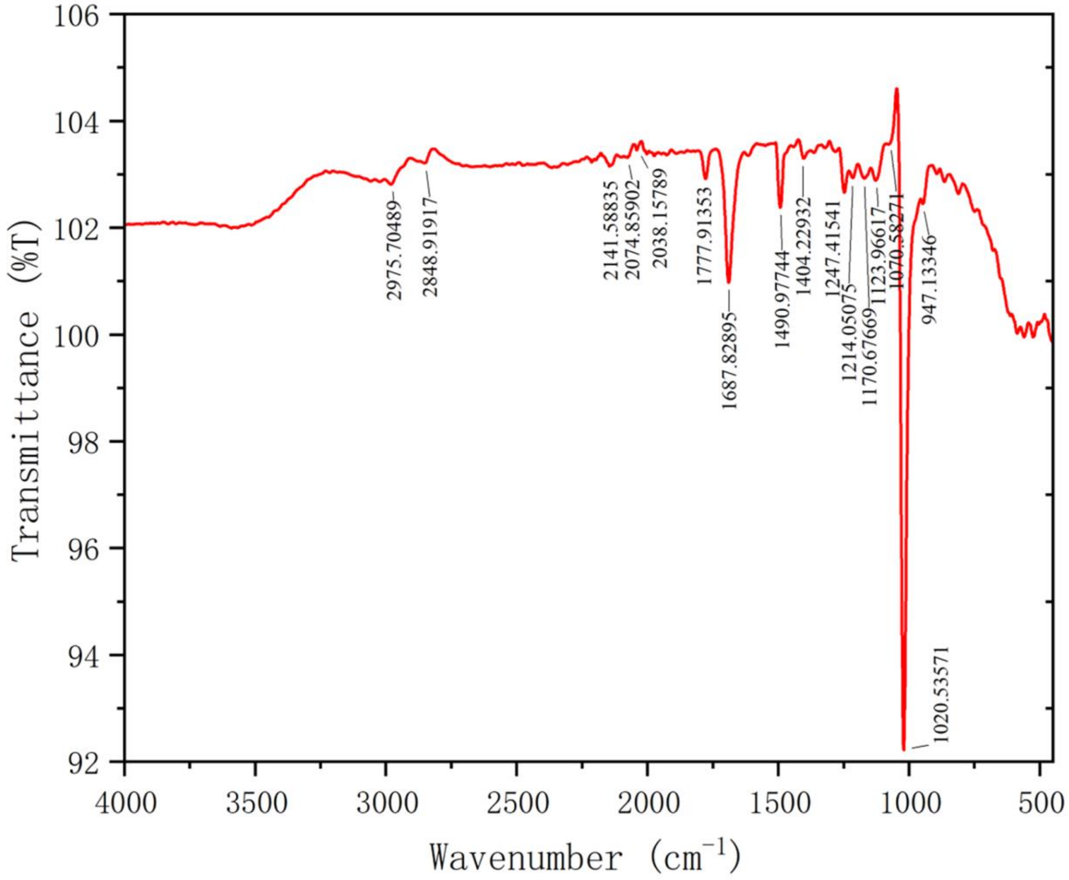

Supplementary Figure 41. IR spectrum of compound **9**.

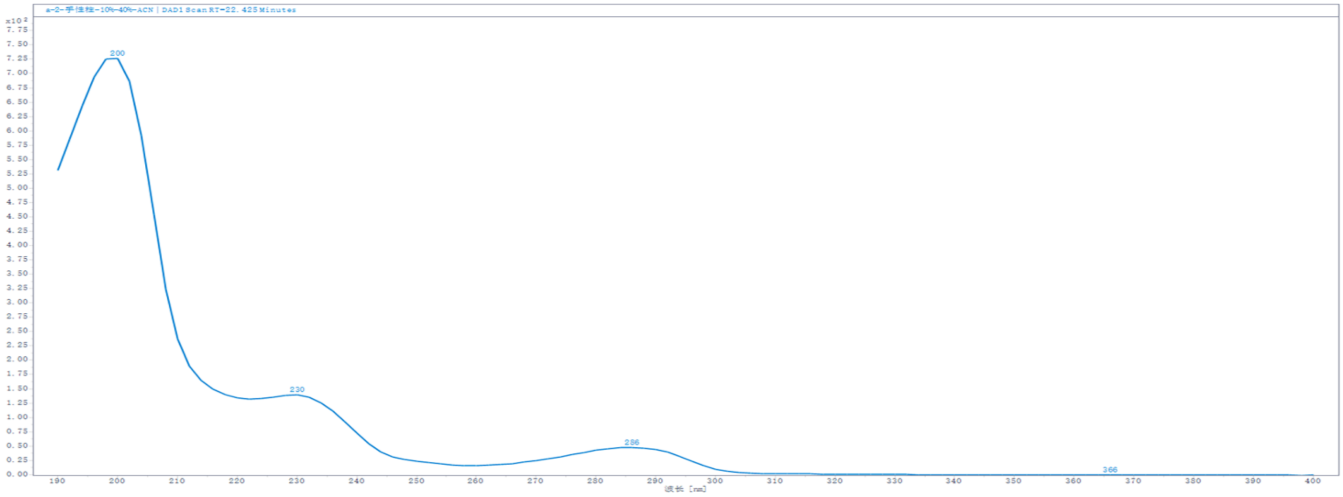

Supplementary Figure 42. UV spectrum of compound **9**.

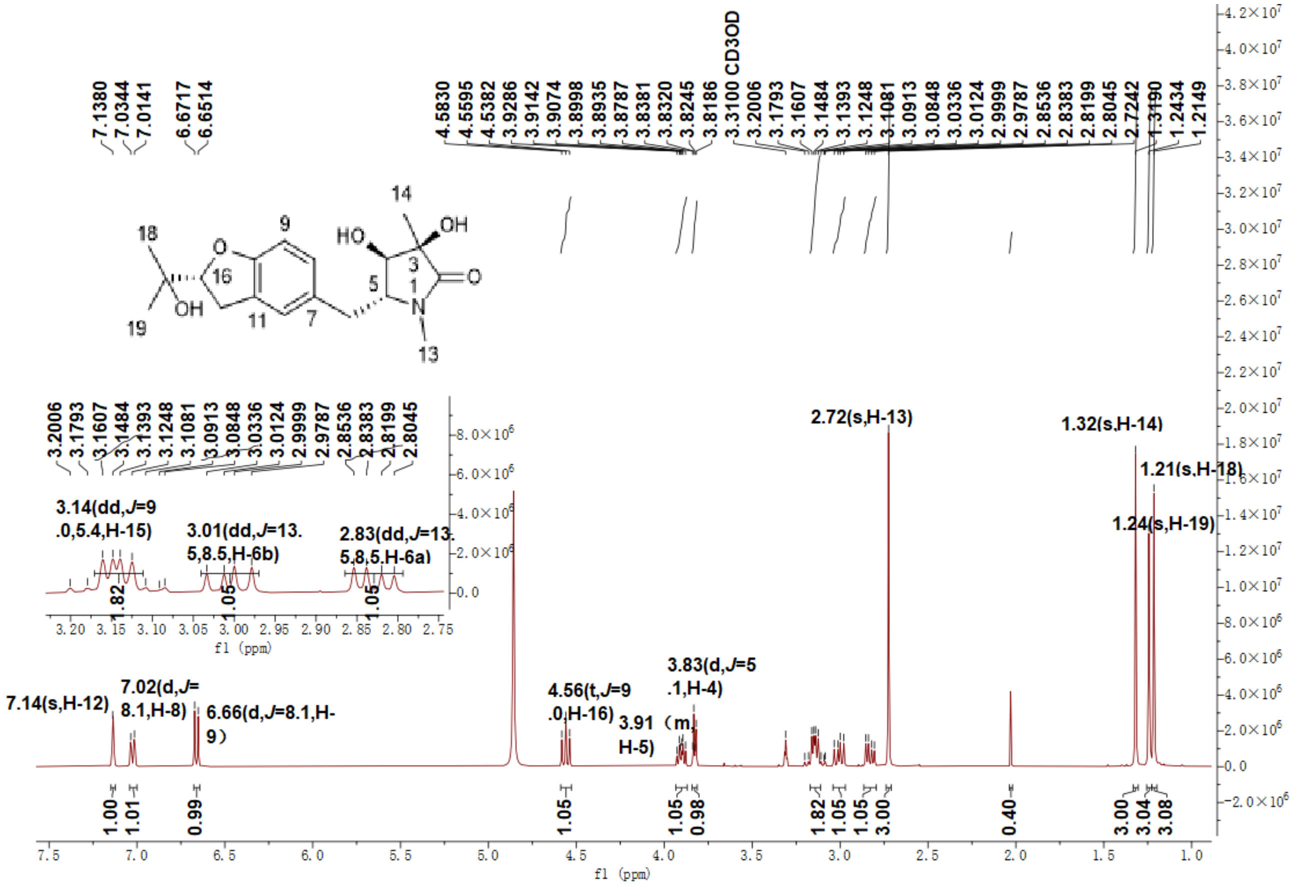

Supplementary Figure 43. The ^{1}H NMR (400 MHz, methanol-d_4) spectrum of compound **10**.

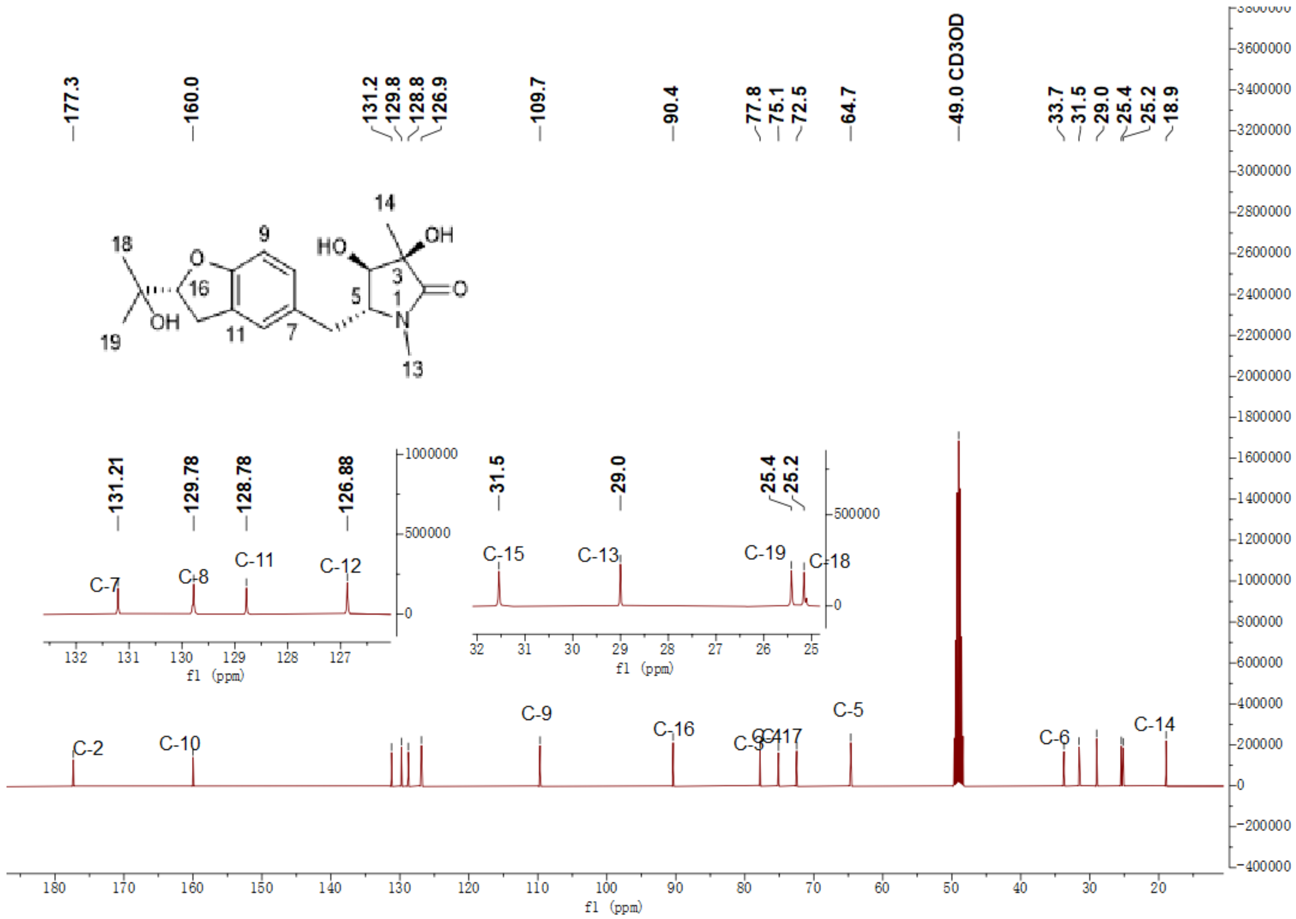

Supplementary Figure 44. The ^{13}C NMR (100 MHz, methanol-d_4) spectrum of compound **10**.

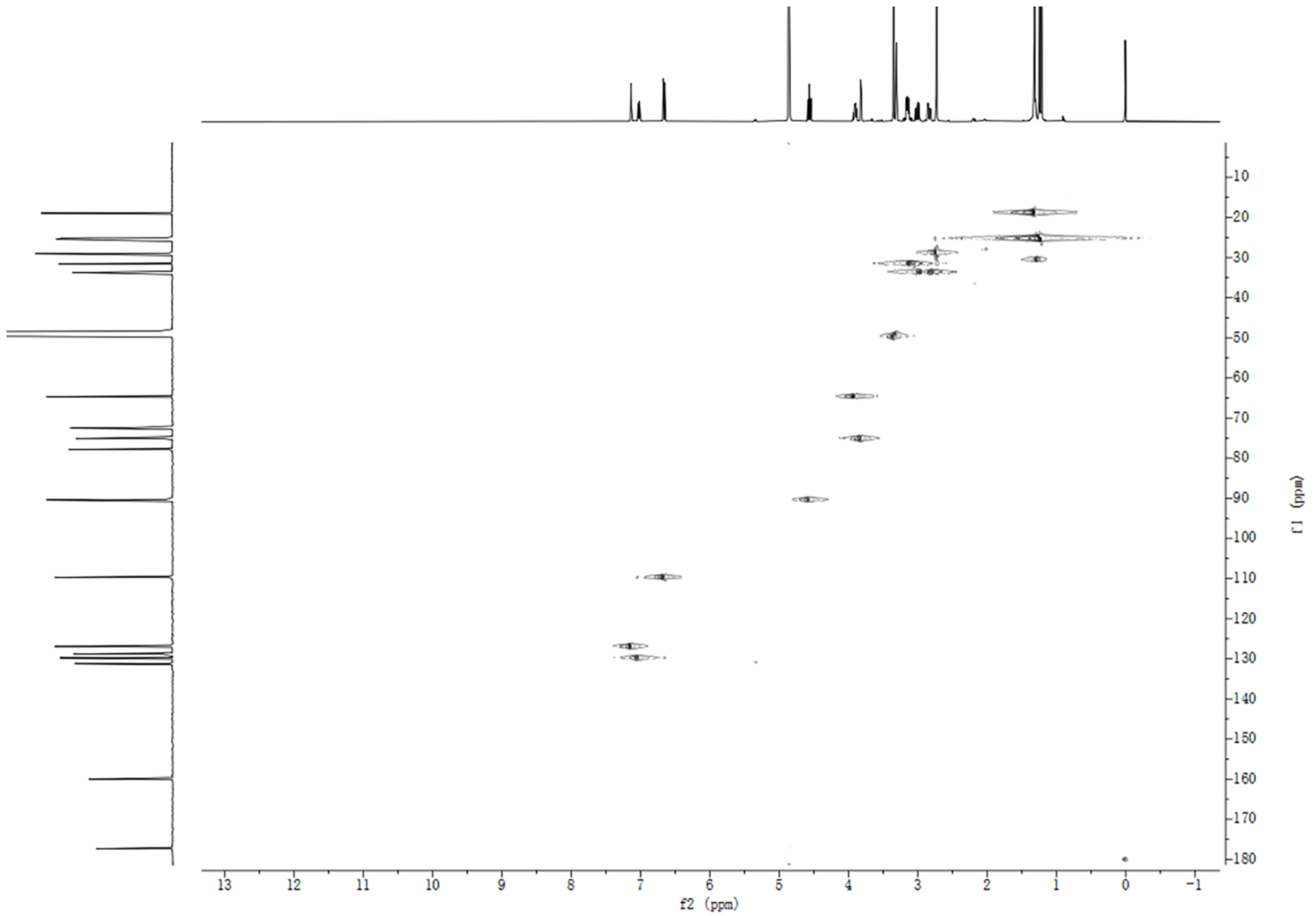

Supplementary Figure 45. The HSQC spectrum of compound **10**.

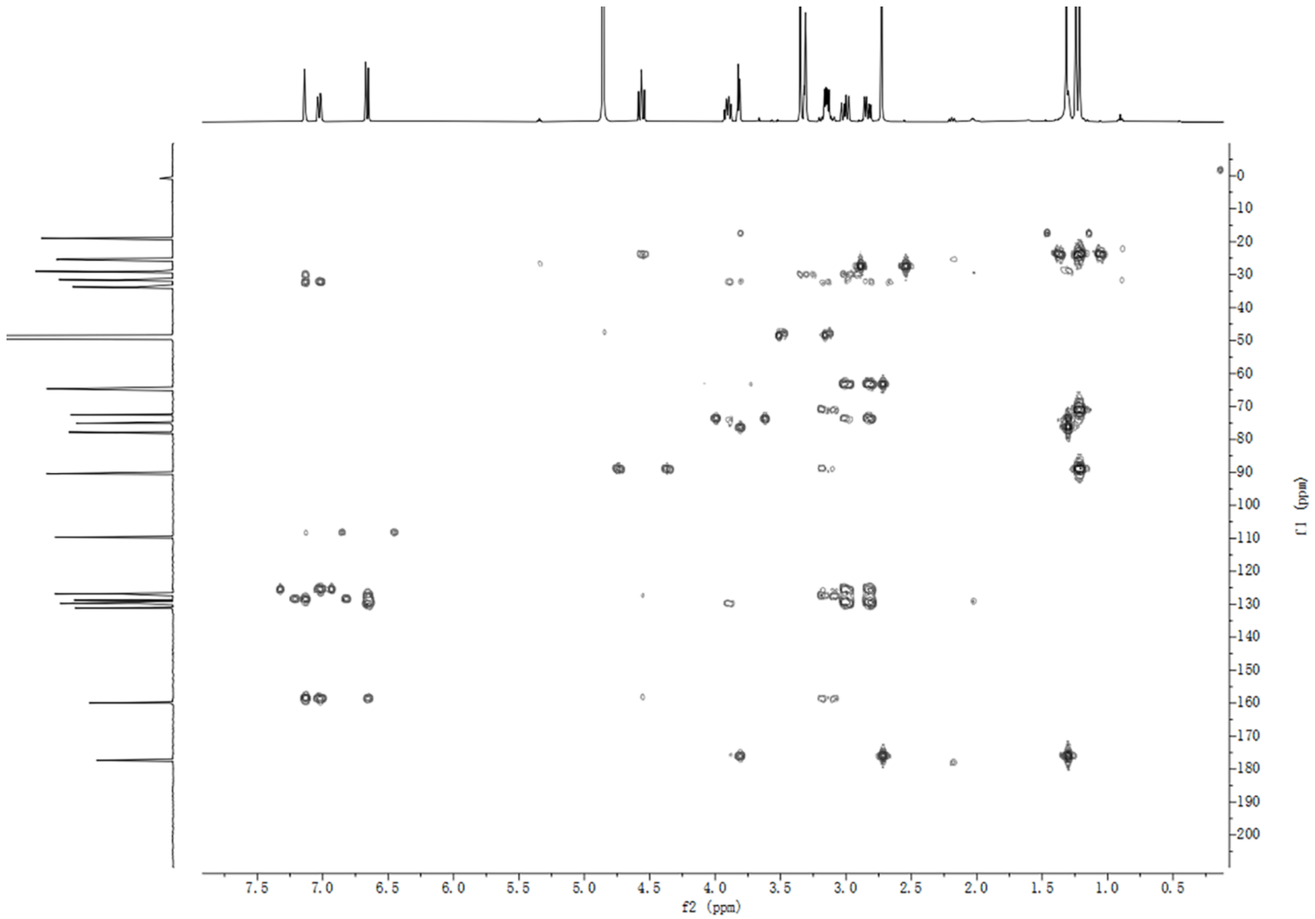

Supplementary Figure 46. The HMBC spectrum of compound **10**.

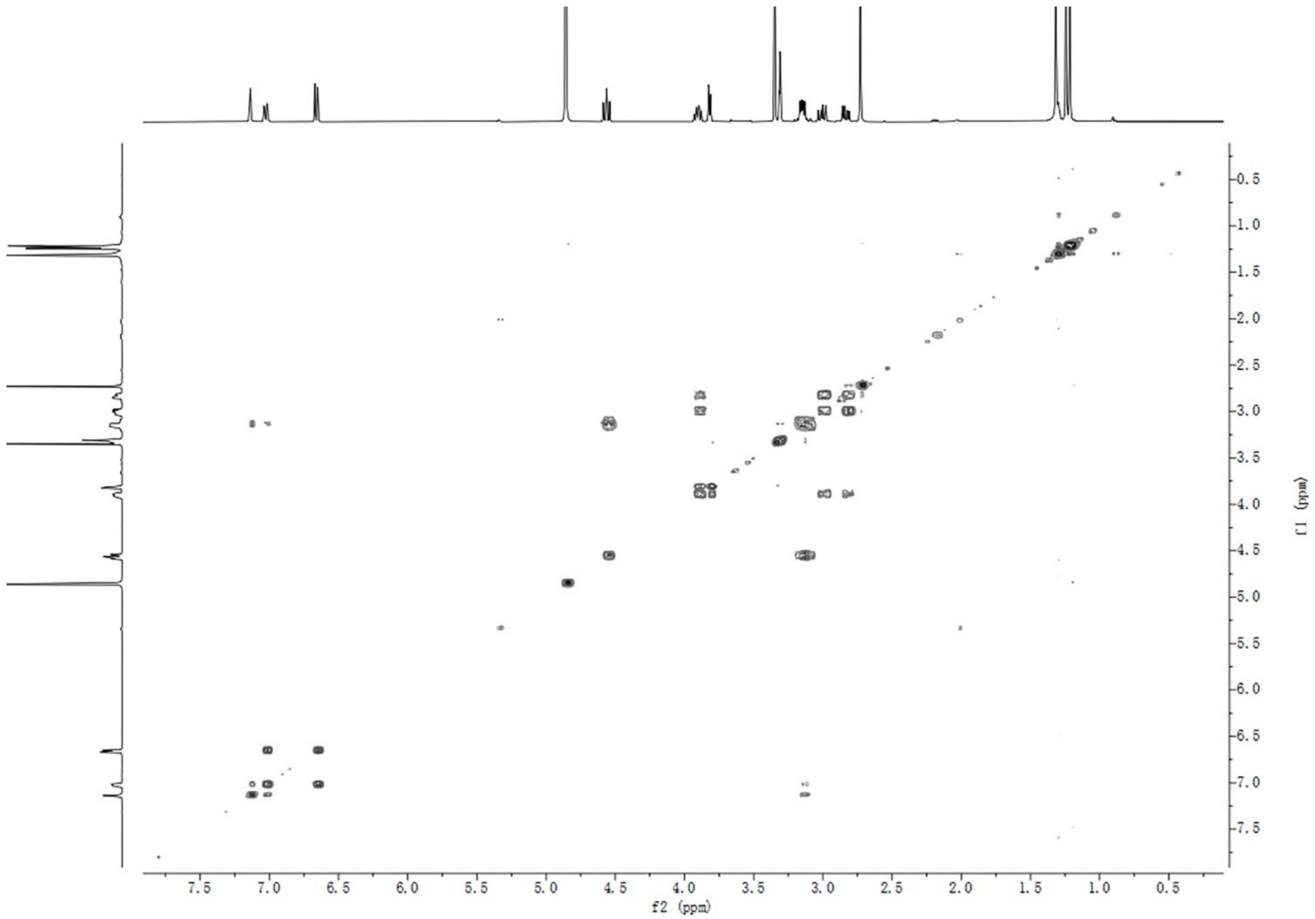

Supplementary Figure 47. The COSY spectrum of compound **10**.

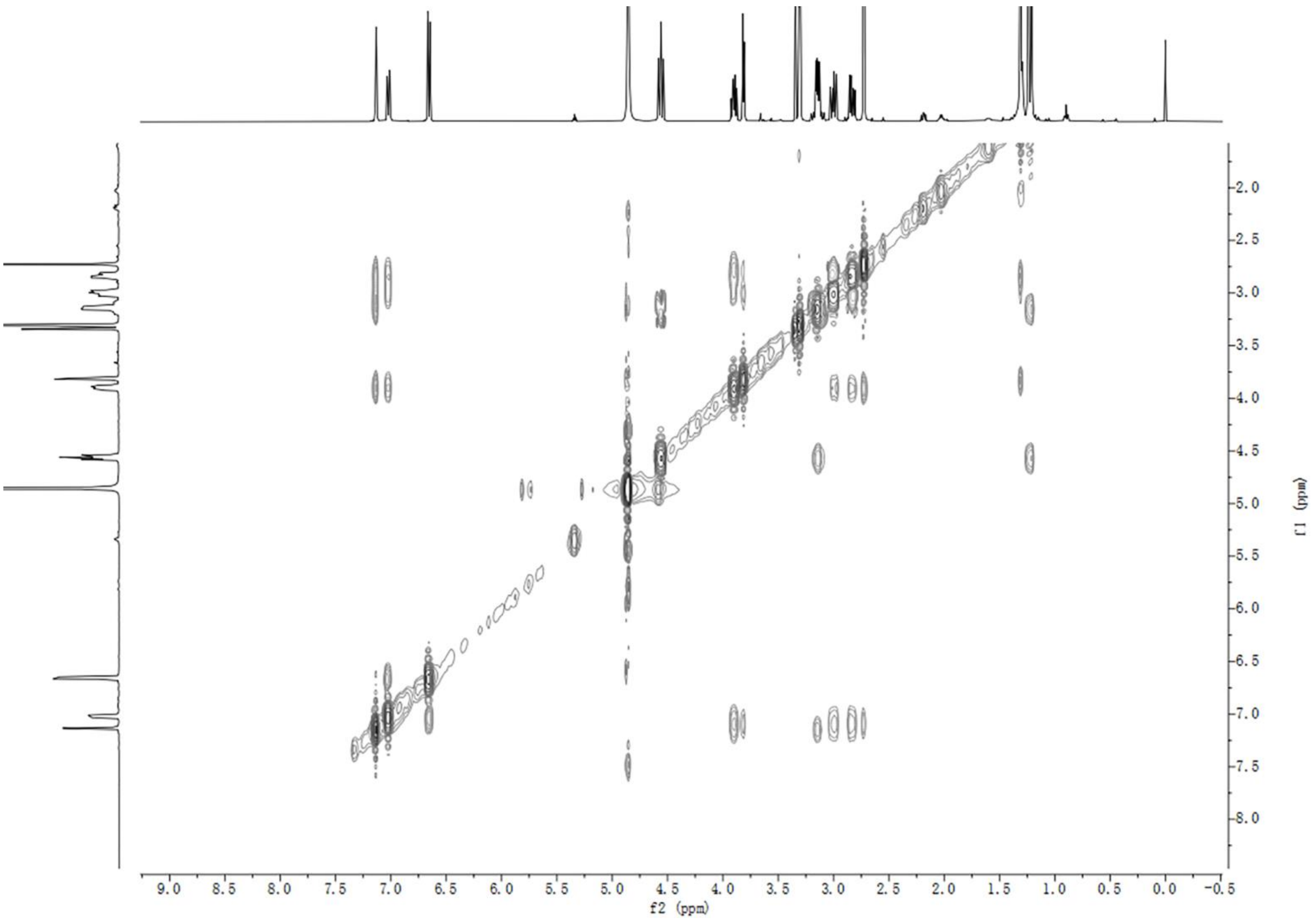

Supplementary Figure 48. The NOESY spectrum of compound **10**.

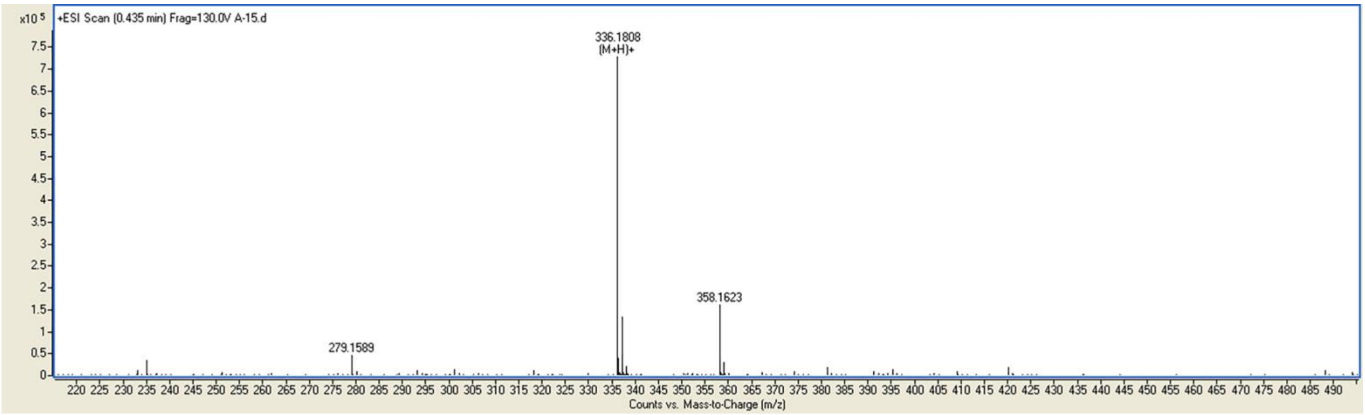

Supplementary Figure 49. The ESI-HRMS spectrum of compound **10**.

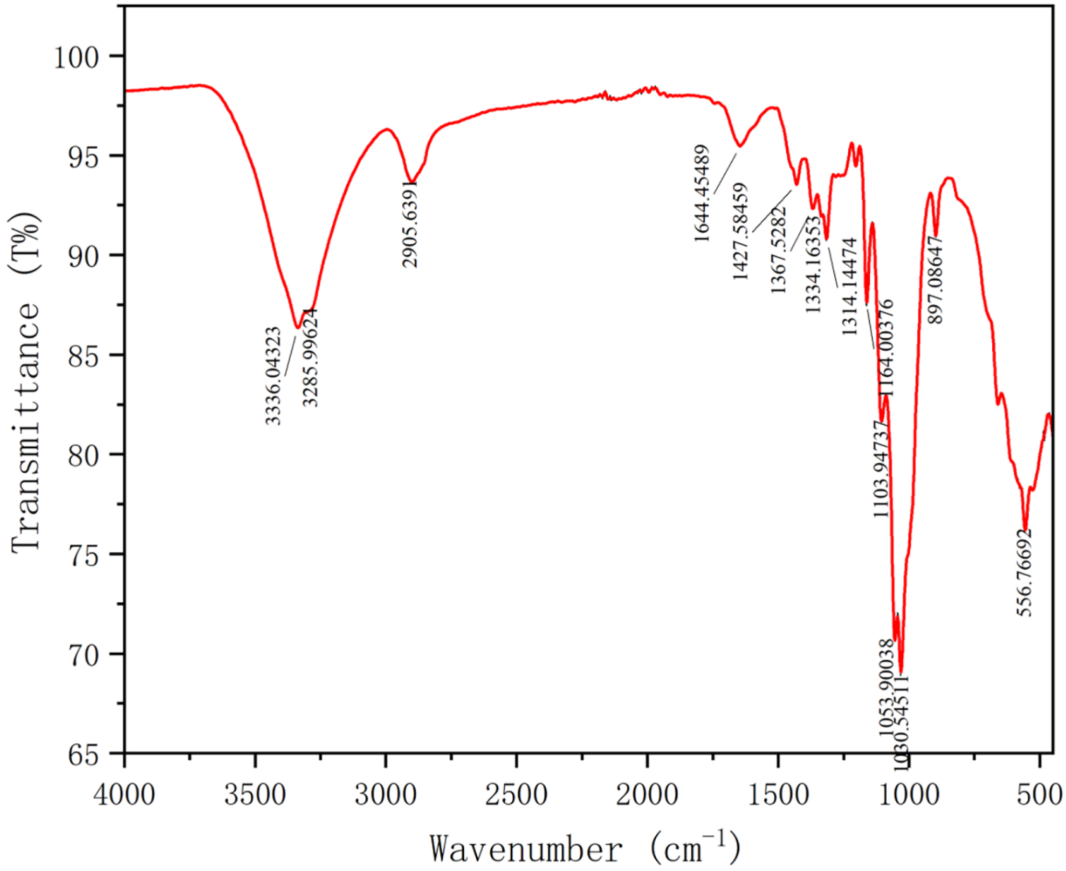

Supplementary Figure 50. IR spectrum of compound **10**.

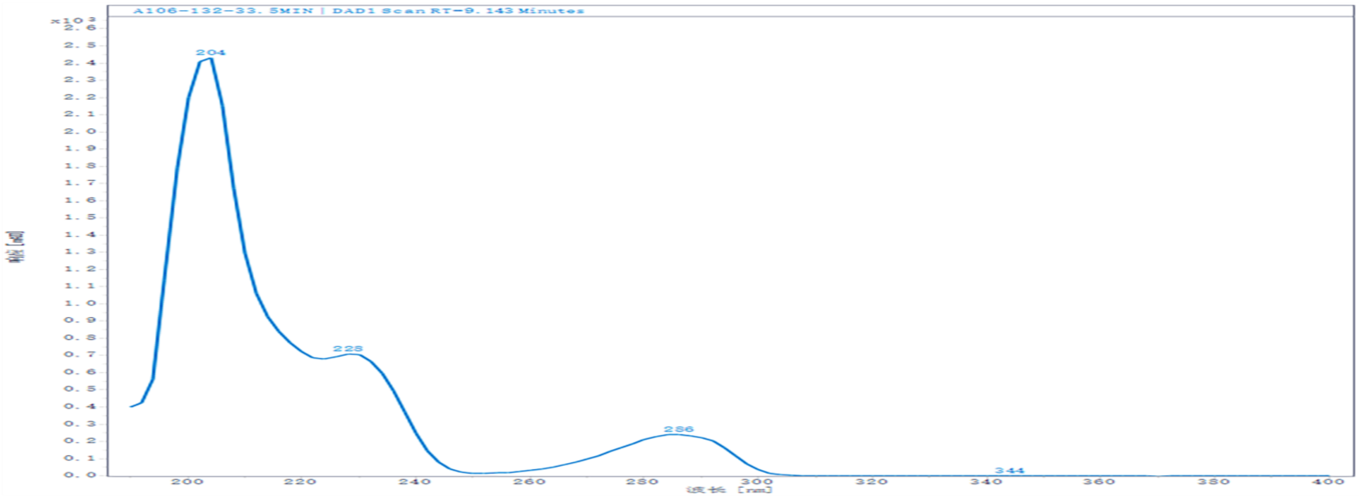

Supplementary Figure 51. UV spectrum of compound **10**.

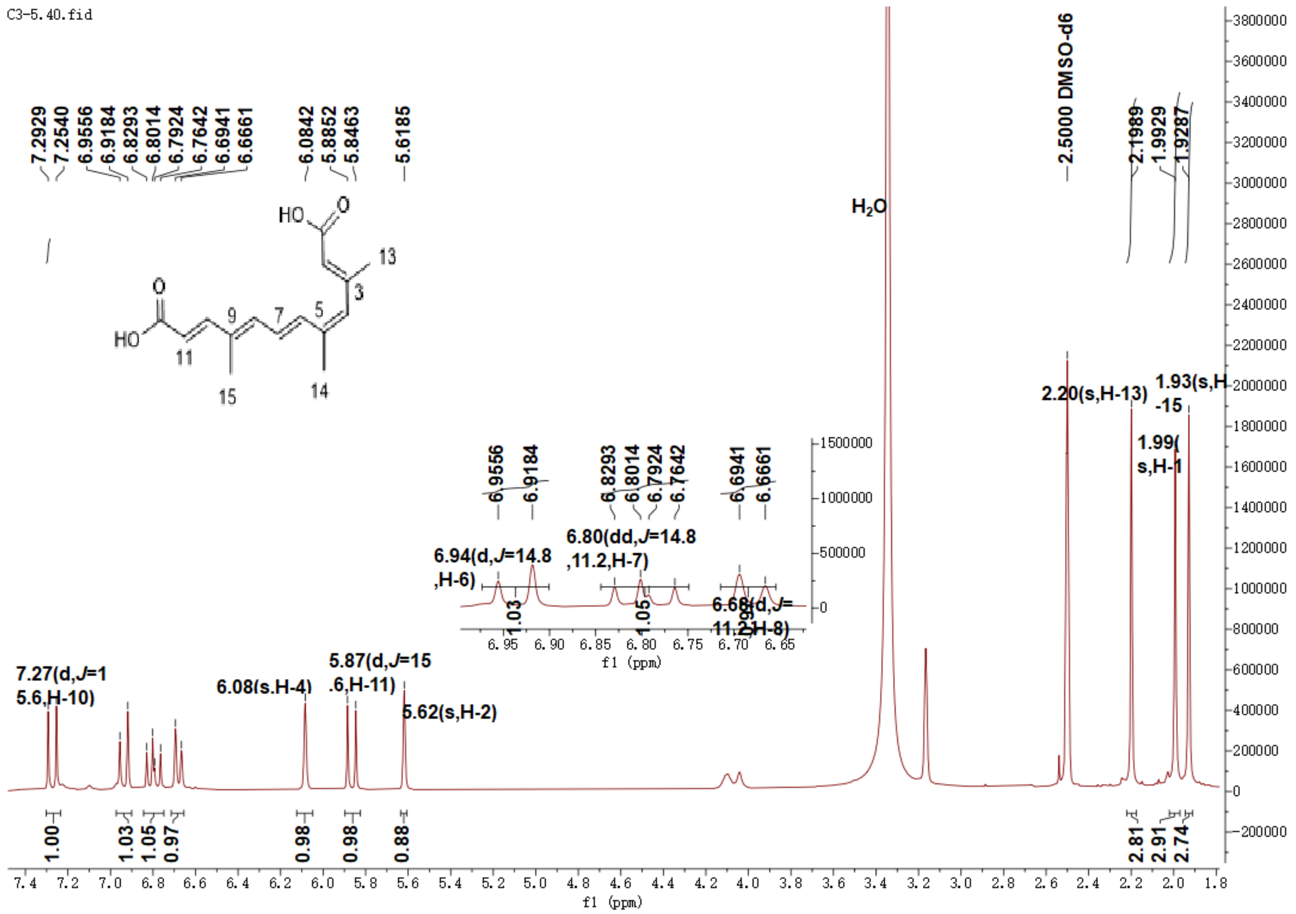

Supplementary Figure 52. The ^{1}H NMR (400 MHz, DMSO-d_6) spectrum of compound **15**.

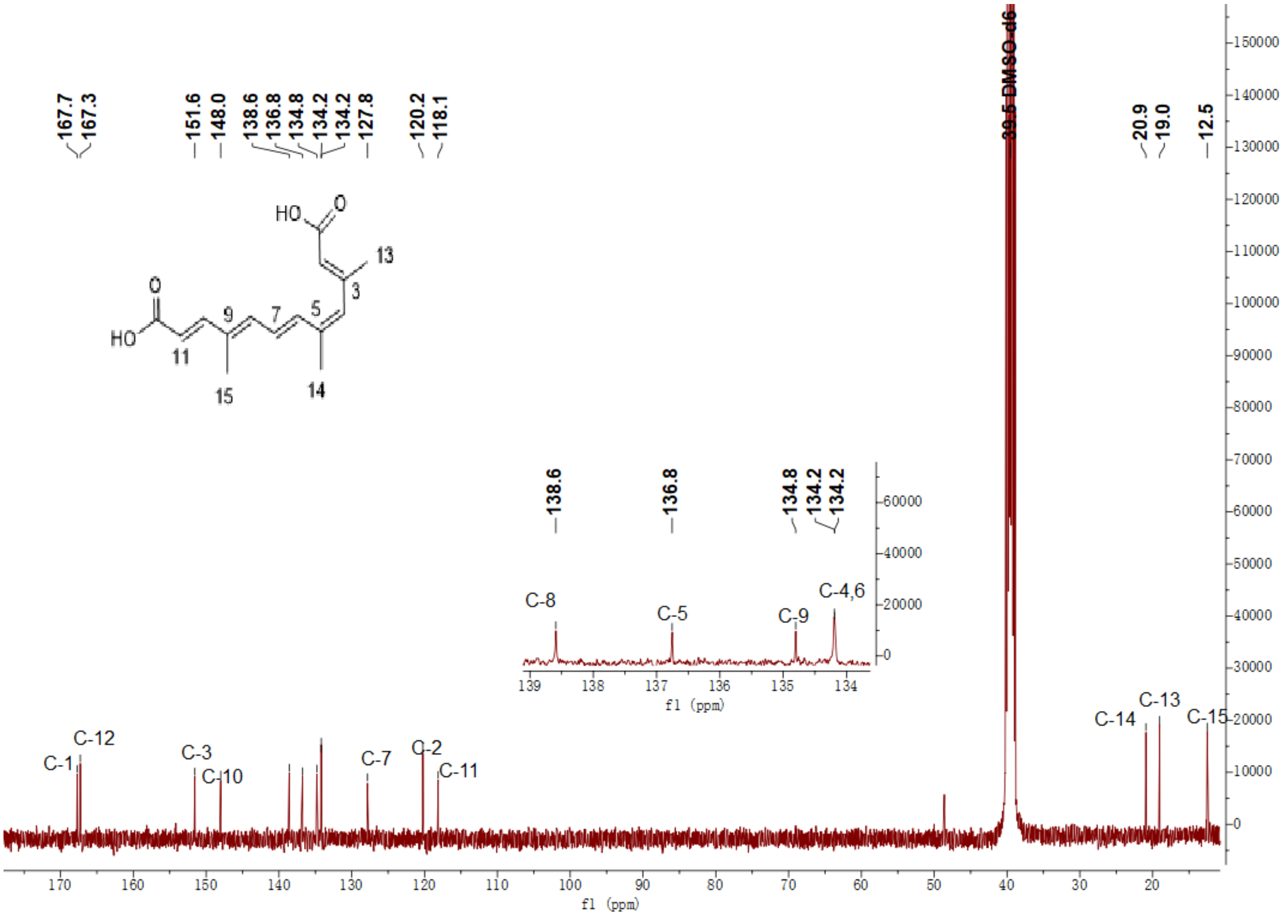

Supplementary Figure 53. The ^{13}C NMR (100 MHz, DMSO-d_6) spectrum of compound **15**

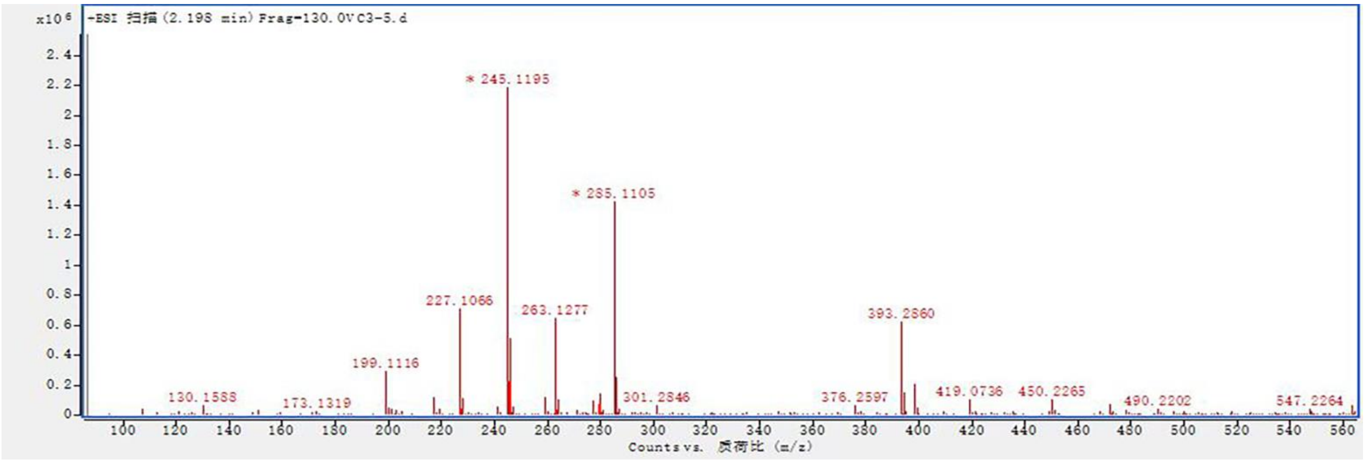

Supplementary Figure 54. The ESI-HRMS spectrum of compound **15**.

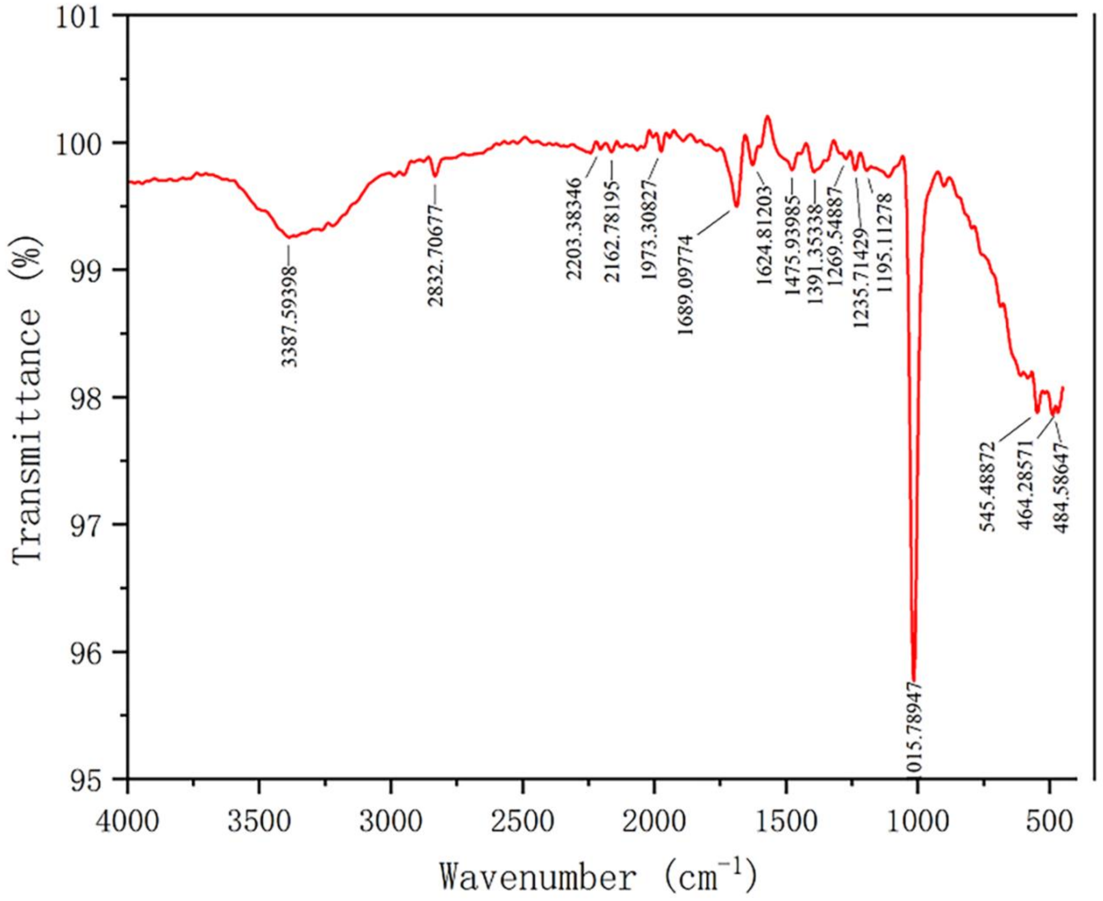

Supplementary Figure 55. IR spectrum of compound **15**.

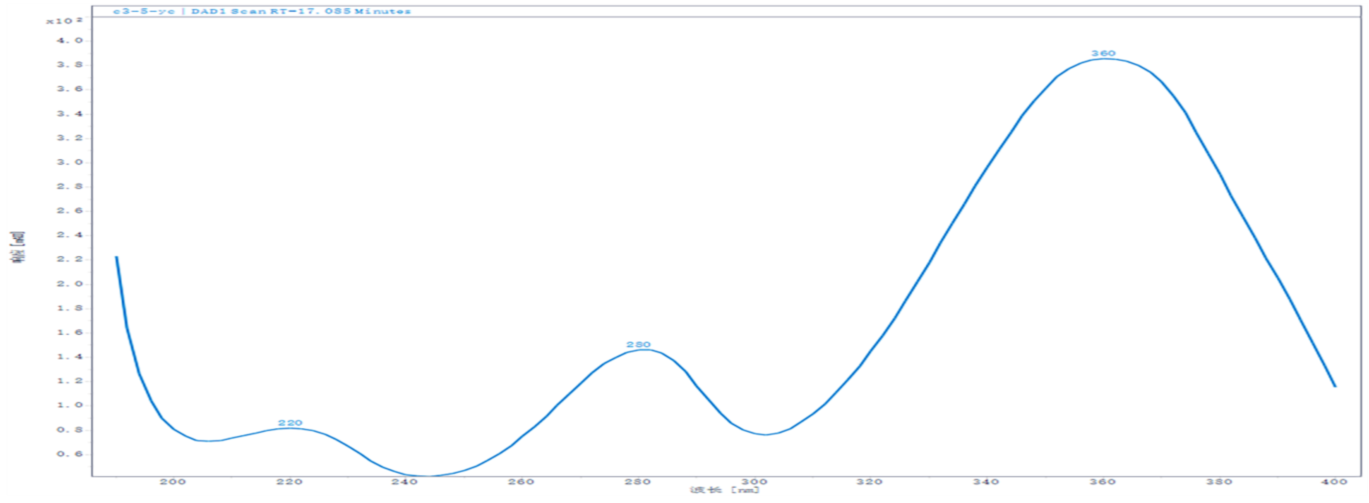

Supplementary Figure 56. UV spectrum of compound **15**.
